# A Minimal, Unstrained S‐Allyl Handle for Pre‐Targeting Diels–Alder Bioorthogonal Labeling in Live Cells

**DOI:** 10.1002/anie.201608438

**Published:** 2016-10-20

**Authors:** Bruno L. Oliveira, Zijian Guo, Omar Boutureira, Ana Guerreiro, Gonzalo Jiménez‐Osés, Gonçalo J. L. Bernardes

**Affiliations:** ^1^ Department of ChemistryUniversity of CambridgeLensfield RoadCB2 1EWCambridgeUK; ^2^ Instituto de Medicina MolecularFaculdade de MedicinaUniversidade de LisboaAvenida Professor Egas Moniz1649-028LisboaPortugal; ^3^ Departamento de QuímicaUniversidad de La RiojaCentro de Investigación en Síntesis Química26006LogroñoSpain; ^4^ Institute of Biocomputation and Physics of Complex Systems (BIFI)University of ZaragozaBIFI-IQFR (CSIC)ZaragozaSpain

**Keywords:** apoptosis, bioorthogonal labeling, Diels–Alder reactions, pre-targeting, unstrained alkenes

## Abstract

The unstrained S‐allyl cysteine amino acid was site‐specifically installed on apoptosis protein biomarkers and was further used as a chemical handle and ligation partner for 1,2,4,5‐tetrazines by means of an inverse‐electron‐demand Diels–Alder reaction. We demonstrate the utility of this minimal handle for the efficient labeling of apoptotic cells using a fluorogenic tetrazine dye in a pre‐targeting approach. The small size, easy chemical installation, and selective reactivity of the S‐allyl handle towards tetrazines should be readily extendable to other proteins and biomolecules, which could facilitate their labeling within live cells.

The inverse‐electron‐demand Diels–Alder reaction (IEDDA) between tetrazines and alkenes have recently found widespread application as an alternative “click” reaction for chemical biology applications due to its high chemoselectivity, fast reaction kinetics, and metal‐free nature.^1^ Most protein‐labeling approaches through IEDDA ligation rely on strained cyclic alkenes and alkynes, such as norbornene,^2^ *trans*‐cyclooctene (TCO),^3^ or bicyclo[6.1.0]nonyne,3b that are introduced on proteins by genetic encoding methods for subsequent reaction with tetrazines. Alternatively, tetrazines may also be genetically encoded into proteins and subsequently labeled with TCO.^4^ While these strained coupling partners reach very fast reaction kinetics with tetrazines (*k*_2_ up to 10^4^ m ^−1^ s^−1^),^5^ their metabolic stability and potential cross‐reactivity with biological nucleophiles is still arguable.^6^ In addition, their large size can potentially impact the protein structure, activity, or localization. Thus, there is a requirement for the development of reactive, yet stable and sterically small, alkene‐handles for tetrazine IEDDA labeling approaches.^7^ In one example, a small‐sized methylcyclopropene tag reacted with high selectivity and relative fast kinetics with tetrazines (*k*_2_≈0.1–3 m ^−1^ s^−1^).^8^ More recently, genetically encoded fatty acylated lysine (Lys) derivatives bearing unstrained alkenes were used to reveal Sirt6‐targeted histone H3 sites in nucleosomes upon reaction with tetrazines.^9^

The abovementioned approaches rely on genetic encoding of Lys derivatives bearing a reactive dienophile moiety. Instead, we thought to explore cysteine (Cys) as a site for the chemical installation of the dienophile counterpart. We chose S‐allyl as an unstrained, small‐sized alkene handle for reaction with tetrazines. The S‐allyl handle, when chemically installed on a surface‐exposed Cys on a protein or peptides, has proven to be an efficient partner for ruthenium‐catalyzed cross‐metathesis.^10^ The future development of genetic strategies to site‐specifically encode S‐allyl Cys, which is stable in the presence of biological thiols and is non‐toxic to cells up to 1 mm (HeLa and HepG2 cells, see the Supporting Information), may also potentiate their use in bioorthogonal labeling strategies.10b

Herein, we have explored S‐allyl Cys as a coupling amino acid partner for IEDDA reactions that proved to be reactive toward tetrazines with reasonable reaction kinetics (*k*_2_≈0.002 m ^−1^ s^−1^). We demonstrated that S‐allyl Cys could be easily chemically installed into apoptosis protein biomarkers that specifically recognize the externalized phosphatidylserine (PS) membrane lipid through a [2,3]‐sigmatropic rearrangement with allyl selenocyanate.^11^ Using a pre‐targeting approach, and upon reaction with a fluorogenic tetrazine, efficient live‐imaging of apoptotic cells was achieved. The easy chemical installation of the S‐allyl handle and availability of protein and antibody biomarkers, which have already been engineered with a free Cys residue, combined with the specificity and favorable kinetics of the IEDDA reaction, makes this a readily adoptable labeling strategy for pre‐targeting approaches.^12^

We started our study by investigating the reactivity of the unstrained alkene handle in IEDDA reactions using S‐allyl Cys amino acid **1** as a model (Figure 1 a,b). Reaction kinetics between **1** and tetrazines PyTz **2**, BnNH_2_‐Tz **3**, Tz‐Rhod **4**, and Tz‐Cy3 **5** were determined in phosphate‐buffered saline (PBS) pH 7.4/methanol (1:1) at 37 °C (Figure 1 b–d). The pyridine–tetrazine and benzylamine–tetrazine cores were selected due to their high reactivity in Diels–Alder reactions and high in vitro stability.^13^ This model amino acid **1** and tetrazine Tz‐Rhod **4** were synthesized as described in the Supporting Information. Kinetic studies were carried out under pseudo‐first‐order conditions in which the alkene concentration was at least 375‐fold higher than the concentration of the tetrazines. The determined second‐order reaction rate constants are presented in Figure 1 e. For the tetrazine cores **2** and **3**, the reactions were monitored by following the decrease of the tetrazine absorbance at 320 nm (Figure 1 f). As shown in Figure 1, Py‐Tz **2** exhibited a slightly higher rate constant (*k*_2_=2.05×10^−3^ m ^−1^ s^−1^) than BnNH_2_‐Tz **3** (*k*_2_=0.54×10^−3^ m ^−1^ s^−1^). These values are lower than those reported for strained alkenes,^5^ but are directly comparable to those of, for example, strain‐promoted azide–alkyne cycloaddition “SPAAC” (*k*_2_≈10^−3^–10^−1^ m ^−1^ s^−1^) that is widely used for cellular labeling purposes.^5^, ^14^ To demonstrate that the tetrazine‐dye probes Tz‐Rhod **4** and Tz‐Cy3 **5** also react efficiently with **1**, their kinetics were also evaluated. In this case, conjugation of rhodamine and Cy3 moieties to the tetrazine cores results in fluorescence quenching. Cycloaddition of the tetrazine moieties and the alkene abolishes this intrinsic quenching effect, leading to an increased “turn‐on” of the fluorescence signal that could be readily detected by fluorimetry (Figure 1 g). The obtained rate constants calculated for these reactions (*k*_2_=0.34×10^−3^ m ^−1^ s^−1^ for **1**:Tz‐Rhod **4**; *k*_2_=0.26×10^−3^ m ^−1^ s^−1^ for **1**:Tz‐Cy3 **5**) are identical compared to the corresponding tetrazine cores. The smaller reactivity difference found for Tz‐Rhod **4** and Tz‐Cy3 **5** may be attributed to structural differences of the fluorophores. Kinetics studies for the reaction between 5‐norbornen‐2‐ol and Tz‐Cy3 **5** showed that the norbornene/tetrazine ligation (*k*_2_≈0.21 m ^−1^ s^−1^) is ≈800 times faster than the S‐allyl Cys/tetrazine ligation (Supporting Information). Similar reaction rates for comparable norbornene/tetrazine coupling partners have been reported.^15^ The fluorogenicity of the tetrazine‐dyes was also studied. It was observed that, upon cycloaddition, the fluorescence of **4** and **5** is enhanced by 5‐ and 2.5‐fold, respectively (Supporting Information).

**Figure 1:**
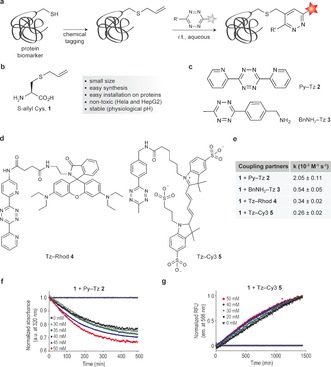
a) General reaction showing the application of IEDDA reactions for protein labeling with a site‐specifically installed S‐allyl handle. b–d) Structures of S‐allyl Cys, tetrazine cores, and fluorophores used in this study. e) Rate constants for the reaction of **2**–**5** with the model amino acid S‐allyl Cys **1** were measured following the consumption of tetrazines by UV/Vis (f) or fluorescence (g).

Encouraged by the kinetic analysis, we proceeded to synthesize proteins incorporating an S‐allyl handle for IEDDA labeling. As model proteins, we chose Annexin V (AnxV)^16^ and an engineered variant of the C2A domain of Synaptotagmin‐I (C2Am),^17^ two apoptosis‐specific biomarkers, that contain a free Cys residue. Because there are no other free Cys residues on these proteins, this provides a unique site for their site‐specific modification. The S‐allyl handle was installed on Cys through a [2,3]‐sigmatropic rearrangement using allyl selenocyanate after 1 h at room temperature (Figure 2 a,b).10c Direct allylation with allyl chloride was less efficient, with complete conversion to the product observed only after 3 h at 37 °C and using pH 11 (at pH 8 no product was detected; Supporting Information). It was also observed that these conditions resulted in significant protein loss (through degradation or precipitation) when compared to the allyl selenocyanate reaction (mass yields of 91 % and 48 % for allyl selenocyanate and allyl chloride reactions, respectively, as assessed by Bradford protein assay). S‐allyl‐tagged AnxV was then subjected to reaction with tetrazine cores Py‐Tz **2** and BnNH_2_‐Tz **3** (Figure 2 c,e). Product formation was detected (≈40 % conversion) by LC‐ESI‐MS after 20 and 12 h of reaction, respectively (complete reaction was obtained after 96 h for **2** and 36 h for **3**; Supporting Information). Reactions were monitored using Liquid Chromatography‐Electrospray Ionization Mass Spectrometry (LC‐ESI‐MS) analysis and Ellman's test (data for C2Am shown in the Supporting Information).

**Figure 2:**
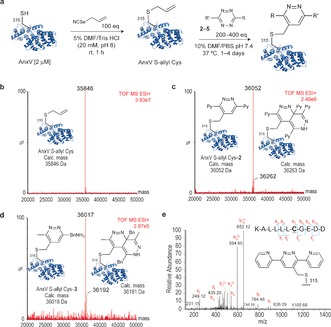
Chemical approach used for protein labeling through IEDDA reaction between S‐allyl Cys‐tagged proteins and tetrazines. a) The unstrained alkene handle was site‐specifically installed into AnxV by allylation of Cys315 through a [2,3]‐sigmatropic rearrangement with allyl selenocyanate. A tetrazine probe could selectively react with the installed alkene. b) Deconvoluted ESI‐MS spectrum of AnxV S‐allyl Cys. c) Deconvoluted ESI‐MS spectrum of the product of the reaction between AnxV S‐allyl Cys and PyTz **2**. d) Deconvoluted ESI‐MS spectrum of the product of the reaction between AnxV S‐allyl Cys and BnNH_2_‐Tz **3**. e) MS/MS spectrum of the *m*/*z* 652.12 doubly charged ion of the Cys‐modified peptide KALLLLCGEDD from AnxV. The underscore relates to the modified amino acid.

LC‐ESI‐MS analysis of the reactions with both tetrazine cores revealed that, besides the desired product, there was also the formation of a secondary product (10–20 %) corresponding to the addition of two tetrazine moieties to the proteins (identical results were obtained for both AnxV and C2Am; Figure 2 b,c and Supporting Information). Cross‐reactivity of reagents with other amino acids is a major issue for the efficiency of bioorthogonal labeling. For instance, it was recently reported that 2,5‐diaryltetrazoles, which were thought to selectively react with alkenes through light‐induced 1,3‐dipolar cycloadditions,^18^ also react with native tryptophan (Trp) residues on proteins.^19^ To verify whether the observed addition of two tetrazine moieties was the result of cross‐reactivity with other unsaturated canonical amino acids, tetrazine **2** was reacted with native proteins AnxV and C2Am (that is, without the S‐allyl handle) under the same IEDDA reaction conditions (200 equivalents of tetrazine, 37 °C for 96 h). These control experiments revealed that there is no addition of **2** to either AnxV or C2Am when the S‐allyl handle is not present (Supporting Information). Importantly, we showed that Trp and histidine (His), both possessing potentially reactive double bonds, do not react with **2** even under forcing conditions (Supporting Information). Moreover, the specificity of the IEDDA reaction towards the S‐allyl handle was further demonstrated by replacing it by dimethylallyl or propargyl groups that also gave no addition reaction in the presence of a tetrazine (Supporting Information). Finally, the Boc‐ and methyl ester‐protected S‐allyl Cys amino acid model was reacted with Py‐Tz **2** yielding the corresponding reduced and oxidized dihydropyridazine and pyridazine species, as well as a third compound resulting from the addition of a second tetrazine moiety to one of the pyridazine species (Supporting Information). These data on small molecules are consistent with our experiments on the protein context, which demonstrate that the formation of the bis‐adduct is not a result of cross reactivity with canonical amino acids but instead is the product of the reaction of a second tetrazine moiety with the first one. A detailed computational study and discussion of the whole reaction profile at the PCM(H_2_O)/M06‐2X/6‐31G(d) level of theory using complete models for both the amino acid and tetrazine counterparts is available in the Supporting Information (Figures S40–42).

To confirm the site of the modification, the protein conjugates were subjected to tryptic digestion, and the resulting peptide fragments were analyzed by LC‐MS/MS. This showed that modification with Py‐Tz **2** occurs at Cys315 where the S‐allyl handle has been chemically installed (Figure 2 d; results for C2Am are shown in the Supporting Information). Labeling of AnxV S‐allyl Cys with the tetrazine fluorophores Tz‐Rhod **4** and Tz‐Cy3 **5** was also studied. The reactions were set up in PBS buffer at pH 7.6 with 10 % DMF in the presence of 200 equivalents of tetrazine dyes **4** and **5**. The labeling of AnxV was confirmed by SDS‐PAGE (Figure 3 b) and LC‐ESI‐MS (Figure 3 c) analysis. Fluorescence imaging of the gel showed a band corresponding to the fluorescently labeled protein (Figure 3 b), while LC‐ESI‐MS indicated 40 % conversion to the product of the reaction with **5** after 12 h at 37 °C. For LC‐ESI‐MS analysis of the reaction with **4**, see the Supporting Information.

**Figure 3:**
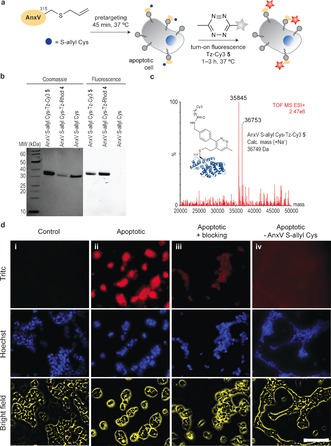
a) Pre‐targeting of apoptotic cells with AnxV S‐allyl Cys followed by IEDDA labeling with fluorogenic Tz‐Cy3 **5**. b) SDS‐PAGE of AnxV labeled with Tz‐Rhod **4** and Tz‐Cy3 **5** for 12 h at 37 °C. The labeled protein was purified by dialysis. Left image shows Coomassie blue‐stained gels and the right image presents the fluorescent imaging of the same gels before staining with Coomassie blue. c) ESI‐MS spectrum of the product of the reaction of AnxV S‐allyl Cys with Tz‐Cy3 **5**. d) Specific labeling of apoptotic cells (ii) pre‐targeted with AnxV S‐allyl Cys and subsequently labeled with Tz‐Cy3 **5**. Cells were imaged 3 h after addition of the fluorogenic dye **5**. Non‐apoptotic cells (i) were included as a control. The specificity of the labeling was confirmed through blocking of cells with non‐labeled AnxV (iii) prior to incubation with AnxV S‐allyl Cys or by treating cells only with the tetrazine probe Tz‐Cy3 **5** (iv) without preincubation with the targeting protein AnxV S‐allyl Cys. Apoptotic cells are shown red, while the nuclei counterstained with Hoechst 33 342 are shown blue. Scale bar=100 μm.

Having successfully demonstrated the tetrazine reaction with unstrained alkenes chemically installed on specific Cys residues in recombinant proteins in vitro, we then proceeded to test our strategy for live imaging using a cell model of apoptosis. Because we noticed some fluorescence background when using Tz‐Rhod **4** (Figure S36), we decided to use Tz‐Cy3 **5** dye in these studies. Dye **5** has greater aqueous solubility and a very low fluorescence background. Remarkably, with this tetrazine the S‐allyl Cys‐tagged AnxV protein was efficiently labeled, enabling visualization of apoptotic cells in a pre‐targeting approach (Figure 3 a). In particular, apoptotic HEK293 cells induced with actinomycin D were incubated with AnxV S‐allyl Cys for 45 min at 37 °C. After this preincubation step, the growth media was replaced to remove unbound protein and then treated with Tz‐Cy3 **5** for 3 h. After washing, the cells were imaged using both Tritc and Hoechst fluorescence channels (Figure 3 d). Significant labeling was observed (Figure 3 d ii) compared to non‐apoptotic cells (Figure 3 d i). Cells previously blocked with non‐labelled AnxV to block the externalized membrane lipid phosphatidylserine (PS) on the cell surface of apoptotic cells showed no labeling after exposure to AnxV S‐allyl Cys and subsequent reaction with Tz‐Cy3 **5** (Figure 3 d iii). A final control was performed where cells were treated only with tetrazine probe Tz‐Cy3 **5** without preincubation with the targeting protein AnxV S‐allyl Cys. Once again, apoptotic cells did not display any fluorescence (Figure 3 d iv). We also found that after 2 h apoptotic cells already display strong fluorescence (Figure S37), however, longer reaction times (3 h) results in higher contrast. Overall, these studies demonstrate the specificity of the tetrazine imaging probe for S‐allyl Cys AnxV in the presence of live cells and growth media. Additionally, our data show that protein‐specific activity is retained after the bioorthogonal IEDDA labeling in cells.

In summary, we demonstrate that unstrained S‐allyl handles precisely installed at predefined Cys residues within the sequence of a protein are suitable chemical handles for IEDDA reactions with tetrazine dyes. This strategy allowed for selective labeling of proteins in live cells using a pre‐targeting approach. The easy site‐specific installation and the small size of the allyl handle, which is potentially less disruptive compared to non‐canonical amino acids bearing bulky strained alkenes, is likely to be of a general benefit for other sensitive protein systems including antibodies used in pre‐targeting approaches. As such, we believe that the simple site‐specific labeling strategy disclosed here, which enables bioorthogonal live‐cell imaging, will find significant use in the biological community, allowing imaging of specific targets with minimal effects on their intrinsic properties.^20^

## Experimental Section

Synthetic procedures and kinetic studies are described in detail in the Supporting Information. Protein labeling through S‐allyl:tetrazine cycloaddition: S‐allyl handle was installed on AnxV by reaction with allyl selenocyanate (100 equivalents) for 1 h at room temperature. Labeling of AnxV S‐allyl Cys with the tetrazine fluorophores Tz‐Rhod **4** and Tz‐Cy3 **5** (5–12 h at 37 °C) was assessed by SDS‐PAGE and LC‐ESI‐MS analysis as described in the Supporting Information. For cell labeling and imaging, HEK293 cells were grown on coverslips and treated with 2 μm of actinomycin D for 12 h at 37 °C. After induction of apoptosis, cells were incubated with AnxV S‐allyl Cys in fresh media for 45 min at 37 °C (0.62 μm). Cells were then washed with d‐PBS before incubating for 1, 2, and 3 h with the tetrazine fluorophores **4** or **5** (375 μm), which were diluted in fresh growth media. Blocking studies were performed by preincubating apoptotic cells for 30 min with a 10x excess of non‐fluorescent AnxV (6.30 μm) before incubation with AnxV S‐allyl Cys. To test binding specificity (control), cells were treated with Tz‐Cy3 **5** without preincubation with AnxV S‐allyl Cys. After labeling, cells were washed 2x with PBS, fixed with PBS containing 4 % (w/v) formaldehyde, and finally imaged by fluorescence microscopy.

## Supporting information

As a service to our authors and readers, this journal provides supporting information supplied by the authors. Such materials are peer reviewed and may be re‐organized for online delivery, but are not copy‐edited or typeset. Technical support issues arising from supporting information (other than missing files) should be addressed to the authors.

Supplementary
