# Supplementary material for: A Minimal, Unstrained S‐Allyl Handle for Pre‐Targeting Diels–Alder Bioorthogonal Labeling in Live Cells
Source: Angew Chem Int Ed Engl. 2016 Oct 20;55(47):14683–7. doi: 10.1002/anie.201608438 (PMC5132151; doi:10.1002/anie.201608438)
Supplement: Supplementary file 1 — Supplementary [file ANIE-55-14683-s001.pdf]

Angewandte Chemie
Eine Zeitschrift der Gesellschaft Deutscher Chemiker

Supporting Information

A Minimal, Unstrained S-Allyl Handle for Pre-Targeting Diels–Alder Bioorthogonal Labeling in Live Cells

*Bruno L. Oliveira[+], Zijian Guo[+], Omar Boutureira[+], Ana Guerreiro, Gonzalo Jiménez-Osés, and Gonçalo J. L. Bernardes**

anie_201608438_sm_miscellaneous_information.pdf

Table of Contents

1. General Methods	S2
2. Experimental Section	S5
2.1. Synthetic procedures and characterization	S5
2.2. Kinetic measurements	S12
2.3. Fluorogenicity of the tetrazine probes	S17
2.4. Annexin-V protein modification analysis by LC–MS	S18
2.5. C2Am Protein modification analysis by LC–MS	S40
2.6. LC-MS/MS analysis	S47
2.7. NuPAGE Bis-Tris gels	S49
2.8. Cell growth conditions and fluorescent microscopy	S50
2.9. Toxicity and stability of **1**	S53
2.10. Computational details	S56
3. Spectroscopic data	S69
4. References	S78

1. General Methods

1.1. Chemical Synthesis

All reagents were obtained from commercial sources and used without further purification. The tetrazine compounds 3,6-di-2-pyridyl-1,2,4,5-tetrazine, 4-(6-methyl-1,2,4,5-tetrazin-3-yl)phenyl)methanamine and 6-methyl-tetrazine-sulfo-Cy3, shortened Py-Tz **2**, Bn-NH_2-Tz **3** and Tz-Cy3 **5** were obtained from Sigma-Aldrich, SiChem and Jena Bioscience, respectively. All reactions were performed using oven-dried glassware (200 °C) under an atmosphere of argon unless otherwise stated. Solvents were freshly distilled over sodium benzophenone ketyl (Et_2O) or calcium hydride (ACN, CH_2Cl_2, hexane and EtOAc). Distilled water was used for chemical reactions and Milli–QR purified water for protein manipulations. Thin layer chromatography was carried out using commercial Merck silica gel 60 F254 coated glass or aluminum plates. Visualization of the silica plates was achieved using a UV lamp (λ_{max} = 254 nm) and/or ninhydrin stain as appropriate. Flash column chromatography was performed using high-purity grade silica gel (Merck grade 9385) with a pore size 60 Å and 230 – 400 mesh particle size under air pressure. Proton (^{1}H NMR) and carbon (^{13}C NMR) nuclear magnetic resonance spectra were recorded on a 400 MHz DPX-400 Dual Spectrometer and Bruker 500 MHz AVIII HD Smart as indicated. Spectra were fully assigned using COSY, HSQC, HMBC and NOESY. Chemical shifts were reported in parts per million (ppm) using the residual solvent as internal standard. Coupling constants (*J*) are reported in Hz with the following splitting abbreviations: s = singlet, d = doublet, t = triplet, q = quartet, and quin = quintet. High-resolution mass spectra (HRMS) were recorded on a Waters ZQ LC-MS using electrospray ionization (ESI) or on a Waters LCT Premier spectrometer. Nominal and exact *m/z* values are reported in Daltons.

1.2. Protein conjugation

Liquid chromatography-mass spectrometry (LC–MS): Analysis were performed on a Xevo G2-S TOF mass spectrometer coupled to an Acquity UPLC system using an Acquity UPLC BEH300 C4 column (1.7 µm, 2.1 × 50 mm). Water with 0.1% formic acid (solvent A) and 70% ACN/30% water with 0.075% formic acid (solvent B), were used as the mobile phase at a flow rate of 0.2 mL min^{-1}. The gradient was programmed as follows: from 28% to 71% of B in 15 min, then to 100% of B in 5 minutes followed by a 1minute gradient to 60% of B and hold at this % for additional 5 minutes. The electrospray source was operated with a capillary voltage of 3.0 kV and a cone voltage of 40 V. Nitrogen was used as the desolvation gas at a total flow of 800 L h^{-1}. Total mass spectra were reconstructed from the ion series using the MaxEnt algorithm preinstalled on MassLynx software (v. 4.1 from Waters). To obtain the ion series described, the major peak(s) of the chromatogram were selected for integration and further analysis.

Protein LC–MS/MS: Experiments were performed using a nanoAcquity UPLC (Waters Corp., Milford, MA) system and an LTQ Orbitrap Velos hybrid ion trap mass spectrometer (Thermo Scientific, Waltham, MA). Protein samples were enzymatically digested by trypsin overnight. Separation of peptides was performed by reverse-phase chromatography using a Waters reverse-phase nano column (BEH C18, 75 µm i.d. x 250 mm, 1.7 µm particle size) at flow rate of 0.3 µL min^{-1}. Peptides were initially loaded onto a pre-column (Waters UPLC Trap Symmetry C18, 180 µm i.d. x 20mm, 5 µm particle size) from the nanoAcquity sample manager with 0.1% formic acid for 3 min at a flow rate of 10 µL min^{-1}. After this period, the column valve was switched to allow the elution of peptides from the pre-column onto the analytical column. Solvent A was water with 0.1% formic acid and solvent B was ACN with 0.1% formic acid. The linear gradient employed was 5 – 40% B in 60 min. The LC eluent was sprayed into the mass spectrometer by means of a New Objective nanospray source. All *m/z* values of eluting ions were measured in the Orbitrap Velos mass analyser, set at a resolution of 30000. Data dependent scans (top 20) were employed to automatically isolate and generate fragment ions by collision-induced dissociation in the linear ion trap, resulting in the generation of MS/MS spectra. Ions with charge states of 2+ and above were selected for fragmentation. Post-run, the data was processed using Protein Discoverer (version 1.3.,

ThermoFisher Scientific). Briefly, all MS/MS data were converted to mgf files and these were submitted to the Mascot search algorithm (Matrix Science, London UK) and searched against a custom database containing the corresponding protein and applying variable modifications of oxidation (M), deamination (NQ) and a custom modification (C), using a peptide tolerance of 25 ppm (MS) and 0.8 Da (MS/MS). Peptide identifications were accepted if they could be established at greater than 95.0% probability.

2. Experimental Section

2.1 Synthetic procedures and characterization

2.1.1. List of abbreviations used in the synthetic procedures

ACN = acetonitrile, calcd = calculated, DCM = dichloromethane, DIPEA = N,N-diisopropylethylamine, DMF= dimethylformamide, DMSO = dimethyl sulfoxide, Et_2O = Diethyl ether, ESI = electrospray ionization, eq = equivalents, EtOAc = ethyl acetate, HATU = 1-[Bis(dimethylamino)methylene]-1H-1,2,3-triazolo[4,5-b]pyridinium 3-oxid hexafluorophosphate, N-[(Dimethylamino)-1H-1,2,3-triazolo-[4,5-b]pyridin-1-ylmethylene]-N-methylmethanaminium hexafluorophosphate N-oxide, h = hours, HRMS = high-resolution mass spectra, NMR = nuclear magnetic resonance, MeOH = methanol, min = minutes, PBS = phosphate buffer saline, rt = room temperature (18 – 21 °C), TLC = thin-layer chromatography, s = seconds, THF = tetrahydrofuran.

2.1.2. Synthesis of N-Boc-S-allyl cysteine methyl ester (BocCysOMe-S-allyl)

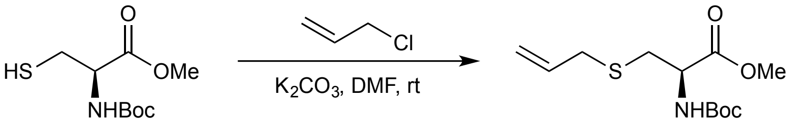

BocCysOMe (1 g, 0.004 mol) was added to a 25 mL round bottom flask and flashed with argon. DMF (5 mL) and K_2CO_3 (3.5 eq, 1.47 g, 0.014 mol) were added and the solution was cooled down to 0 ^{o}C in ice. Allyl chloride (5 eq, 1.50 g, 0.019 mol) was added dropwise. Then the reaction was allowed to stir at room temperature overnight. Reaction was diluted with 25 mL Et_2O and H_2O. Organic layer was separated and washed with H_2O (2 x 15 mL) and brine (2 x 15mL) subsequently, dried by $MgSO_4$. Solvents were removed and the residue was purified by column chromatography (1:3 EtOAc/petroleum ether) to afford BocCysOMe-S-allyl (0.90 g, 0.003 mol, 75% yield) as colorless oil which solidifies on storing at -20 °C. ^{1}H NMR (400 MHz, $CDCl_3$) δ 5.83 – 5.65 (m, $\underline{H}C=CH_2$, 1H), 5.38 – 5.24 (m, NH, 1H), 5.13 (q, J = 1.0 Hz, HC=C$\underline{H}$H, 1H), 5.12 – 5.08 (m, HC=CH$\underline{H}$, 1H), 4.59 – 4.44 (m, αH, 1H), 3.75 (s, OCH3, 3H), 3.19 – 3.04 (m, $S\underline{H_2}CH=CH_2$, 2H), 2.97 – 2.78 (m, $C\underline{H_2}$SAllyl, 2H), 1.44 (s, Boc, 9H). ^{13}C NMR (101 MHz, $CDCl_3$) δ 171.7 ($\underline{C}OOCH_3$), 155.2 (Boc, $\underline{C}OOC(CH_3)_3$), 133.8 (H$\underline{C}$=CHH), 118.0

(HC=CHH), 80.2 (Boc, COOC(CH_3)$_3$), 53.3 (αC), 52.7 (COOCH_3), 35.3 (SCH_2CH=CH_2), 33.0 (SCH_2Cα), 28.4 (Boc COOC(CH_3)$_3$). HRMS ESI$^+$: *m*/*z* calcd for $C_{12}H_{22}NO_4S$: 276.1270 [M+H]$^+$; found 276.1272.

2.1.3. Synthesis of S-allyl cysteine (S-allyl Cys 1)

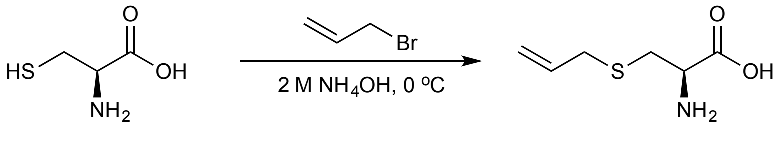

1

L-cysteine (1 g, 0.008 mol) was dissolved in 24 mL of NH_4OH 2M, and cooled in ice. Allyl bromide (1.5 eq, 1.5 g, 0.012 mol) was added dropwise and the reaction was allowed to stir vigorously for 1 h. The mixture was filtrated and the filtrate washed with ethanol three times providing **2** as a white solid (0.8 g, 0.005 mol, 62% yield). ^{1}H NMR (400 MHz, D_2O) δ 5.85 – 5.64 (m, CH_2=CH, 1H), 5.24 – 5.04 (m, CH_2=CH, 2H), 3.81 (dd, J = 7.5, 4.3 Hz, CHNH_2, 1H), 3.12 (dq, J = 7.3, 1.2 Hz, SCH_2CH=CH_2, 2H), 3.04 – 2.78 (m, SCH_2Cα, 2H). ^{13}C NMR (101 MHz, D_2O) δ 172.8 (COOH), 133.4 (CH_2=CH), 127.1, 124.3, 118.3 (CH_2=CH), 53.4 (αC), 33.8 (SCH_2CH=CH_2), 30.6 (SCH_2Cα). HRMS ESI$^+$: *m*/*z* calcd for $C_6H_{12}NO_2S$: 162.0589 [M+H]$^+$; found 162.0588.

2.1.4. Synthesis of dihydropyridazine (Cys-Py-Tz$_{red}$) and pyridazine (Cys-Py-Tz$_{oxi}$) derivatives of BocCysOMe-S-allyl.

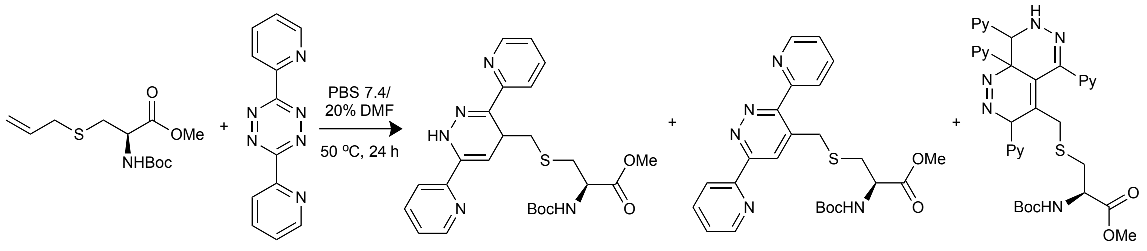

Py-Tz **2** | **Cys-Py-Tz-red** | **Cys-Py-Tz-oxi** | **bis-addition product**

BocCysOMe-S-allyl (50 mg, 0.181 mmol) and 3,6-Di(2-pyridinyl)-1,2,4,5-tetrazine (Py-Tz **2**, 3 eq, 128 mg, 0.541 mmol) were dissolved in PBS pH 7.6/ 20% DMF (2 mL) and heated for 24 h at 50 ºC. After complete removal of the solvents the residue was purified by column chromatography (DCM to DCM/MeOH 5:1) to afford a mixture of compounds **Cys-Py-Tz$_{red}$** (19 mg, 0.039, 22% yield), **Cys-Py-Tz$_{oxi}$** (20 mg, 0.041 mmol, 23% yield) and **bis-addition product** (19 mg, 0.027 mmol, 15% yield). Characterization

for **Cys-Py-Tz$_{red}$**: ^{1}H NMR (500 MHz, $CDCl_3$) δ 9.43 – 9.35 (m, NH, 1H), 8.65 – 8.49 (m, pyridyl, 2H), 8.08 (ddt, J = 8.1, 2.3, 1.1 Hz, pyridyl, 1H), 7.73 – 7.70 (m, pyridyl, 2H), 7.70 – 7.64 (m, pyridyl, 1H), 7.25 – 7.17 (m, pyridyl, 2H), 5.72 (td, J = 6.3, 2.4 Hz, pyridazine, C$\underline{H}$-CHCH_2S, 1H), 5.61 (t, J = 10.8 Hz, BOC NH, 1H), 4.62 – 4.54 (m, Hα, 1H), 4.51 (ddt, J = 8.8, 6.2, 4.3 Hz, pyridazine, C$\underline{H}$$CH_2SCH_2$Cα, 1H), 3.77 – 3.64 (s, COOC$\underline{H_3}$, 3H), 3.20 – 2.96 (m, SC$\underline{H_2}$Cα, 2H), 2.84 (ddd, J = 20.2, 13.0, 4.3 Hz, C$\underline{H}$HSCH_2Cα, 1H), 2.59 (dd, J = 13.0, 9.7 Hz, CH$\underline{H}$SCH_2Cα,1H), 1.39 (s, Boc, 9H). ^{13}C NMR (126 MHz, $CDCl_3$) δ 171.9 ($COOCH_3$), 155.4 (pyridyl), 155.3 (Boc $\underline{C}$OOC$(CH_3)_3$), 154.6 (pyridyl), 150.3 (pyridyl), 148.8 (pyridyl), 148.5 (pyridyl), 141.6 (pyridyl), 136.8 (pyridyl), 136.2 (pyridyl), 123.2 (pyridazine), 122.9 (pyridazine), 120.7 (pyridyl), 119.3 (pyridyl), 97.0 (pyridazine, $\underline{C}$H-CHCH_2S), 80.1 (Boc COO$\underline{C}$$(CH_3)_3$), 53.4 (αC), 52.5 (COO$\underline{C}$$H_3$), 35.7 ($\underline{C}$$H_2SCH_2$Cα), 34.3 (S$\underline{C}$$H_2$Cα), 31.7 (pyridazine, $\underline{C}$HCH_2SCH_2Cα), 28.4 (BOC COOC($\underline{C}$$H_3$)$_3$). HRMS ESI$^+$: *m*/*z* calcd for $C_{24}H_{29}O_4N_5NaS$: 506.1832 [M+Na]$^+$, found 506.1823. Characterization for **Cys-Py-Tz$_{oxi}$**: ^{1}H NMR (400 MHz, $CDCl_3$) δ 8.78 – 8.67 (m, 1H pyridazine + 2H pyridyl, 3H), 8.65 (s, pyridazine, 1H), 8.29 (d, J = 7.9, 1.0 Hz, pyridyl, 1H), 7.97 – 7.82 (m, pyridyl, 2H), 7.47 – 7.35 (m, pyridyl, 2H), 5.44 (d, J = 8.1 Hz, NH, 1H), 4.58 – 4.43 (m, Hα, 1H), 4.39 (s, C$\underline{H_2}$SCH_2Cα, 2H), 3.67 (s, COOC$\underline{H_3}$, 3H), 2.96 – 2.76 (m, SC$\underline{H_2}$Cα, 2H), 1.40 (s, Boc, 9H). ^{13}C NMR (101 MHz, $CDCl_3$) δ 171.4 ($\underline{C}$OOCH_3), 157.7 (pyridyl), 157.5 (pyridyl), 155.8 (pyridyl), 155.1 (Boc $\underline{C}$OOC$(CH_3)_3$), 153.2 (pyridyl), 149.5 (pyridazine), 148.5 (pyridazine), 138.8 (pyridyl), 137.2 (pyridyl), 137.1 (pyridyl), 125.9 (pyridazine), 124.8 (pyridazine), 123.9 (pyridyl), 121.8 (pyridyl), 80.2 (Boc COO$\underline{C}$$(CH_3)_3$), 53.3 (αC), 52.6 (COO$\underline{C}$$H_3$), 34.4 (S$\underline{C}$$H_2$Cα), 33.7 (S$\underline{C}$$H_2$), 28.3 (BOC COOC($\underline{C}$$H_3$)$_3$). HRMS ESI$^+$: *m*/*z* calcd for $C_{24}H_{27}O_4N_5NaS$: 504.1676 [M+H]$^+$; found 504.1673. Characterization for **bis-addition product**: ^{1}H NMR (500 MHz, $CDCl_3$) δ 10.14 (s, 1H), 8.86 (s, 1H), 8.65 (ddd, J = 4.8, 1.9, 1.0 Hz, 1H), 8.58 (ddd, J = 4.8, 1.9, 0.9 Hz, 1H), 8.43 (ddd, J = 4.9, 1.8, 0.9 Hz, 1H), 8.36 (ddd, J = 4.8, 1.8, 0.9 Hz, 1H), 8.10 (d, J = 8.0 Hz, 1H), 8.01 (dt, J = 8.2, 1.1 Hz, 1H), 7.92 – 7.82 (m, 3H), 7.68 – 7.56 (m, 2H), 7.53 (ddd, J = 8.2, 7.3, 1.8 Hz, 1H), 7.36 – 7.28 (m, 1H), 7.14 – 7.06 (m, 2H), 7.05 – 6.98 (m, 1H), 5.28 (d, J = 8.2 Hz, 1H), 5.00 (dd, J = 9.0, 6.5 Hz, 1H), 4.43 – 4.28 (m, 1H), 3.59 (s, 3H), 2.91 (dd, J = 13.2, 6.5 Hz, 1H), 2.80 – 2.49 (m, 3H), 1.39 (s, 9H). ^{13}C NMR (126 MHz, $CDCl_3$) δ 171.7, 164.6, 155.7, 155.3, 154.6, 151.5, 149.6, 149.5, 148.4, 147.4, 143.2, 137.3, 137.1, 136.6, 136.4, 135.9, 134.2, 125.0, 123.8, 122.8, 122.5,

122.1, 121.9, 121.3, 119.2, 103.7, 80.1, 61.2, 60.5, 53.6, 52.4, 34.6, 33.3, 32.9, 28.4. HRMS ESI$^+$: *m/z* calcd for $C_{36}H_{38}N_9O_4S$: 692.2767 $[M+H]^+$; found 692.2766.

2.1.5. Synthesis of the rhodamine precursor Rhod-NH$_2$

Rhod-NH$_2$ was prepared according to a published protocol.[1]

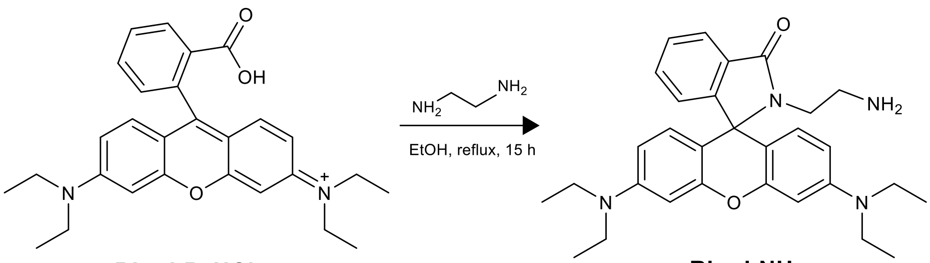

Rhodamine B (1.2 g, 2.5 mmol) was dissolved in ethanol (30 mL). Ethylenediamine (1.3 eq, 0.22 mL, 3.25 mmol) was added dropwise to the solution and refluxed overnight (15 h) until the solution loses its red color. The solvent was removed by evaporation. Water (20 mL) was added to the resultant and extracted with CH_2Cl_2 (20 mL × 2). The combined organic phase was washed twice with water and dried over Na_2SO_4. The solvent was removed by evaporation and dried in vacuo, affording a pale-pink solid of Rhod-NH$_2$ after purification by column chromatography (DCM to DCM/MeOH, 95:5) (0.908 g, 1.875 mmol, 75% yield). ^1H NMR (400 MHz, CD_3OD) δ 7.83 - 7.86 (m, 1H), 7.40 - 7.43 (m, 2H), 7.04 - 7.06 (m, 1H), 6.42 (s, 1H), 6.40 (s, 1H), 6.35 (d, J = 2.3 Hz, 2H), 6.25 (dd, J = 8.9, 2.6 Hz, 2H), 3.24 - 3.34 (m, 10H), 2.67 (t, J = 5.4 Hz, 2H), 1.14 (t, J = 7.2 Hz, 12H). ^{13}C NMR (101 MHz, CD_3OD) 169.6, 153.7, 153.4, 149.1, 133.1, 130.0, 128.3, 128.1, 123.7, 122.4, 108.1, 104.1, 97.6, 65.8, 43.9, 38.6, 37.8, 11.2. HRMS ESI$^+$: *m/z* calcd for $C_{30}H_{37}N_4O_2$ 485.2916 $[M+H]^+$; found 485.2900.

2.1.6. Synthesis of the tetrazine fluorophore Tz-Rhod 4

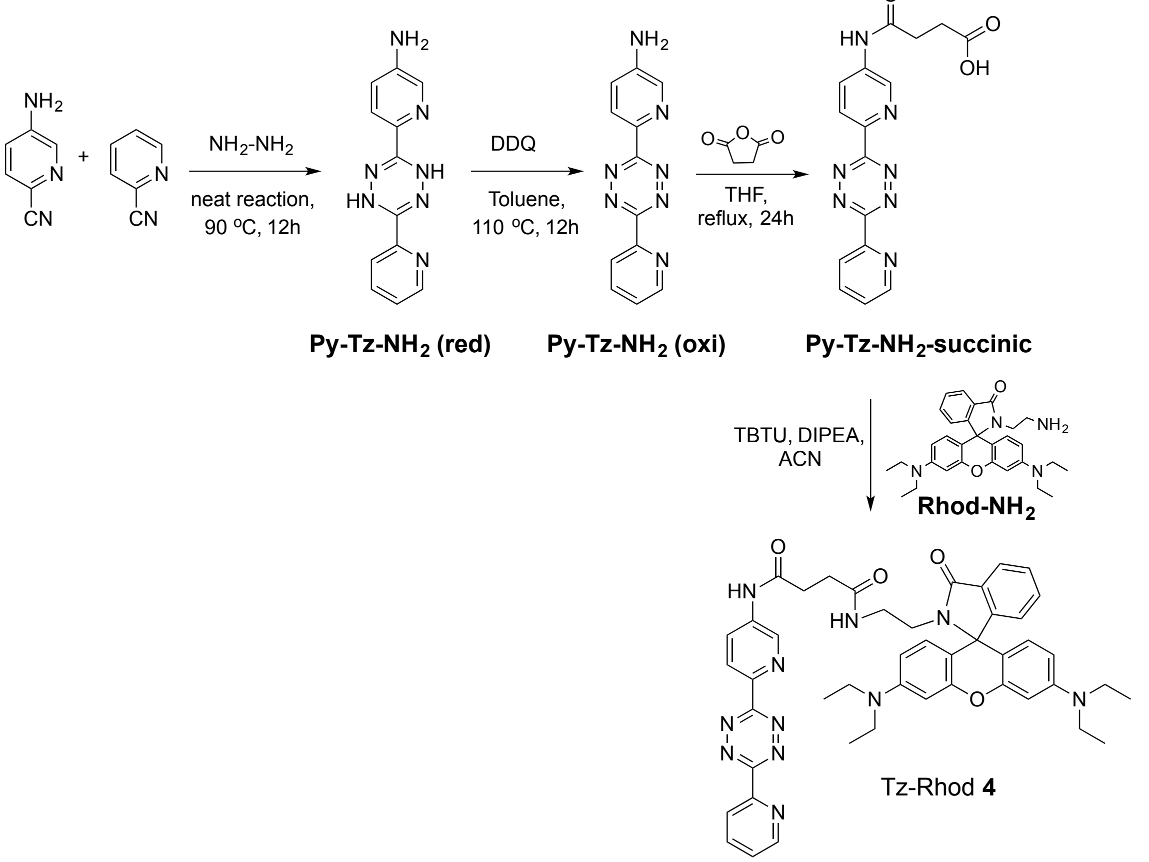

Synthesis of 6-(6-(Pyridin-2-yl)-1,2,4,5-tetrazin-3-yl)pyridin-3-amine (Py-Tz-NH_2 oxi)

Equimolar amounts of 5-amino-2-cyanopyridine (500 mg, 4.8 mmol) and 2-cyanopyridine (572 g, 4.8 mmol) were mixed with 64% aqueous hydrazine (0.932 ml, 19.2 mmol) and heated for 12 h to 90 ºC behind a blast shield. The mixture was allowed to cool to room temperature and the orange precipitate was isolated by filtration and washed with cold water and dried under vacuum. The crude orange solid of 3-(5-aminopyridin-2-yl)-6-(pyridin-2-yl)-1,4-dihydro-s-tetrazine (**Py-Tz-NH_2 red**, 1g) was dissolved in anhydrous toluene (25 mL) under nitrogen and 2,3-dichloro-5,6-dicyano-1,4-benzoquinone (DDQ) was added portionwise (1 g, 4.40 mmol). The reaction mixture was allowed to reflux and stir for 12 hr. The reaction mixture was concentrated in vacuo onto deactivated silica gel and chromatographed using a gradient of MeOH (0 - 10%) in DCM to give 356 mg (1.01 mmol, 22.9% yield) of a red solid. ^{1}H NMR (500 MHz, d_6-DMSO): δ 8.91 (d, J = 4.6 Hz, 1H), 8.57 (m, 1H), 8.37 (d, J = 8.7 Hz, 1H), 8.24 (d, J =

2.6 Hz, 1H), 8.13 (m, 1H), 7.67 (m, 1H), 7.14 (dd, J = 8.7, 2.7 Hz, 1H). ^{13}C NMR (126 MHz, d_6-DMSO): δ 163.0, 162.5, 150.9, 148.7, 147.9, 138.1, 137.6, 136.4, 126.7, 126.2, 124.2, 119.5. HRMS ESI$^+$: *m/z* calcd for $C_{12}H_{10}N_7$ $[M+H]^+$ 252.0997; found 252.0986.

Synthesis of 4-oxo-4-((6-(6-(pyridin-2-yl)-1,2,4,5-tetrazin-3-yl)pyridin-3-yl)amino)butanoic acid (Py-Tz-NH$_2$-succinic)

A mixture of **Py-Tz-NH$_2$ oxi** (70 mg, 0.279 mmol) and succinic anhydride (3.5 eq, 100 mg, 1.0 mmol) in THF (5 mL) was refluxed for 24 h in a sealed flask. After cooling, the precipitate was thoroughly washed with DCM (2 × 5 mL), ethyl ether (5 mL) and THF (5mL) to yield **Py-Tz-NH$_2$-succinic** as a purple solid (62 mg, 0.176 mmol, 63.3% yield). ^{1}H NMR (500 MHz, d_6-DMSO) δ 12.17 (br s, COOH, 1H), 10.68 (s, NH, 1H), 9.06 (d, J = 2.2 Hz, 1H), 8.94 (d, J = 4.2 Hz, 1H), 8.64 (d, J = 16.0 Hz, 1H), 8.59 (d, J = 8.3 Hz, 1H), 8.42 (d, J = 8.7, 2.4 Hz, 1H), 8.16 (td, J = 7.8, 1.6 Hz, 1H), 7.73 (dd, J = 6.7, 4.8 Hz, 1H), 2.68 (t, J = 6.8 Hz, 2H), 2.60 (t, J = 6.7 Hz, 2H). ^{13}C NMR (126 MHz, d_6-DMSO): δ 174.0, 172.1, 163.1, 162.5, 151.8, 150.4, 143.9, 141.8, 139.7, 137.7, 126.6, 126.0, 125.3, 124.1, 31.7, 29.3, 29.1. HRMS ESI$^+$: *m/z* calcd for $C_{16}H_{14}N_7O_3$ $[M+H]^+$ 352.1158; found 352.1192.

Synthesis of N1-(2-(3',6'-bis(diethylamino)-3-oxospiro[isoindoline-1,9'-xanthen]-2-yl)ethyl)-N4-(6-(6-(pyridin-2-yl)-1,2,4,5-tetrazin-3-yl)pyridin-3-yl)succinamide (Tz-Rhod 4)

N,N-Diisopropylethylamine (DIPEA, 3 eq, 22 mg, 0.170 mmol) was added to a stirred mixture of **Py-Tz-NH$_2$-succinic** (20 mg, 0.056 mmol), and HATU (1.5 eq, 32 mg, 0.085 mmol) in ACN (2 mL). After stirring for 16 h at room temperature, Rhod-NH$_2$ (2 eq, 55 mg, 0.113 mmol) was added and reacted for 1h. The mixture was evaporated and the product was purified by column chromatography on silica using a gradient of MeOH (0-10%) in DCM giving Tz-Rhod **4** as a dark purple solid (9.6 mg, 0.012 mmol, 21.4%). ^{1}H NMR (500 MHz, $CDCl_3$) δ = 10.32 (s, NH, 1H), 8.99 (m, NH, 1H), 8.95 (d, J = 2.3 Hz, 1H), 8.74 (d, J = 8.8 Hz, 1H), 8.69 (d, J = 8.7 Hz, 1H), 8.49 (dd, J = 8.7, 2.5 Hz, 1H), 8.01 (td, J = 7.8, 1.7 Hz, 1H), 7.91 (m, 1H), 7.58 (m, 1H), 7.49 (m, 2H), 7.17 (m, 1H), 7.12 (m, 1H), 6.47 (m, 2H), 6.40 (m, 2H), 6.30 (dd, J = 8.8, 2.5 Hz, 2H), 3.37 (m, 10H), 3.11 (m, 2H), 2.73 (m, 2H), 2.60 (m, 2H), 1.19 (m, 12). ^{13}C NMR (126 MHz, $CDCl_3$): δ

172.6, 171.7, 170.0, 163.5, 153.6, 153.3, 150.9, 150.2, 149.0, 144.0, 142.0, 138.4, 137.4, 132.9, 130.4, 128.4, 128.3, 126.6, 126.4, 125.1, 124.3, 124.0, 122.8, 108.3, 104.5, 97.8, 65.8, 44.3, 41.1, 40.0, 33.5, 31.3, 12.6. HRMS ESI$^+$: *m*/*z* calcd for $C_{46}H_{48}N_{11}O_4$ $[M+H]^+$ 818.3890; found 818.3891.

2.1.7. Control studies with amino acids with potentially reactive double bonds

On proteins Py-Tz **2** reacts with AnxV and C2Am affording a sub-prodruct (10 - 20%) corresponding to the conjugation of two tetrazines. To check if the double addition was a result of cross-reactivity with native amino acids present on the proteins, Py-Tz **2** was reacted with tryptophan and histidine. These amino acids have in their structure double bonds that are potentially reactive in Diels-Alder reactions.

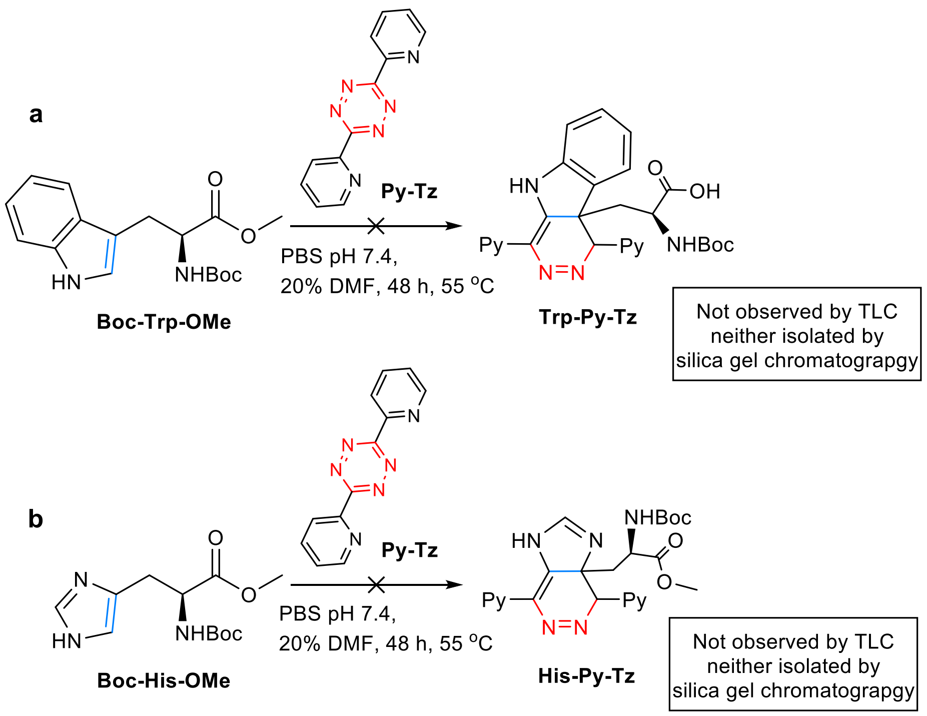

Boc-Trp-OMe (80 mg, 0.251 mmol) or **Boc-His-OMe** (30 mg, 0.111 mmol) and an excess of Py-Tz **2** (3 eq) were dissolved in 2 mL of PBS/ 20% DMF in a sealed tube. The reaction was allowed to stir at 55 °C for 48 h. TLC analysis showed no formation of **Trp-Py-Tz** or **His-Py-Tz**. The crude mixtures were purified by column chromatography (60% EtOAc in petrol), providing unreacted **Boc-Trp-OMe** and **Boc-His-OMe** with identical NMR spectroscopic data to the starting materials.

2.2. Kinetic measurements

The kinetics were set to be performed in pseudo first order with the dienophile being at least 375 times higher than the concentration of the diene. A stock solution of the compound Cys-allyl **1** was prepared in $CD_3OD:D_2O$ 1:1 and the exact concentration determined by qNMR, using potassium hydrogen phthalate as internal standard. Working solutions were prepared by dilution with PBS pH 7.6/MeOH 1:1. Solutions of the alkene **1** (15 - 50 mM) and the tetrazines **2** - **5** (0.04 mM) were mixed in quartz cuvettes and the reaction followed at 37 ºC using a UV/vis (Cary 100, Agilent) or a fluorescence spectrophotometer (Cary Eclipse, Agilent). For each diene and dienophile pair at least 2 - 3 replicates with 4 - 5 different concentrations were performed.

- Reaction between Cys S-allyl **1** and tetrazines Py-Tz **2** and Bn-NH_2-Tz **3**: These reactions were monitored through the decreasing of the absorption of the tetrazines at 320 nm. Pseudo-first-order rate constants (k_{obs}) were determined by fitting the data to a one-phase exponential decay using GraphPad Prism (GraphPad Software, La Jolla, CA, USA). Data were fit without value constraints. Second-order rate constants (k_2) were determined by plotting the pseudo-first-order rate constants against the corresponding alkene concentrations, followed by linear regression (**Figure S1** and **S2**).

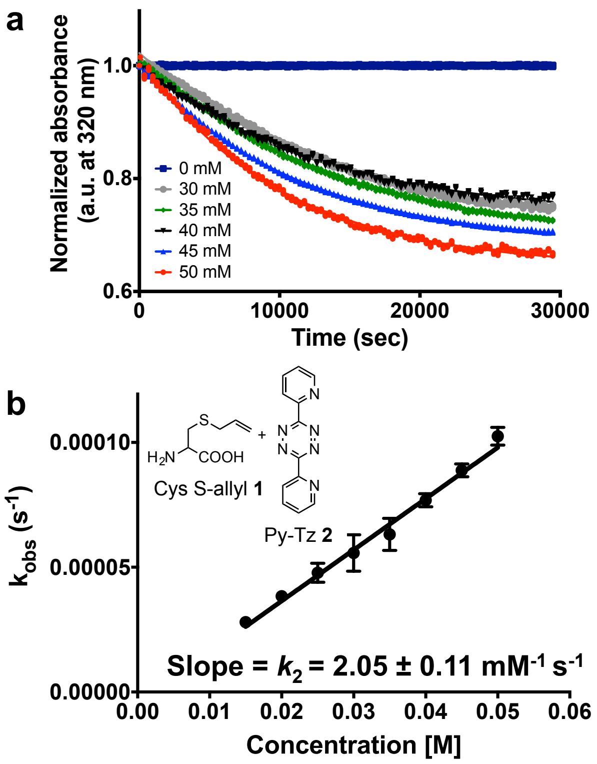

Figure S1 (**a**) Decrease of absorbance at 320 nm for Py-Tz **2** (0.04 mM) over time upon reaction with increasing concentrations of Cys S-allyl **1** in PBS/MeOH, pH 7.6 at 37 ºC. (**b**) Plot of k_{obs} vs the concentration of Cys S-allyl **1**. Each data point is the average of at least three independent runs (error bars lie within the symbols when not visible). The second order rate constant (k_2, 2.1 ± 0.1 × 10^{-3} M^{-1} s^{-1}) was calculated from the slope of the graph using the relationship k_{obs} = [Cys S-allyl](k_2).

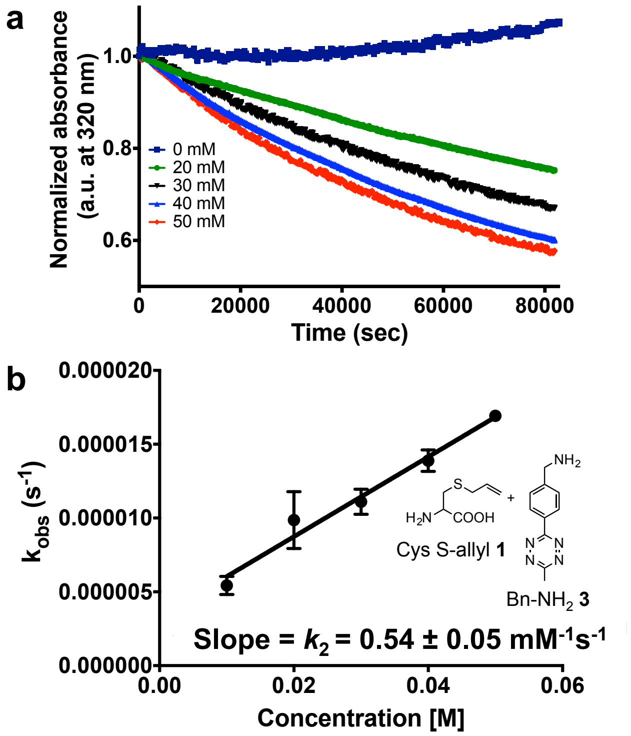

Figure S2 (**a**) Decrease of absorbance at 320 nm for Bn-NH_2-Tz **3** (0.04 mM) over time upon reaction with increasing concentrations of Cys S-allyl **1** in PBS/MeOH, pH 7.6 at 37 ºC. (**b**) Plot of k_{obs} vs the concentration of Cys S-allyl **1**. Each data point is the average of three runs (for 0.05 M error bar lie within the symbol). The second order rate constant (k_2, 0.5 ± 0.05 × 10^{-3} $M^{-1}s^{-1}$, n = 3) was calculated from the slope of the graph using the relationship k_{obs} = [Cys S-allyl](k_2).

- Reaction between Cys S-allyl **1** and tetrazines Tz-Rhod **4** and Tz-Cy3 **5**: in the case of the fluorogenic tetrazines the fluorescent increment data were fitted to a single exponential increase using GraphPad Prism. The resulted values for k_{obs} were plotted against alkene concentrations. Linear regression of this plot allowed deriving the second-order rate constant for the respective alkene–tetrazine couples (**Figure S3**).

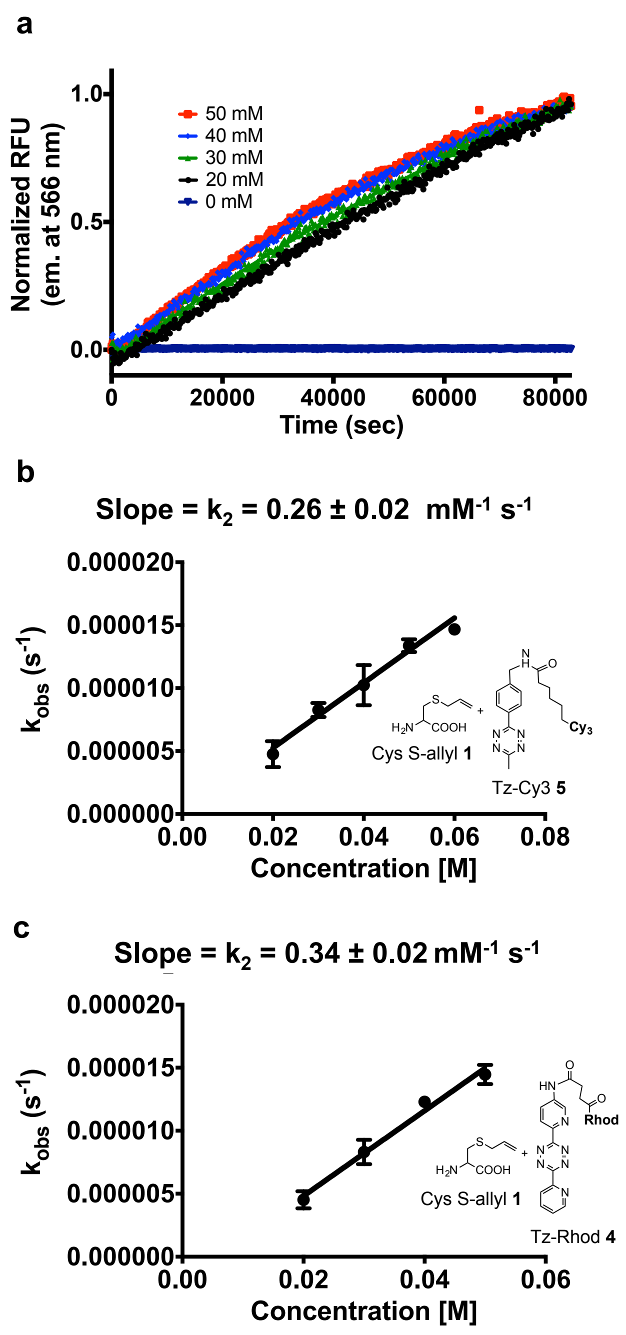

Figure S3 Determination of rate constants k_2 for the reaction of Tz-Rhod **4** and Tz-Cy3 **5** with Cys-allyl **1** The fluorescence increase was monitored at 570 nm (Tz-rhod **4**) and 566 nm (Tz-Cy3 **5**) with excitation at 553 nm. (**a**) Response of the fluorescence for Tz-Cy3 **5** upon addition of different concentrations of Cys-allyl **1**; by fitting the data to a single exponential increase equation, k_{obs} values were determined. (**b** and **c**) k_{obs} values for Tz-Rhod **4** and Tz-Cy3 **5** were plotted against the concentration of Cys-allyl **1** and the data subjected to a linear fit to yield the second-rate constant (k_2 for Tz-Rhod **4** =

$0.34 \pm 0.03 \times 10^{-3}$ M^{-1} s^{-1}, n = 2; k_2 for Tz-Cy3 **5** = $0.26 \pm 0.02 \times 10^{-3}$ M^{-1} s^{-1}, n = 3). Error bars lie within the symbols when not visible.

The second-rate constant for the reaction between 5-norbornene-2-methanol (**Norb**) and Tz-Cy3 **5** was also determined to be used as a reference (**Figure S4**).

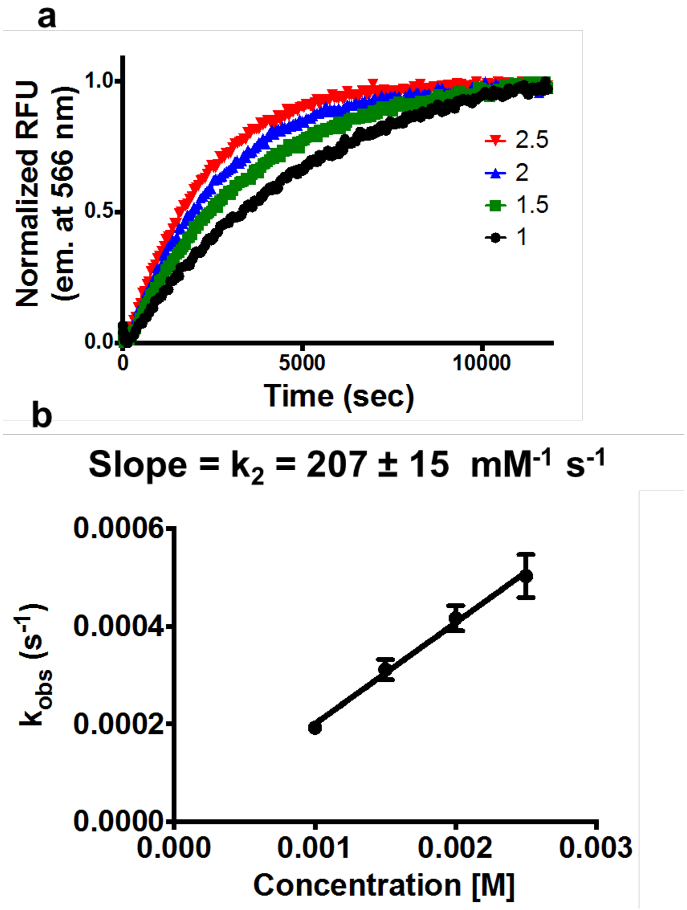

Figure S4 Determination of rate constant k_2 for the reaction of **Norb** with Tz-Cy3 **5**. (**a**) Response of the fluorescence for Tz-Cy3 **5** upon addition of different concentrations of **Norb** (1 mM to 2.5 mM). (**b**) Second-order rate constant (k_2, 0.21 ± 0.02 M^{-1} s^{-1}; n = 2) was obtained from linear regression analyses of k_{obs} versus [Norb] plot. Error bar for 0.001 M lie within the symbol. 80/20 endo-/exo-isoforms.

2.3. Fluorogenicity of the tetrazine probes

A 0.2 mM solution of the tetrazine-fluorophores Tz-Rhod **4** and Tz-Cy3 **5** in PBS buffer pH 7.6 was reacted with 1000 equivalents of **Norb** at 37 °C. Emission spectra were recorded before and after 2 h reaction (**Figure S5**).

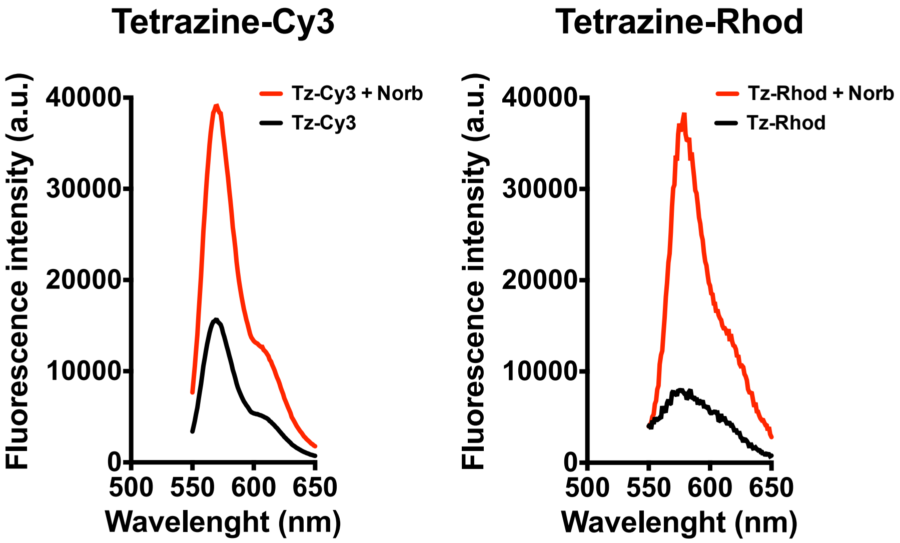

Figure S5 “Turn on” fluorescence of tetrazine-fluorophores upon reaction with norbornene. Excitation wavelengths: Tz-Rhod **4**, 550 ± 20 nm; Tz-Cy3 **5**, 540 ± 20 nm. Tz-Rhod **4** and Tz-Cy3 **5** display 5-fold and 2.5-fold fluorescence enhancement upon reaction.

2.4. Annexin-V protein modification analysis by LC-MS

2.4.1. Sequence of Annexin-V (AnxV, modified residue highlighted)[2]

AQVLRGTVTDFPGFDERADAETLRKAMKGLGTDEESILTLLTSRSNAQRQEISAA
FKTLFGRDLLDDLKSELTGKFEKLIVALMKPSRLYDAYELKHALKGAGTNEKVL
TEIIASRTPEELRAIKQVYEEEYGSSLEDDVVGDTSGYYQRMLVVLLQANRDPD
AGIDEAQVEQDAQALFQAGELKWGTDEEKFITIFGTRSVSHLRKVFDKYMTISGF
QIEETIDRETSGNLEQLLLAVVKSIRSIPAYLAETLYYAMKGAGTDDHTLIRVMV
SRSEIDLFNIRKEFRKNFATSLYSMIKGDTSGDYKKALLLL**C**GEDD

Calculated average isotopic mass = 35805.58 (*N*-terminal Met cleaved)

A typical analysis of a conjugation reaction by LC–MS is described below. The total ion chromatogram, combined ion series, and deconvoluted spectra are shown for AnxV. Identical analyses were carried out for all the conjugation reactions performed in this work.

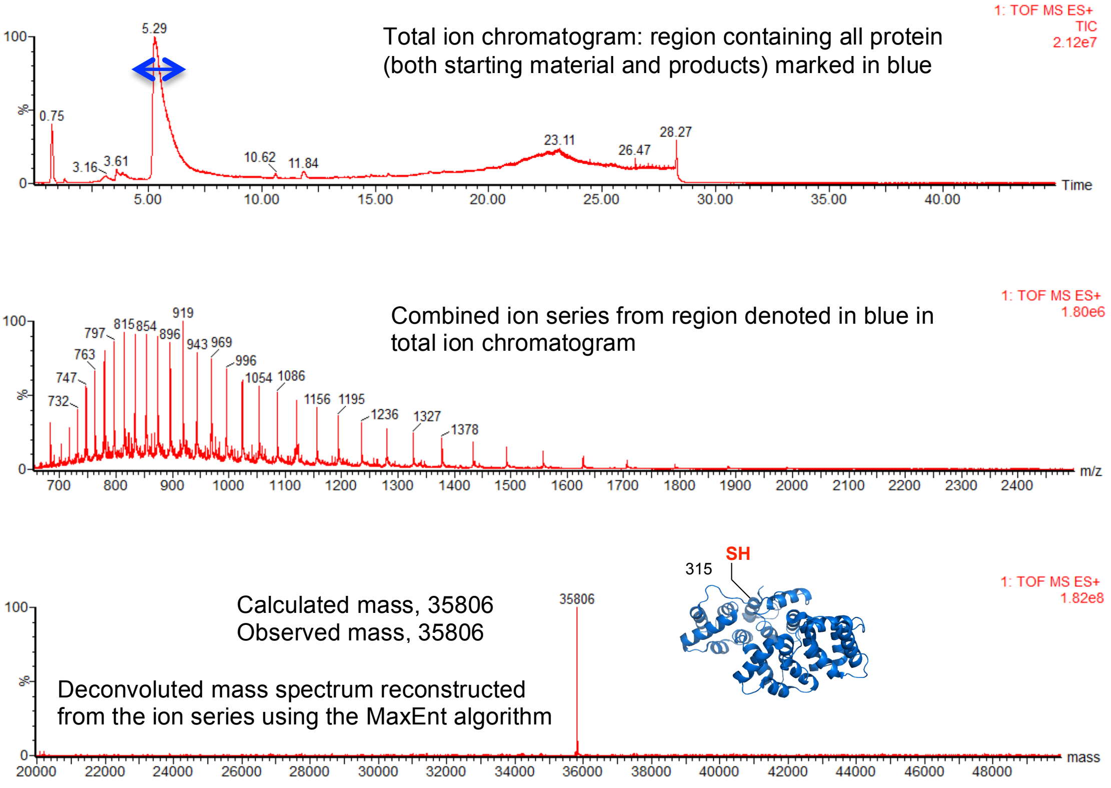

Figure S6 LC–MS analysis of AnxV.

Control: reaction of Ellman's reagent with AnxV

Control studies to proof quantitative modification of the free cysteines of the AnxV and C2Am were performed using the Ellman's test. As an example, the ESI-MS spectra for the reaction between AnxV and the Ellman's reagent are shown below.

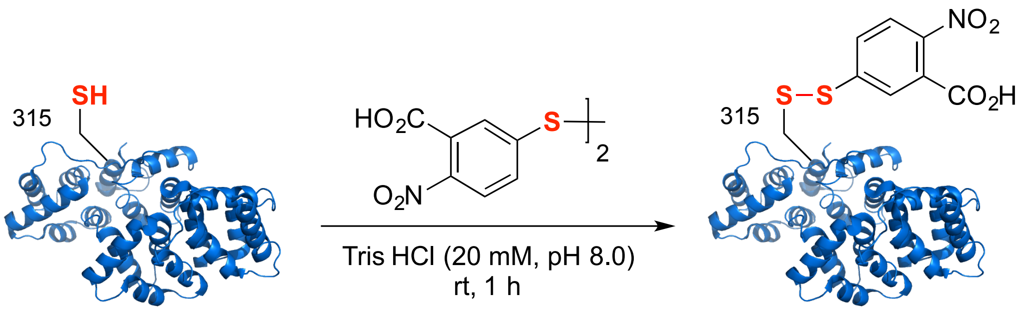

A 10 μL aliquot of AnxV (1 mg/mL, 0.616 nmol) in 20 mM Tris HCl buffer at pH 8.0 was transferred to a 0.5 mL eppendorf tube. Ellman's reagent (5,5-dithio-bis-(2-nitrobenzoic acid), 2 μL of a 14 mg/mL stock solution in H_2O, 70.7 nmol) was added and the resulting mixture vortexed for 30 seconds. After 1 h of additional shaking at room temperature, an aliquot was analyzed by LC–MS (3 μL diluted by 7 μL of 20 mM Tris HCl buffer at pH 8.0) and complete conversion to the expected Ellman's product (calculated mass, 36003; observed mass, 36003) was observed.

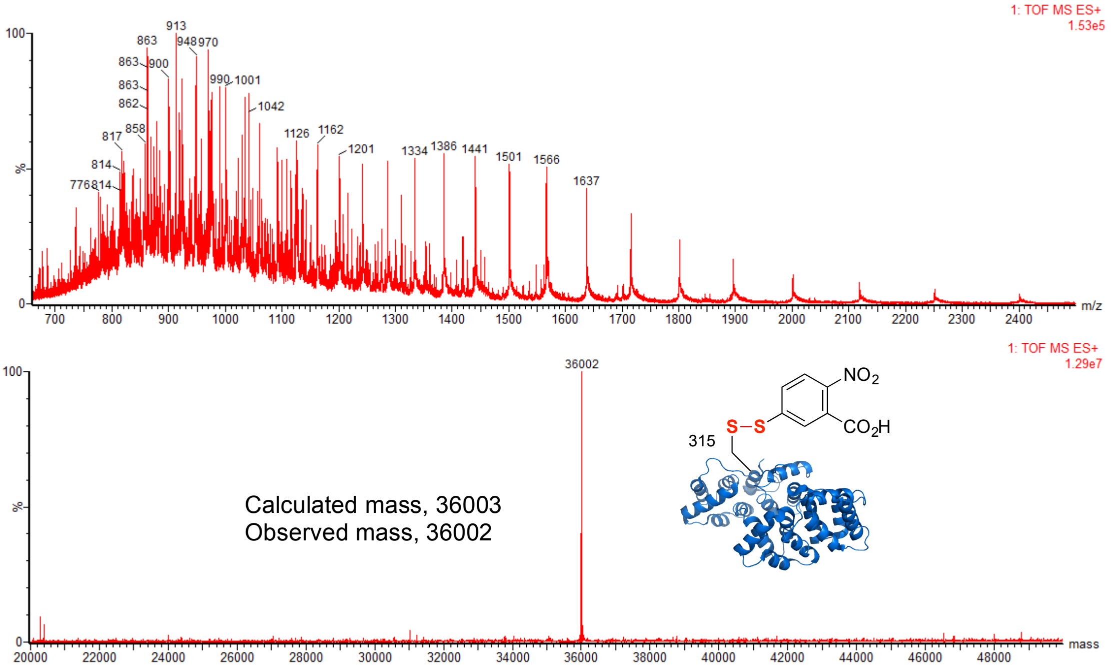

Figure S7 ESI–MS of the reaction of Ellman's reagent with AnxV.

2.4.2. Synthesis of Annexin-V S-allyl Cys (AnxV S-allyl Cys)

Direct alkylation with allyl chloride at pH 8.0

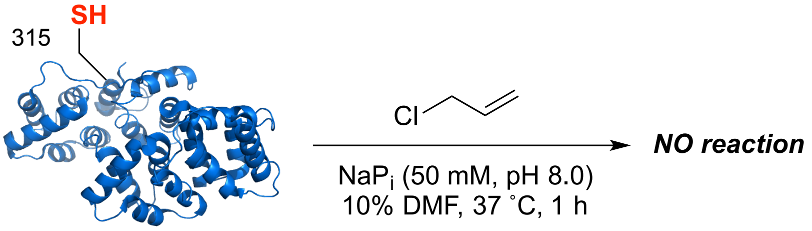

AnxV was prepared as a 0.25 mg/mL solution in 50 mM sodium phosphate buffer at pH 8.0 and 20 µL (0.140 nmol) were transferred to a 0.5 mL eppendorf tube. Allyl chloride (2.5 µL of a solution of 0.5 µL of allyl chloride in 218 µL DMF, 69.8 nmol) was added and the resulting mixture vortexed for 30 seconds. After 1 h of additional shaking at 37 ºC, a 3 µL aliquot diluted in 7 µL of 50 mM sodium phosphate buffer at pH 8.0 was analyzed by LC–MS and starting protein AnxV (calculated mass, 35806; observed mass, 35806) was detected unaltered.

Direct alkylation with allyl chloride at pH 11.0

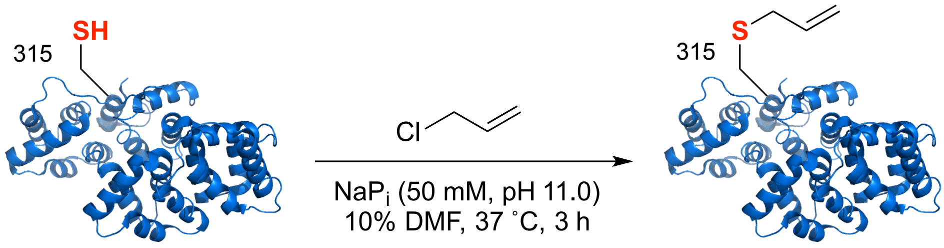

AnxV was prepared as a 0.25 mg/mL solution in 50 mM sodium phosphate buffer at pH 11.0 and 20 µL (0.140 nmol) were transferred to a 0.5 mL eppendorf tube. Allyl chloride (2.5 µL of a solution of 0.5 µL of allyl chloride in 218 µL DMF, 69.8 nmol) was added and the resulting mixture vortexed for 30 seconds. After 3 h of additional shaking at 37 ºC, a 3 µL aliquot diluted in 7 µL of 50 mM sodium phosphate buffer at pH 11.0 was analyzed by LC–MS and complete conversion to AnxV S-allyl Cys (calculated mass, 35846; observed mass, 35842) was observed. Small molecules were removed from the reaction mixture by loading the sample onto a Zeba™ Spin desalting column (ThermoFisher Scientific) previously equilibrated with PBS buffer at pH 7.6. The sample was eluted by centrifugation (2 min, 1500 x g). The protein sample (0.12 mg/mL by Bradford assay) was stored at –20 ºC.

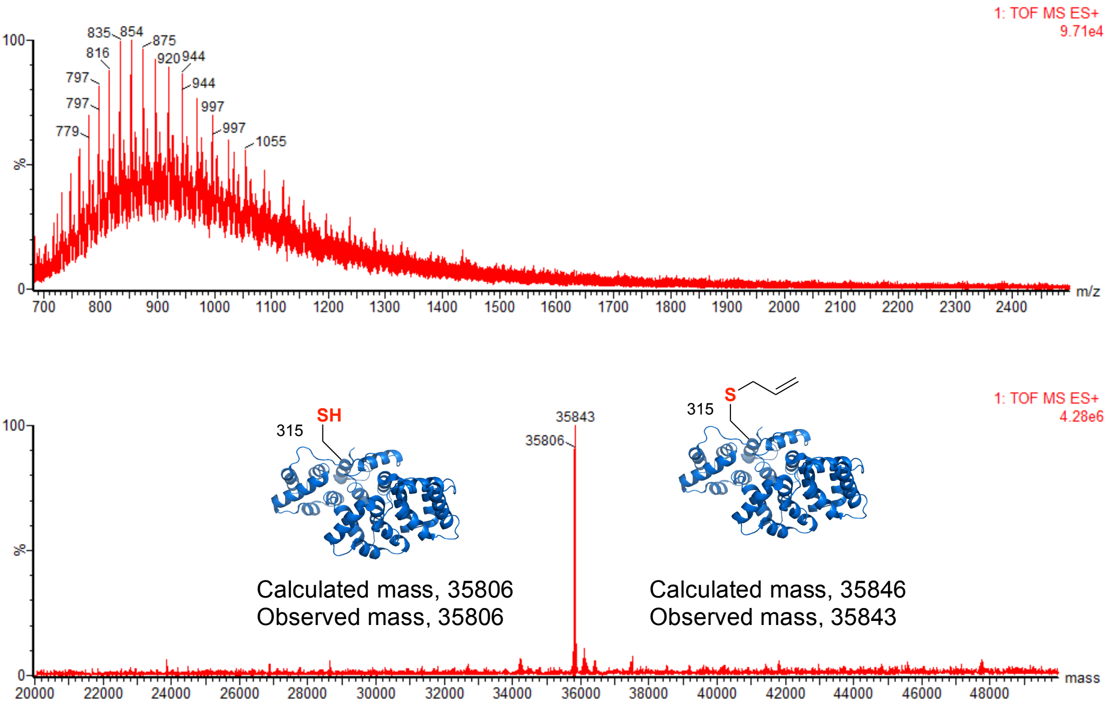

Figure S8 ESI–MS of the reaction of allyl chloride with AnxV after 1.5 h at 37 ºC.

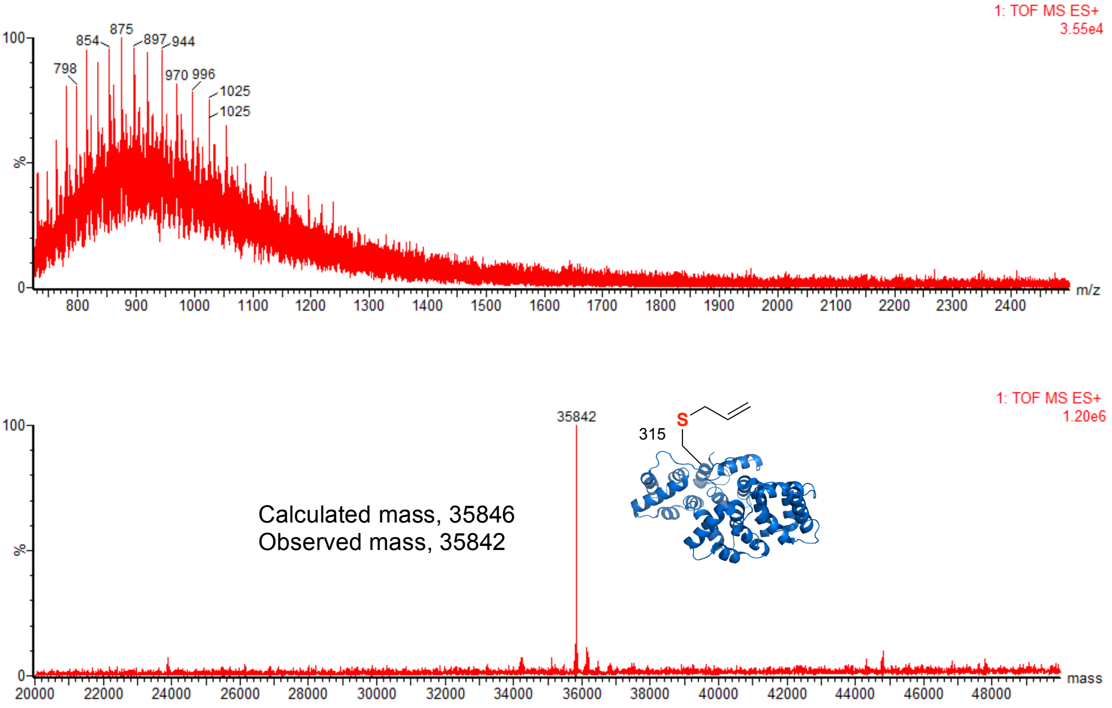

Figure S9 ESI–MS of the reaction of allyl chloride with AnxV after 3 h at 37 ºC.

2.4.3. Reaction of allyl selenocyanate with AnxV

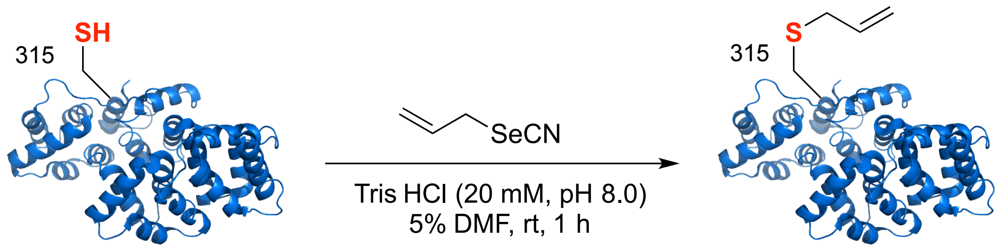

AnxV was prepared as a 1 mg/mL solution in 20 mM Tris HCl buffer at pH 8.0 and 0.5 mL (13.96 nmol) were transferred to a 1.5 mL eppendorf tube. Allyl selenocyanate (0.2 mg, 1396.4 nmol) in 26 µL DMF was added and the resulting mixture vortexed for 30 seconds. After 1 h of additional shaking, 3 µL aliquot diluted by 7 µL of 20 mM Tris HCl buffer at pH 8.0 was analyzed by LC–MS and complete conversion to AnxV S-allyl Cys (calculated mass, 35846; observed mass, 35846) was observed. Small molecules were removed from the reaction mixture by loading the sample onto a Zeba™ Spin desalting column (ThermoFisher Scientific) previously equilibrated with PBS buffer at pH 7.6. The sample was eluted by centrifugation (2 min, 1500 x g). The protein sample (0.91 mg/mL by Bradford assay) was stored at –20 ºC.

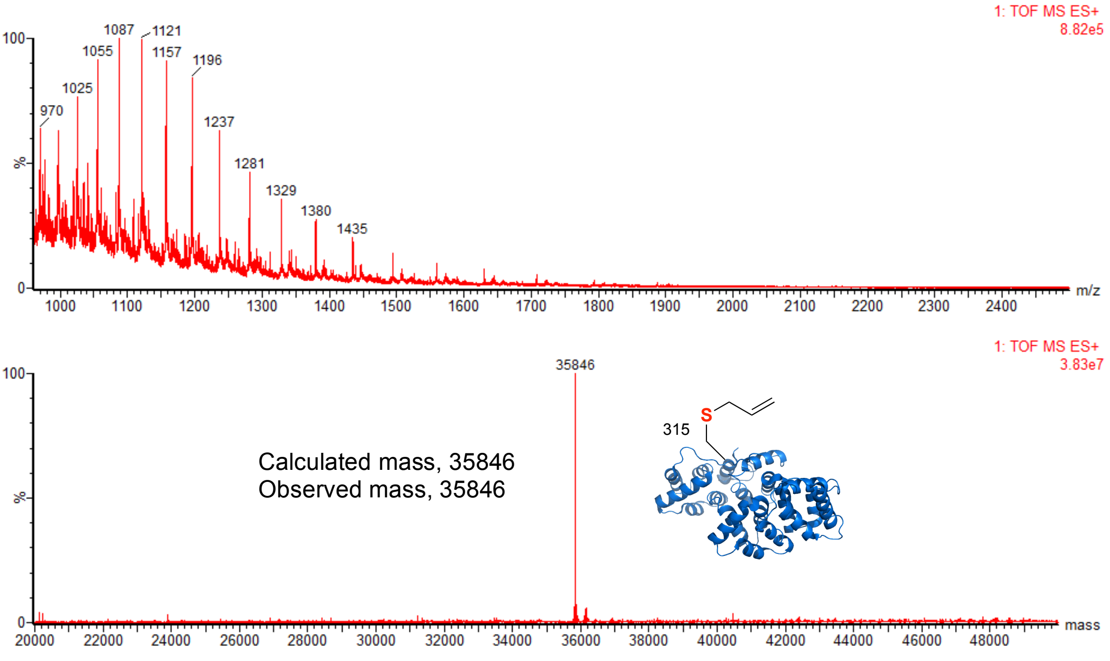

Figure S10 ESI–MS of the reaction of allyl selenocyanate with AnxV.

Control: reaction of Ellman's reagent with AnxV S-allyl Cys

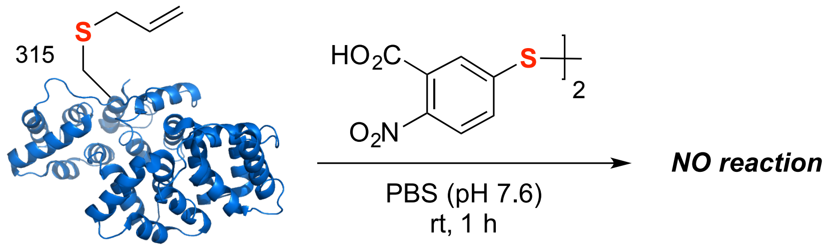

A 10 µL aliquot of AnxV S-allyl Cys (0.12 mg/mL, 0.0335 nmol) in PBS buffer at pH 7.6 was transferred to a 0.5 mL eppendorf tube. Ellman's reagent (0.2 µL of a 14 mg/mL stock solution in H_2O, 7.1 nmol) was added and the resulting mixture vortexed for 30 seconds. After 1 h of additional shaking, a 3 µL aliquot diluted in 7 µL of 50 mM sodium phosphate buffer at pH 7.6 was analyzed by LC–MS and starting protein AnxV S-allyl Cys (calculated mass, 35846; observed mass, 35846) was detected unaltered.

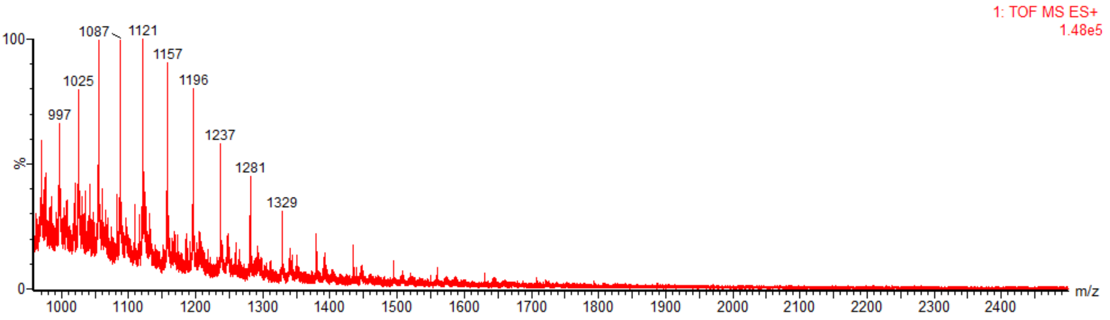

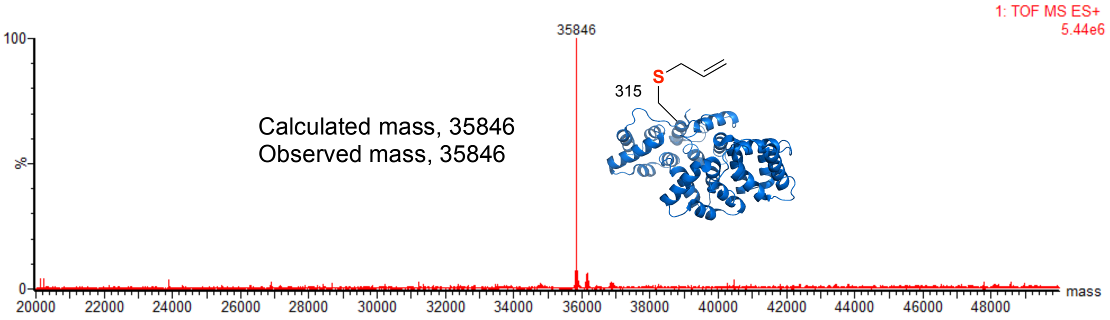

Figure S11 ESI–MS of the reaction of Ellman's reagent with AnxV S-allyl Cys.

2.4.4. Reaction of AnxV S-allyl Cys with Py-Tz 2

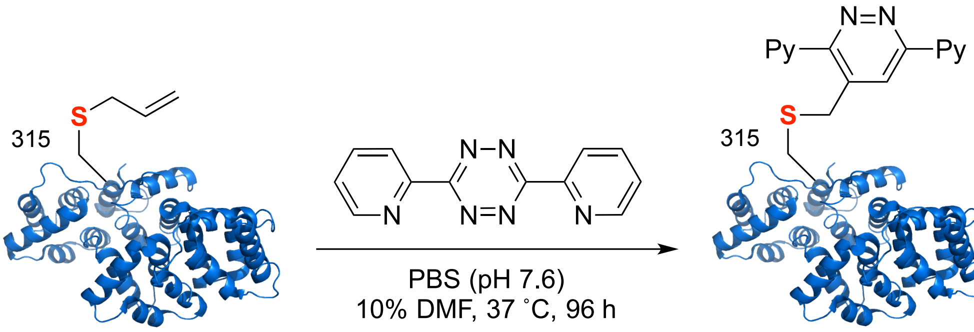

A 25 μL aliquot of AnxV S-allyl Cys (0.91 mg/mL, 0.635 nmol) in PBS buffer at pH 7.6 was transferred to a 0.5 mL eppendorf tube. Py-Tz **2** (3.1 μL of a stock solution of 0.4 mg in 41 μL DMF, 127 nmol) was added and the resulting mixture vortexed for 30 seconds. After 96 h of additional shaking, a 3 μL aliquot diluted by 7 μL of PBS buffer at pH 7.6 was analyzed by LC–MS and 70% conversion to the expected ligation product (AnxV S-allyl Cys-**2**) (calculated mass, 36052; observed mass, 36052) together with 20% of bis-ligation product (calculated mass, 36263; observed mass, 36262) was observed.

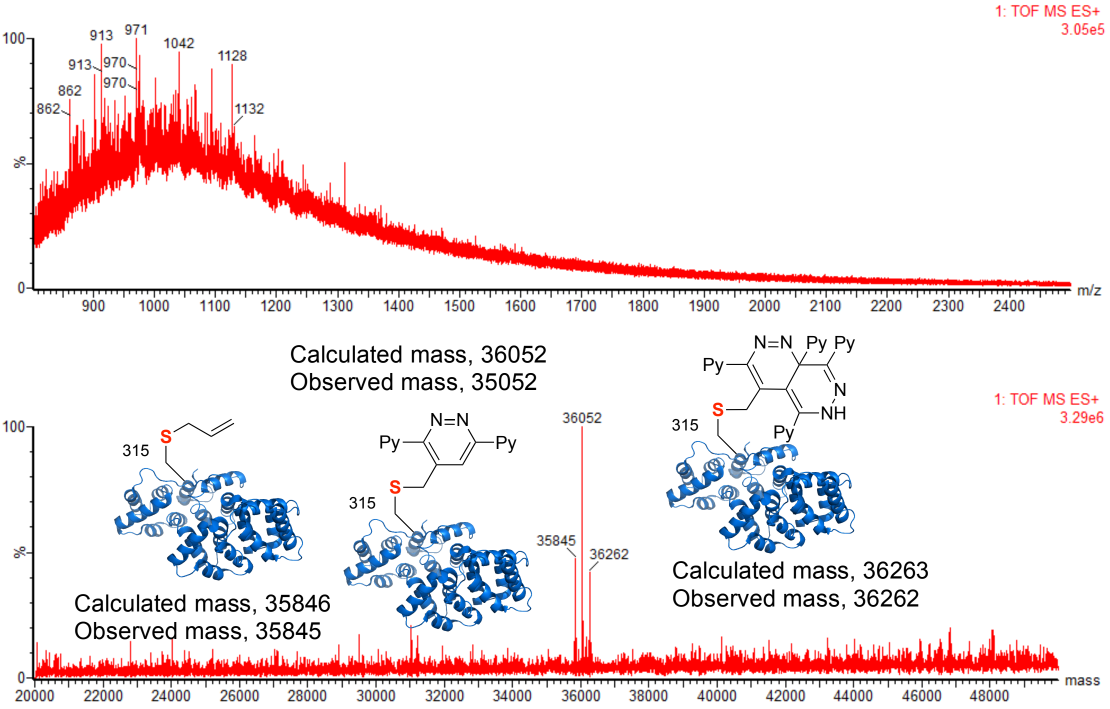

Figure S12 ESI–MS of the reaction of **2** with AnxV S-allyl Cys after 72 h at 37 ºC.

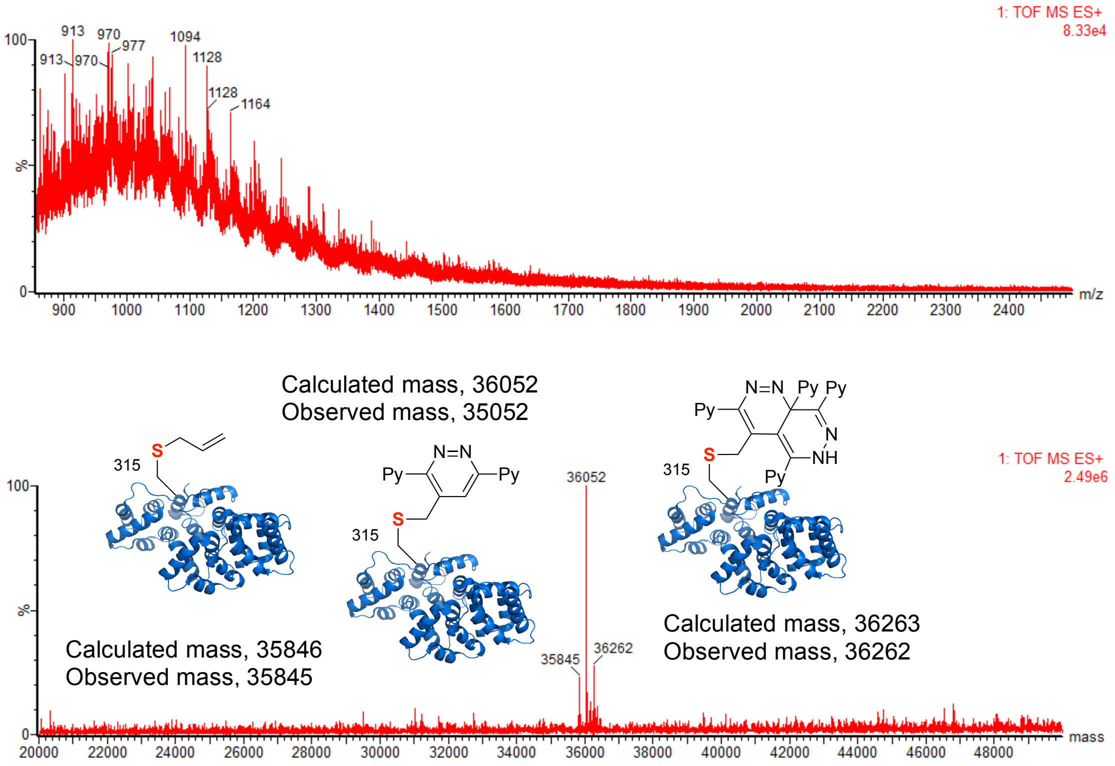

Figure S13 ESI–MS of the reaction of **2** with AnxV S-allyl Cys after 96 h at 37 ºC.

Reaction between AnxV S-allyl Cys and Py-Tz **2** was performed in triplicate with similar results (**Figure S14** and **S15)**.

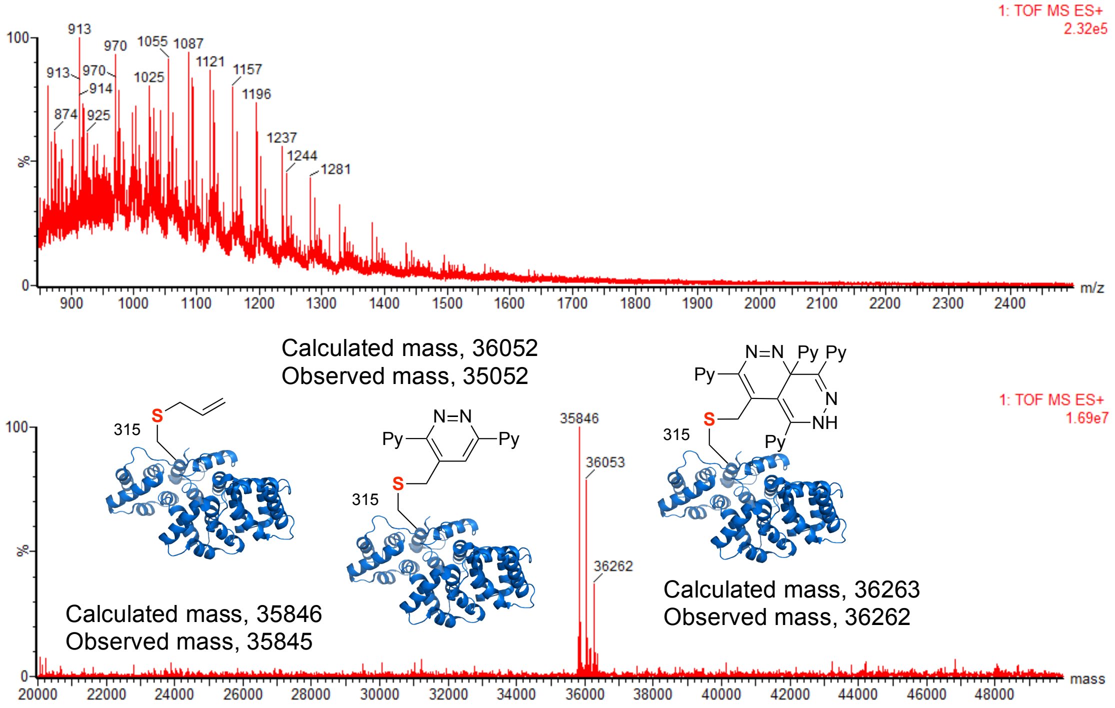

Figure S14 ESI–MS of the reaction of **2** with AnxV S-allyl Cys after 20 h at 37 ºC.

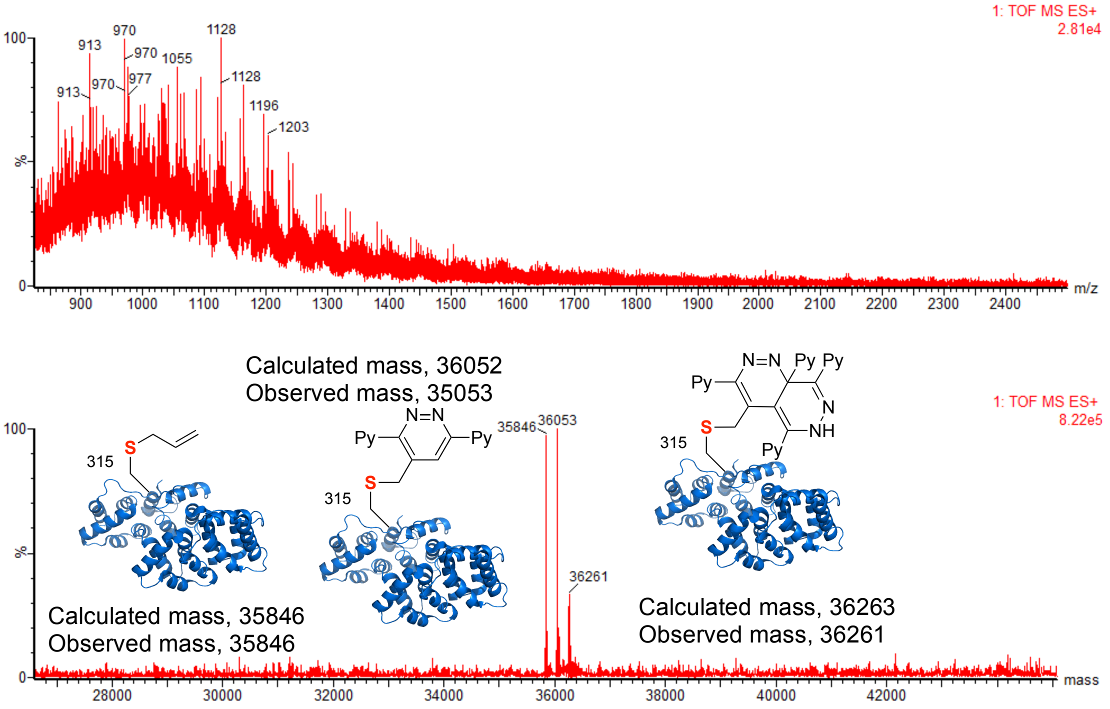

Figure S15 ESI–MS of the reaction of **2** with AnxV S-allyl Cys after 37 h at 37 ºC.

2.4.5. Reaction of tetrazine Bn-NH$_2$-Tz with AnxV S-allyl Cys

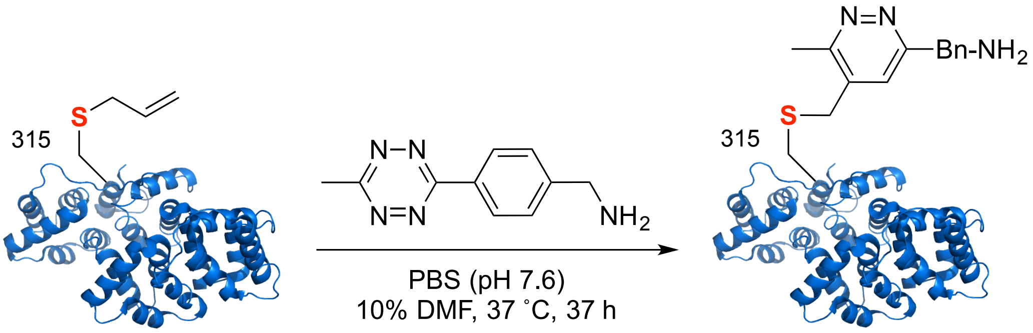

A 12.5 µL aliquot of AnxV S-allyl Cys (0.91 mg/mL, 0.317 nmol) in PBS buffer at pH 7.6 was transferred to a 0.5 mL eppendorf tube and diluted with 37.5 µL of PBS pH 7.6. Tetrazine (Bn-NH$_2$-Tz **3**) (63.5 nmol) was added in 6.3 µL DMF and the resulting mixture vortexed for 30 seconds. After 36 h of additional shaking at 37 ºC, a 8 µL aliquot was analyzed by LC–MS and conversion to the expected ligation product AnxV S-allyl Cys-**3** (calculated mass, 36052; observed mass, 36053) was observed.

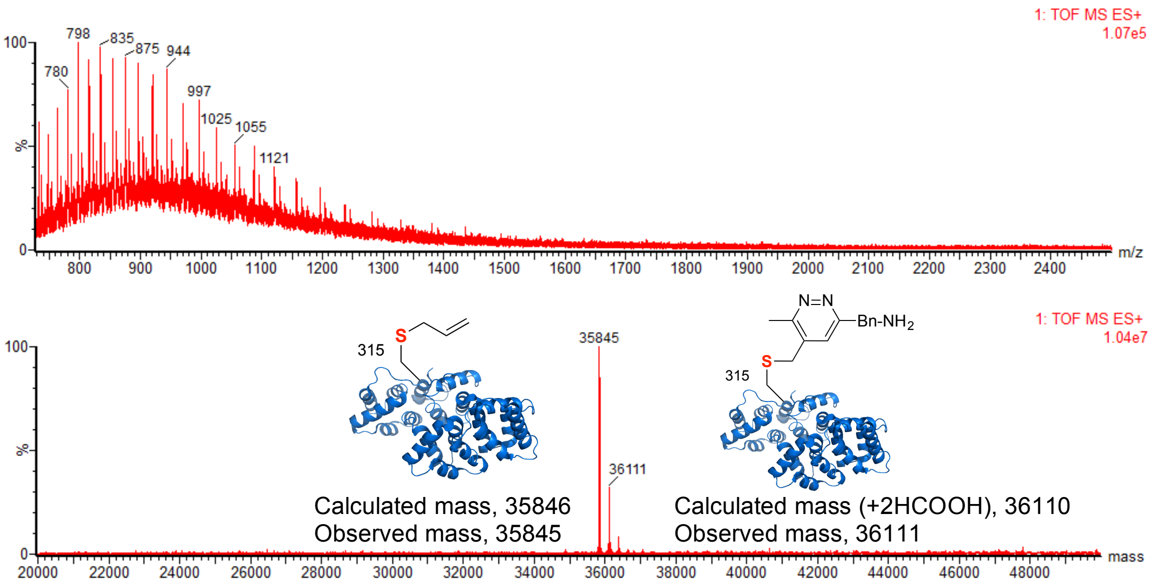

Figure S16. ESI–MS of the reaction of Bn-NH_2-Tz **3** with AnxV S-allyl Cys after 5 h at 37 ºC.

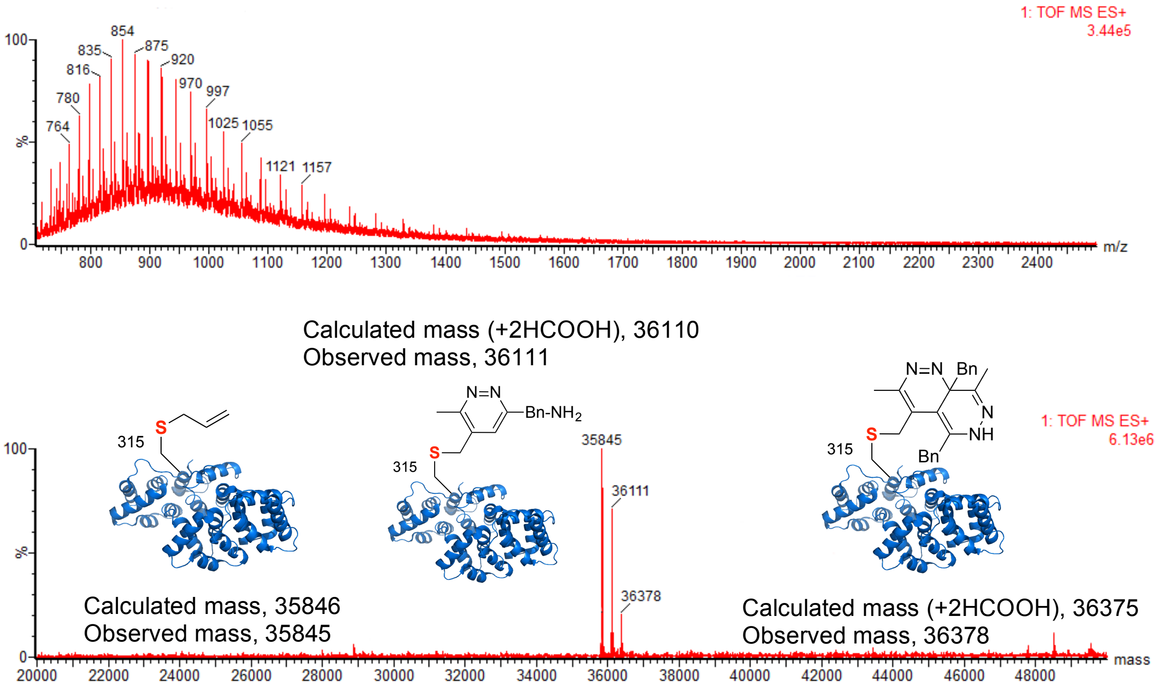

Figure S17. ESI–MS of the reaction of Bn-NH_2-Tz **3** with AnxV S-allyl Cys after 12 h at 37 ºC.

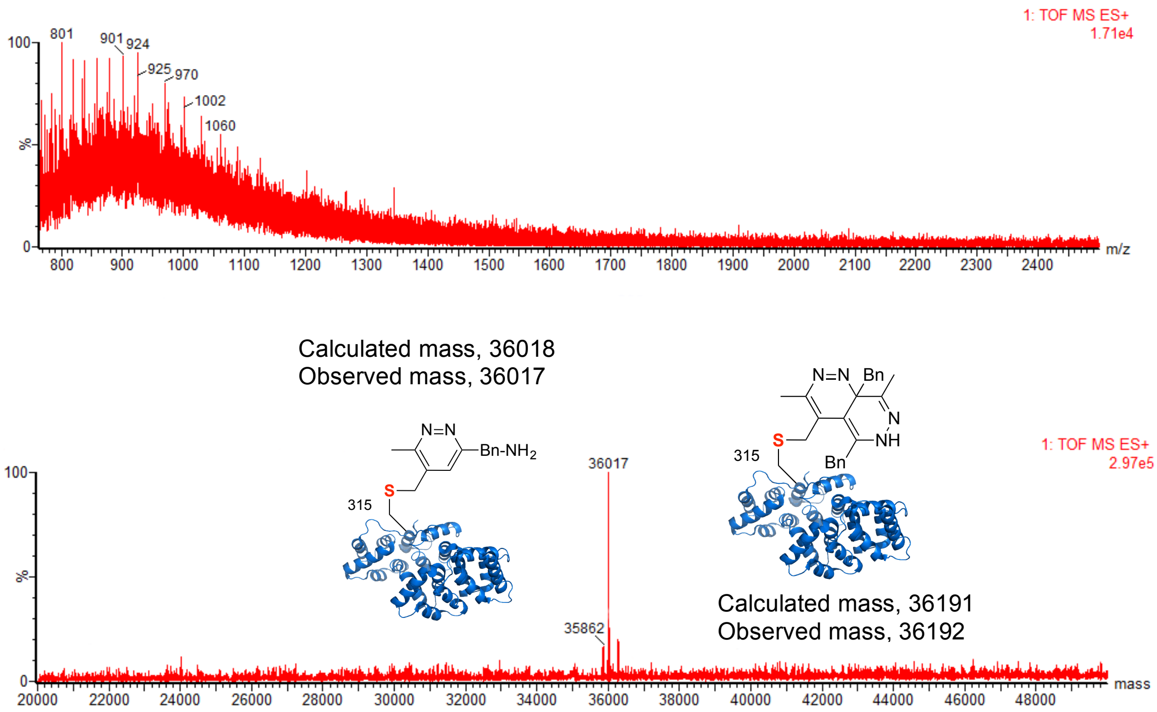

Figure S18 ESI–MS of the reaction of Bn-NH_2-Tz **3** with AnxV S-allyl Cys after 36 h at 37 ºC.

2.4.6. Control experiments for the formation of the bis-ligation product

Reaction of tetrazine Py-Tz 2 with AnxV

During the reaction between the tetrazine cores and AnxV S-allyl Cys it was observed the formation of a bis-ligation product. To check if the double addition was result of cross-reactivity with other amino acids on the protein, the tetrazine reaction was performed with the unmodified AnxV using the same conditions (200 eq, 37 °C, 96 h, **Figure S19**). These control experiments revealed that there is no ligation of the tetrazine to the protein if the allyl handle is not present precluding any cross-reactivity with other amino acid residues (controls for C2Am are shown in **Figure S33**).

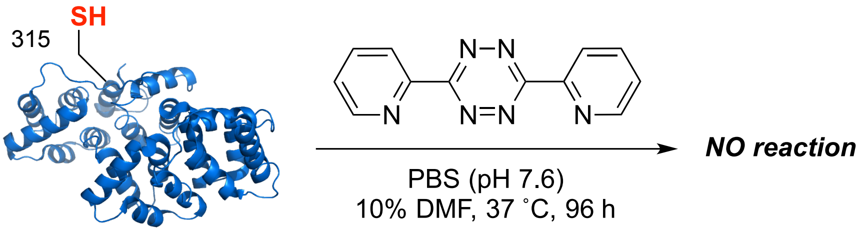

AnxV was prepared as a 1 mg/mL solution in PBS buffer at pH 7.6 and 25 µL (0.698 nmol) were transferred to a 0.5 mL eppendorf tube. Tetrazine Py-Tz **2** (2.8 µL of a stock solution of 0.4 mg in 34 µL DMF, 139.6 nmol) was added and the resulting mixture vortexed for 30 seconds. After 96 h of additional shaking at 37 ºC, a 3 µL aliquot diluted by 7 µL of PBS buffer at pH 7.6 was analyzed by LC–MS and starting protein AnxV (calculated mass, 35806; observed mass, 35805) was detected unaltered.

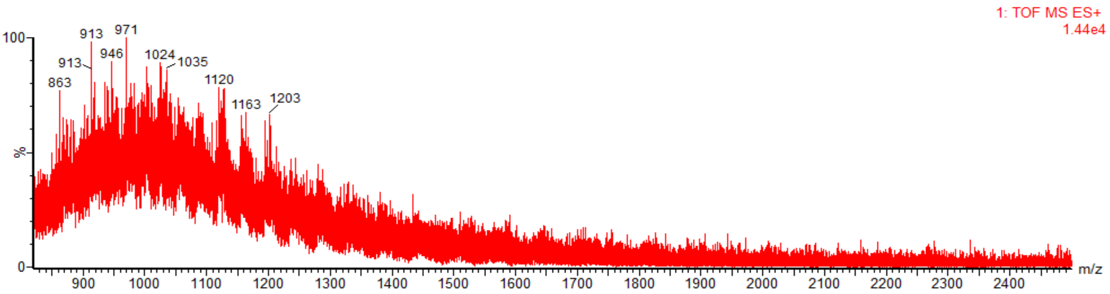

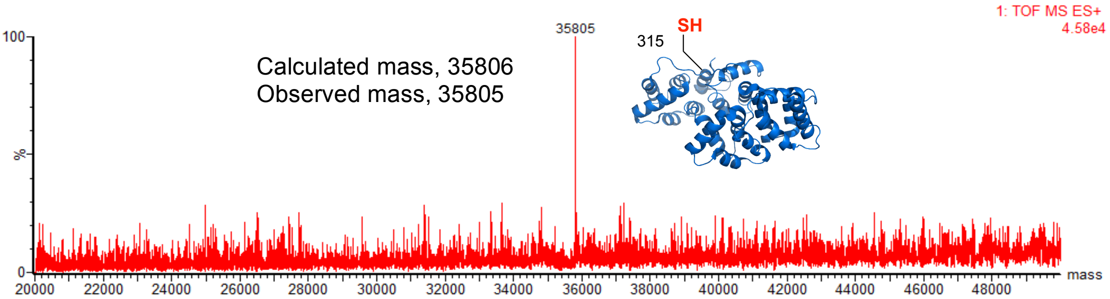

Figure S19 ESI–MS of the reaction of Py-Tz **2** with AnxV after 96 h at 37 ºC.

Reaction of tetrazine Py-Tz 2 with AnxV S-dimethylallyl Cys and AnxV S-propargyl Cys

Control experiments shown above revealed that there is no reaction between AnxV (no S-allyl present) and Py-Tz **2**. To further corroborated that the bis-addition is not cross-reactivity with other amino acids on the protein, the Cysteine 316 of AnxV was alkylated with dimethylallyl bromide and propargyl chloride (**Figures S20** and **S21**). This control was to verify if modification of the cysteine may induce conformational changes in the structure of the protein, exposing any buried amino acid that could potentially react with the tetrazine. As observed before, upon reaction of AnxV S-dimethylallyl Cys (**Figure S22**) and AnxV S-propargyl Cys with Py-Tz **2** (**Figure S23**) the starting protein was detected unaltered after 48 h at 37 °C.

Reaction of 3,3-dimethylallyl bromide with AnxV

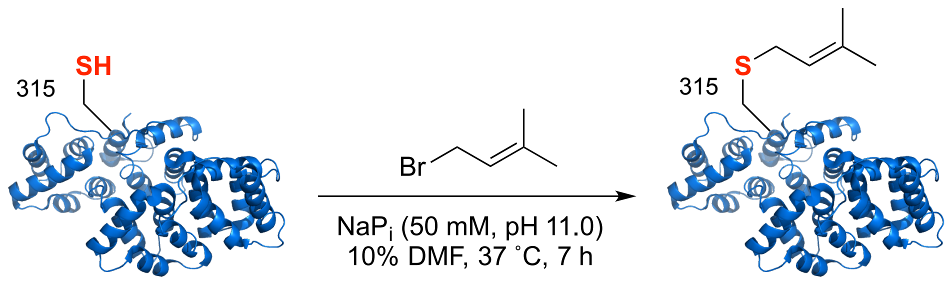

AnxV was prepared as a 0.12 mg/mL solution in 50 mM sodium phosphate buffer at pH 11.0 and 50 µL (0.168 nmol) were transferred to a 0.5 mL eppendorf tube. 3,3-Dimethylallyl bromide (5.6 µL of a 1.4 mg stock solution in 607 µL DMF, 83.8 nmol) was added and the resulting mixture vortexed for 30 seconds. After 7 h of additional shaking at 37 ºC, a 3 µL aliquot diluted by 7 µL of 50 mM sodium phosphate buffer at pH 11.0 was analyzed by LC–MS and conversion to AnxV S-dimethylallyl Cys (calculated mass, 35874; observed mass, 35872) was observed. Small molecules were removed from the reaction mixture by loading the sample onto a Zeba™ Spin desalting column (ThermoFisher Scientific) previously equilibrated with PBS buffer at pH 7.6. The sample was eluted by centrifugation (2 min, 1500xg) and stored at –20 ºC.

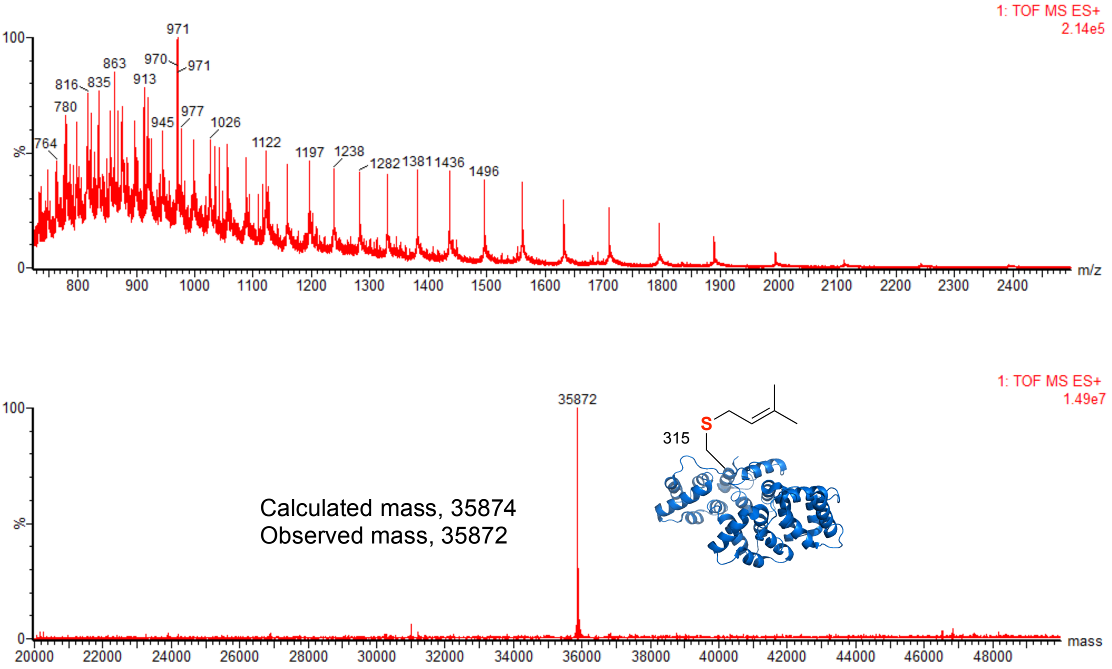

Figure S20 ESI–MS of the reaction of 3,3-dimethylallyl bromide with AnxV.

Reaction of propargyl chloride with AnxV

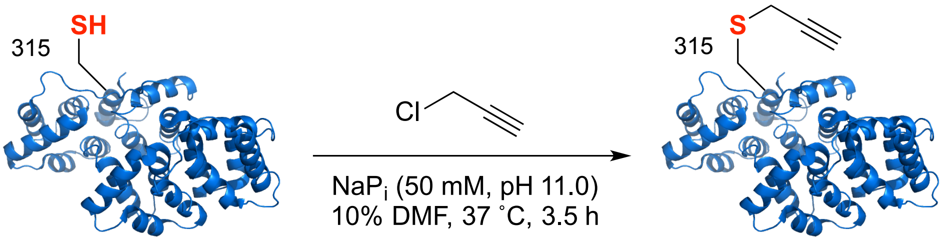

AnxV was prepared as a 0.4 mg/mL solution in 50 mM sodium phosphate buffer at pH 11.0 and 50 µL (0.559 nmol) were transferred to a 0.5 mL eppendorf tube. Propargyl chloride (5.6 µL of a stock solution of 0.5 mg in 140 µL DMF, 279.3 nmol) was added and the resulting mixture vortexed for 30 seconds. After 3.5 h of additional shaking at 37 ºC, a 3 µL aliquot diluted by 7 µL of 50 mM sodium phosphate buffer at pH 11.0 was analyzed by LC–MS and conversion to AnxV S-propargyl Cys (calculated mass, 35844; observed mass, 35844) was observed. Small molecules were removed from the reaction mixture by loading the sample onto a Zeba^{TM} Spin desalting column (ThermoFisher Scientific) previously equilibrated with PBS buffer at pH 7.6. The sample was eluted by

centrifugation (2 min, 1500 x g) and stored at –20 ºC. No reaction was observed with Ellman's reagent indicating that propargylation of the cysteine was complete. Alkylation of AnxV with propargyl chloride at pH 8 resulted in very low conversions even with extended reaction times (>16 h).

Formation of allenes from propargyl halides via a SN2' mechanism may occur under certain conditions. To support that S-propargylation occurs instead of S-allenation, BocNH-Cys-OMe (0.021 mmol) was reacted with propargyl chloride (3 eq) for 18 h at 37 ºC in 10% DMF/PBS at pH 11 (identical conditions of those used for protein modification). This afforded the propargylated protected Cys in 93% yield after purification by flash column chromatography.

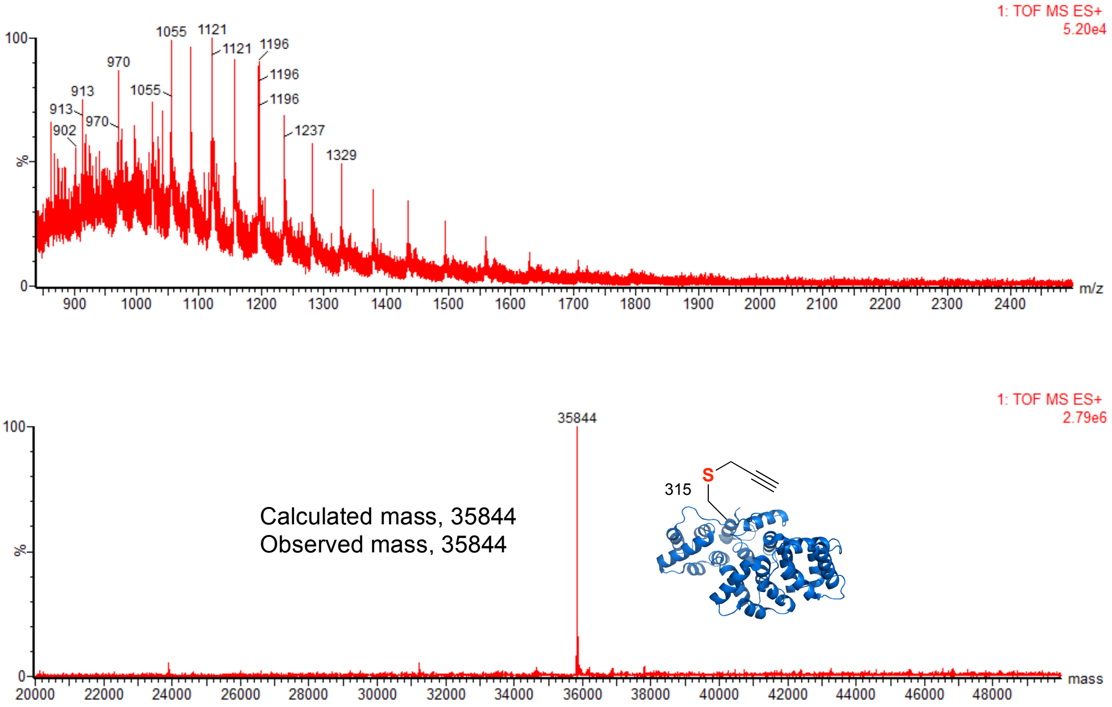

Figure S21 ESI–MS of the reaction of propargyl chloride with AnxV after 3.5 h at 37 ºC.

***Reaction of tetrazine Py-Tz with* AnxV S-dimethylallyl Cys**

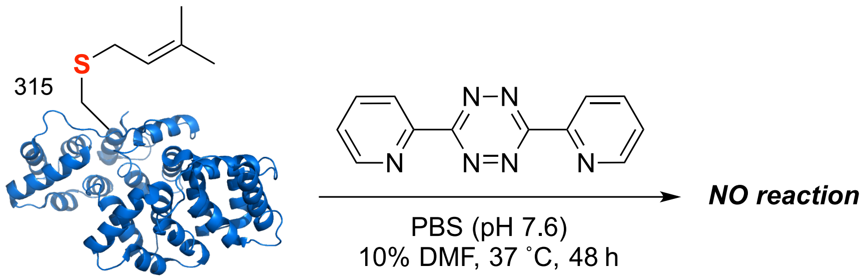

A 50 μL aliquot of AnxV S-dimethylallyl Cys (0.116 mg/mL, 0.166 nmol) in PBS buffer at pH 7.6 was transferred to a 0.5 mL eppendorf tube. Py-Tz **2** (500 eq, 5.6 μL of a stock solution of 1.6 mg in 1.1 mL DMF, 33.2 nmol) was added and the resulting mixture vortexed for 30 seconds. After 48 h of additional shaking at 37 ºC, a 8 μL aliquot was analyzed by LC–MS and starting protein (calculated mass, 35874; observed mass, 35872) was detected unaltered.

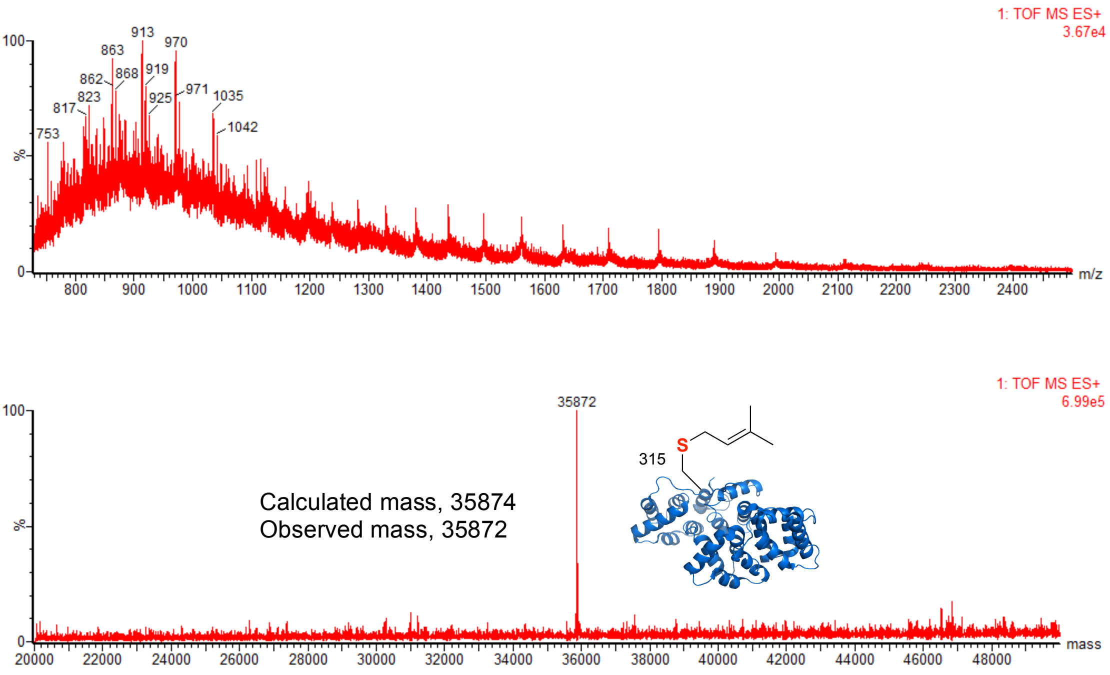

Figure S22 ESI–MS of the reaction of Py-Tz **2** with AnxV S-dimethylallyl Cys after 48 h at 37 ºC.

Reaction of tetrazine Py-Tz with AnxV S-propargyl Cys

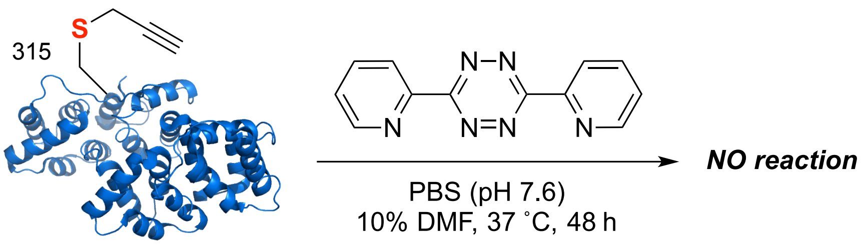

A 25 μL aliquot of AnxV S-propargyl Cys (0.4 mg/mL, 0.279 nmol) in PBS buffer at pH 7.6 was transferred to a 0.5 mL eppendorf tube. Py-Tz **2** (500 eq, 2.8 μL of a stock solution of 0.6 mg in 50 μL DMF, 139.6 nmol) was added and the resulting mixture vortexed for 30 seconds. After 48 h of additional shaking at 37 ºC, a 8 μL aliquot was analyzed by LC–MS and starting protein (calculated mass, 35844; observed mass, 35844) was detected unaltered.

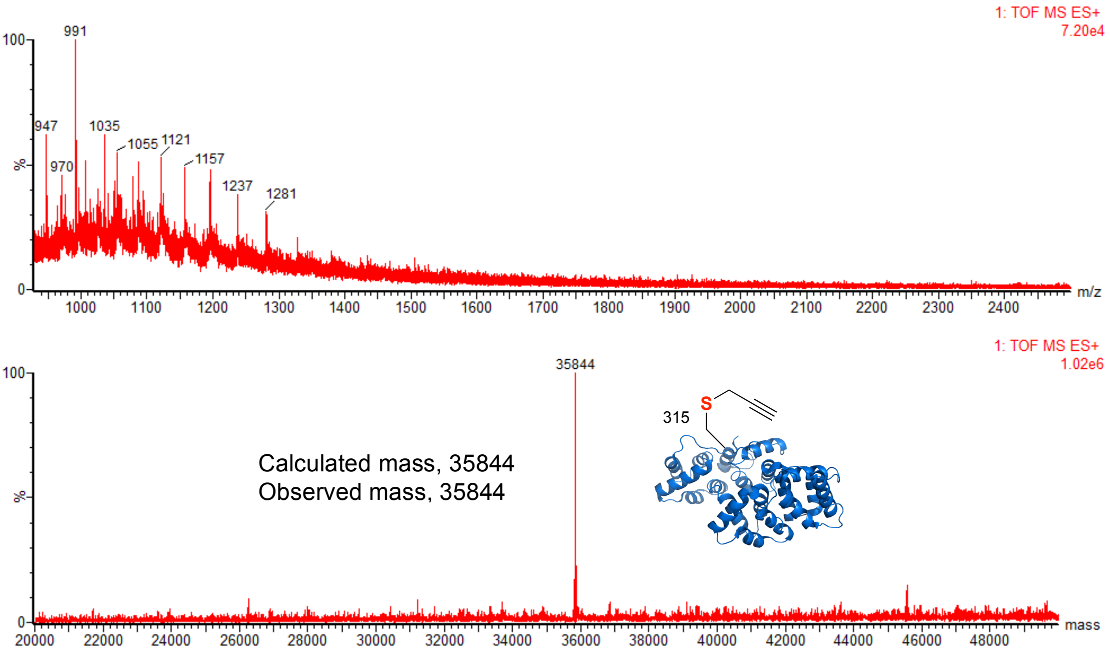

Figure S23 ESI–MS of the reaction of Py-Tz **2** with AnxV S-propargyl Cys after 48 h at 37 ºC.

2.4.7. Reaction of tetrazine-dye Tz-Rhod 4 with AnxV S-allyl Cys

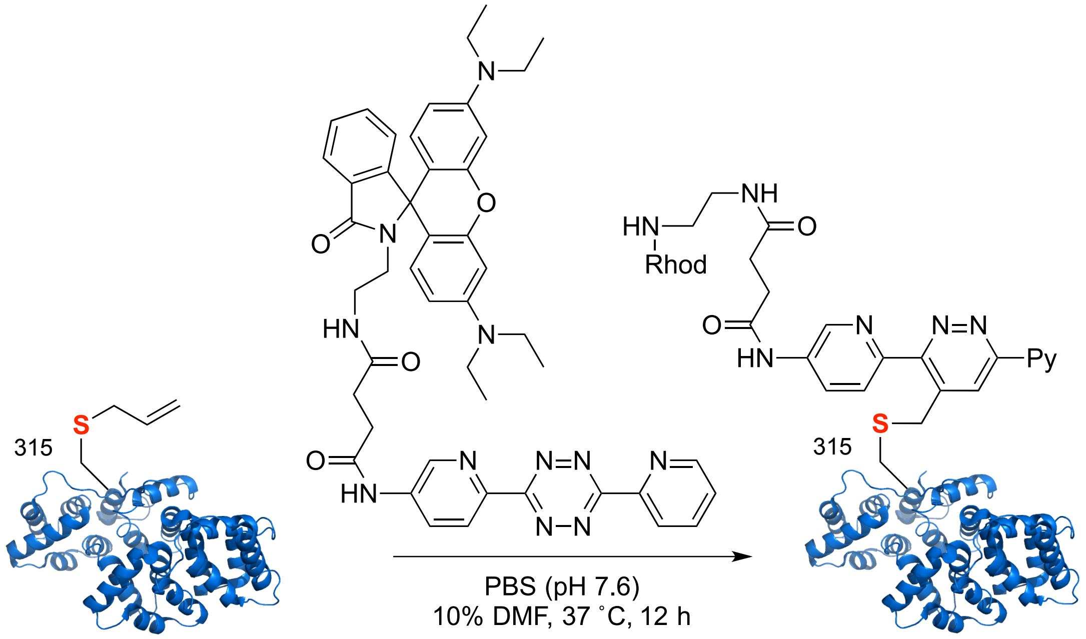

AnxV S-allyl Cys was prepared as a 0.19 mg/mL solution in PBS buffer at pH 7.6 and 60 µL (0.318 nmol) were transferred to a 0.5 mL eppendorf tube. Tz-Rhod **4** (200 eq, 6.2 µL of a stock solution 0.5 mg in 60 µL DMF, 63.6 nmol) was added and the resulting mixture vortexed for 30 seconds. After 12 h of additional shaking at 37 ºC, a 8 µL aliquot was analyzed by LC–MS and the expected ligation product (calculated mass, 36634; observed mass, 36634) was observed. Longer reaction periods led to low detection of the modified protein due to significant protein precipitation or less efficient ionization of the protein with the fluorophore attached.

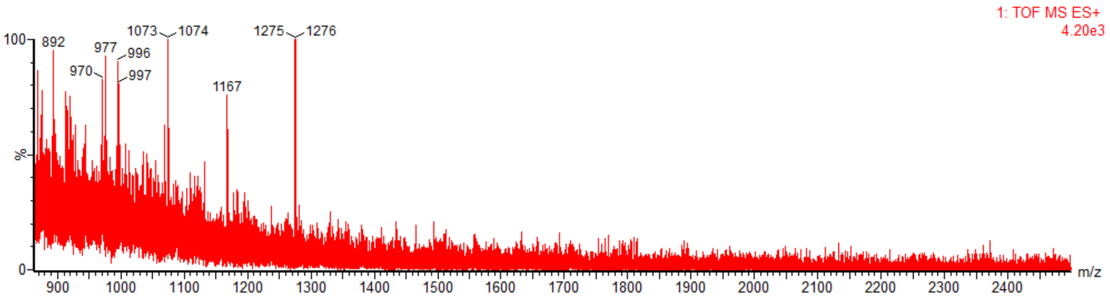

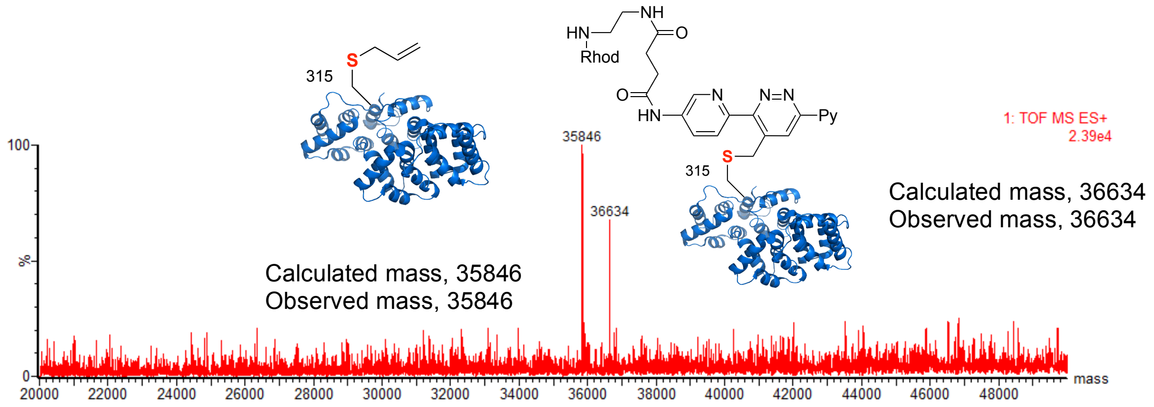

Figure S24 ESI–MS of the reaction of Tz-Rhod **4** with AnxV S-allyl Cys after 12 h at 37 ºC.

2.4.8. Reaction of tetrazine-dye Tz-Cy3 5 with AnxV S-allyl Cys

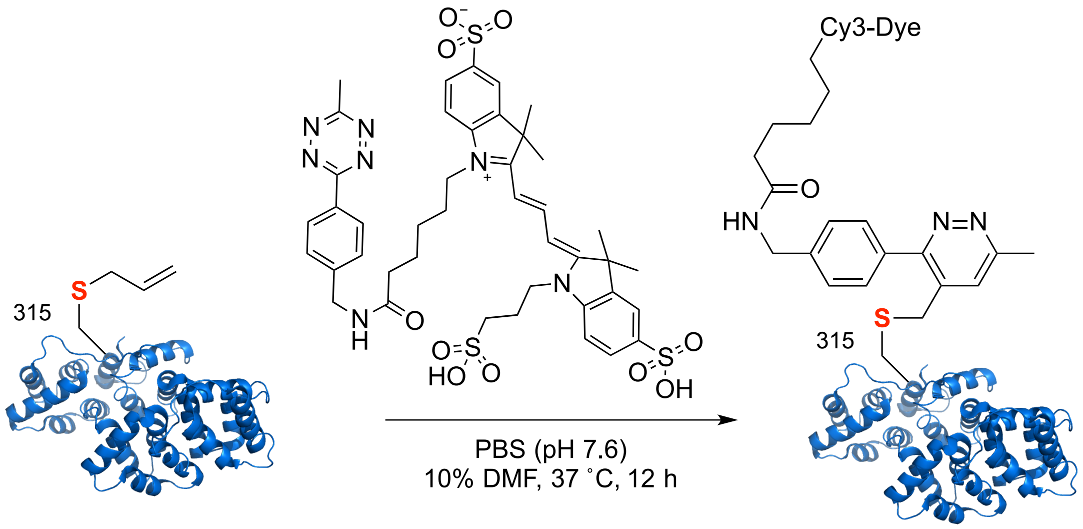

AnxV S-allyl Cys was prepared as a 0.19 mg/mL solution in PBS buffer at pH 7.6 and 60 µL (0.318 nmol) were transferred to a 0.5 mL eppendorf tube. Tz-Cy3 **5** (200 eq, 25 µL of a stock solution of 1 mg in 440 µL PBS pH 7.6, 63.0 nmol) was added and the resulting mixture vortexed for 30 seconds. After 12 h of additional shaking at 37 ºC, a 8 µL aliquot was analyzed by LC–MS and the expected ligation product (calculated mass, 36750; observed mass, 36753) was observed.

Note: The low signal/noise ration observed may be attributed to deficient protein ionization due to the presence of the conjugated dye.

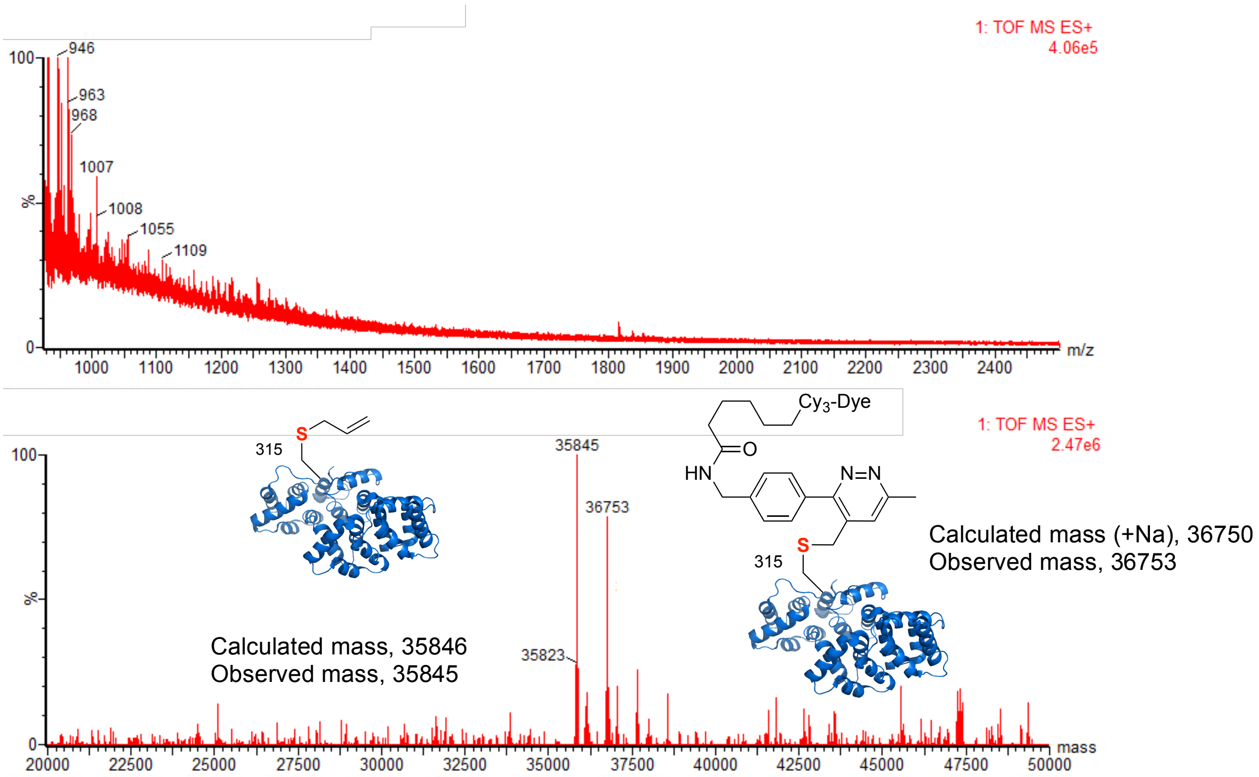

Figure S25 ESI–MS of the reaction of Tz-Cy3 **5** with AnxV S-allyl Cys after 12 h at 37 ºC.

2.5. C2Am protein modification analysis by LC-MS

2.5.1. Sequence of C2A domain of Synaptotagmin-I (C2Am, modified residue highlighted)[3]

GSPGISGGGGGILDSMVEKLGKLQYSLDYDFQNNQLLVGIIQAAELPALDMGGT
SDPYVKVFLLPDKKKKFETKVHRKTLNPVFNEQFTFKVPYCELGGKTLVMAVY
DFDRFSKHDII GEFKVPMNTVDFGHVTEEWRDLQSAEK

Calculated average isotopic mass = 16222.53 (*N*-terminal Met cleaved)

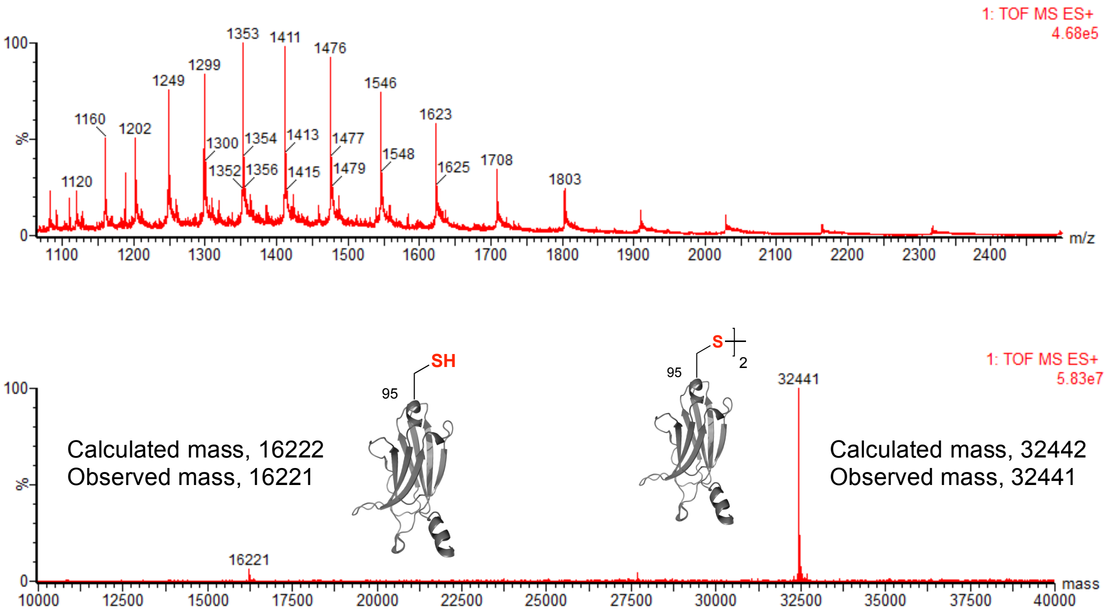

Figure S26 ESI–MS spectrum of non-reduced C2Am.

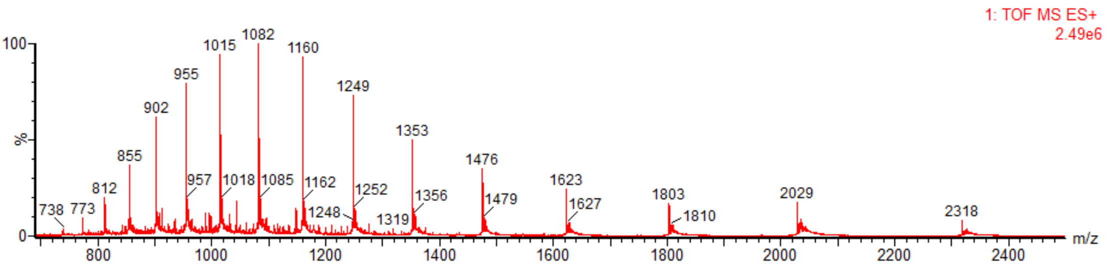

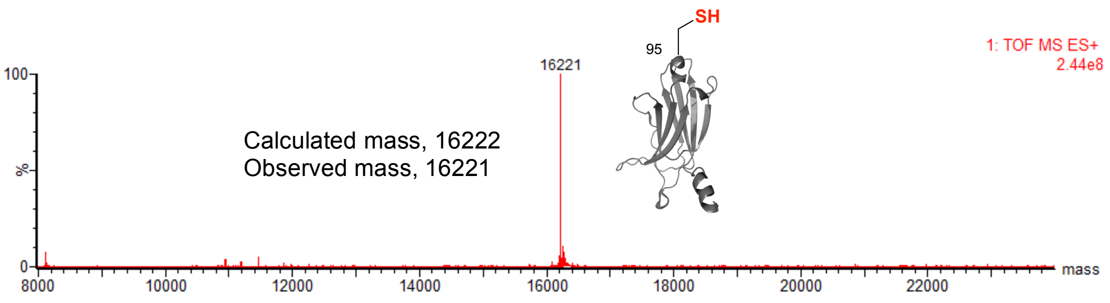

Figure S27 ESI–MS of reduced C2Am.

2.5.2. Reaction of allyl selenocyanate with C2Am

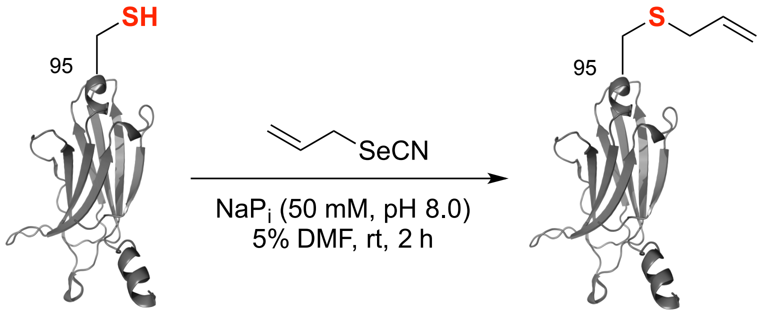

Tris(2-carboxyethyl)phosphine hydrochloride (TCEP·HCl) (100 eq, 10 µL of a stock solution of 1 mg/mL in water, 308.2 nmol) was added to a solution of C2Am (50 µL, 1 mg/mL, 3.08 nmol) in 50 mM sodium phosphate buffer at pH 8.0 and the resulting mixture shaken for 2 h at room temperature. Small molecules were removed from the reaction mixture by using a Zeba^{TM} Spin desalting column (ThermoFisher Scientific) previously equilibrated with 50 mM sodium phosphate buffer at pH 8.0. The sample was eluted by centrifugation (2 min, 1500 x g). A 25 µL aliquot of C2Am (*ca.* 1 mg/mL, 1.54 nmol) was transferred to a 0.5 mL eppendorf tube. Allyl selenocyanate (150 eq, 1.3 µL of a stock solution of 4.1 mg in 158 µL DMF, 231.2 nmol) was added and the resulting mixture vortexed for 30 seconds. After 2 h of additional shaking, a 3 µL aliquot diluted by 7 µL of 50 mM sodium phosphate buffer at pH 8.0 was analyzed by LC–MS and complete conversion to C2Am S-allyl Cys (calculated mass, 16262; observed mass, 16263) was observed. Small molecules were removed from the reaction mixture by using a Zeba^{TM} Spin desalting column (ThermoFisher Scientific) equilibrated with PBS buffer at pH 7.6. The product protein did not react with Ellman's reagent, indicating that all

cysteine was consumed in the allylation reaction. The protein sample (0.32 mg/mL by Bradford assay) was stored at –20 ºC.

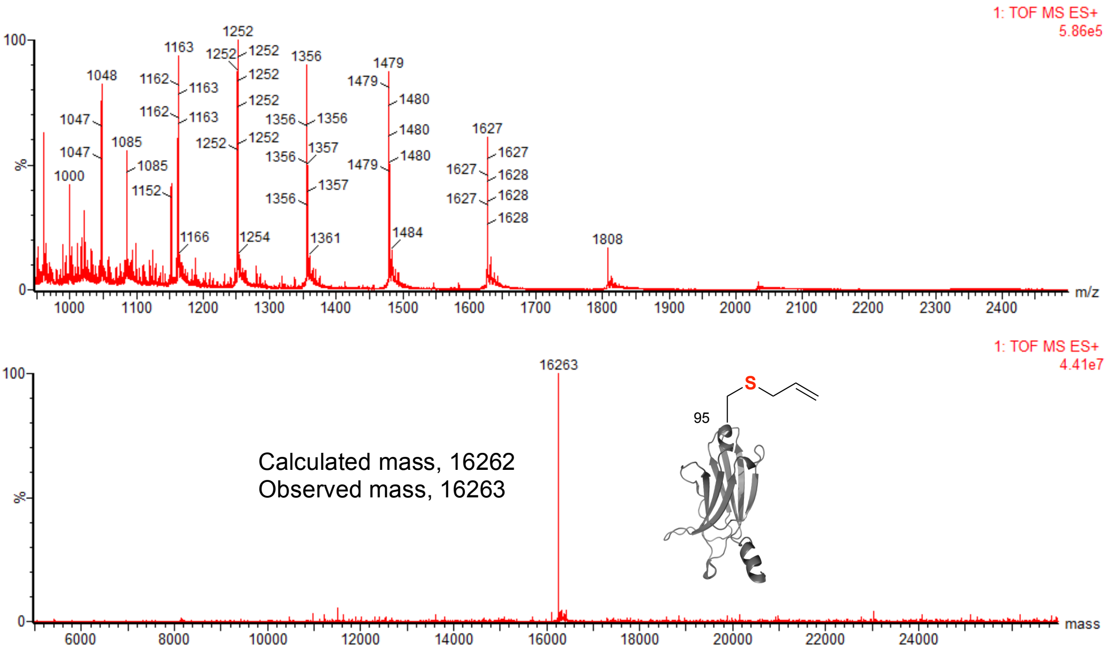

Figure S28 ESI–MS of the reaction of allyl selenocyanate with C2Am after 2 h at room temperature.

2.5.3. Reaction of tetrazine Py-Tz 2 with C2Am S-allyl Cys

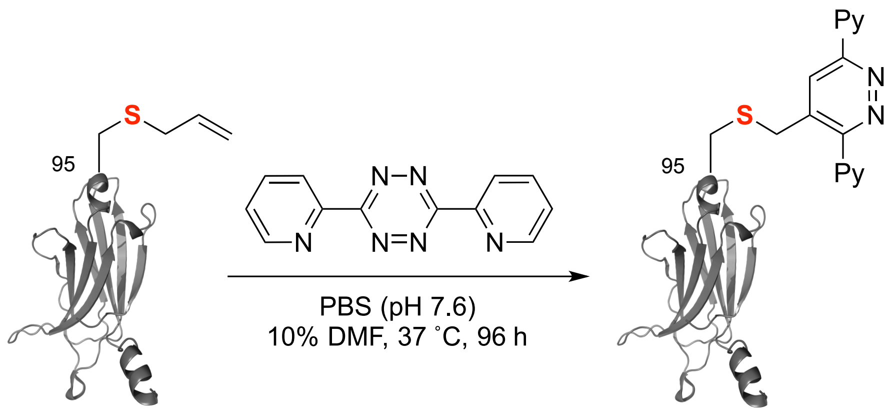

A 25 μL aliquot of C2Am S-allyl Cys (0.32 mg/mL, 0.492 nmol) in PBS buffer at pH 7.6 was transferred to a 0.5 mL eppendorf tube. Py-Tz **2** (200 eq, 2.8 μL of a stock solution of 0.4 mg in 48 μL DMF, 98.4 nmol) was added and the resulting mixture vortexed for 30 seconds. After 96 h of additional shaking at 37 ºC, a 3 μL aliquot diluted by 7 μL of

PBS buffer at pH 7.6 was analyzed by LC–MS and complete conversion to the expected ligation product (calculated mass, 16466; observed mass, 16468) together with 10% of bis-ligation product (calculated mass, 16674; observed mass, 16679) was observed.

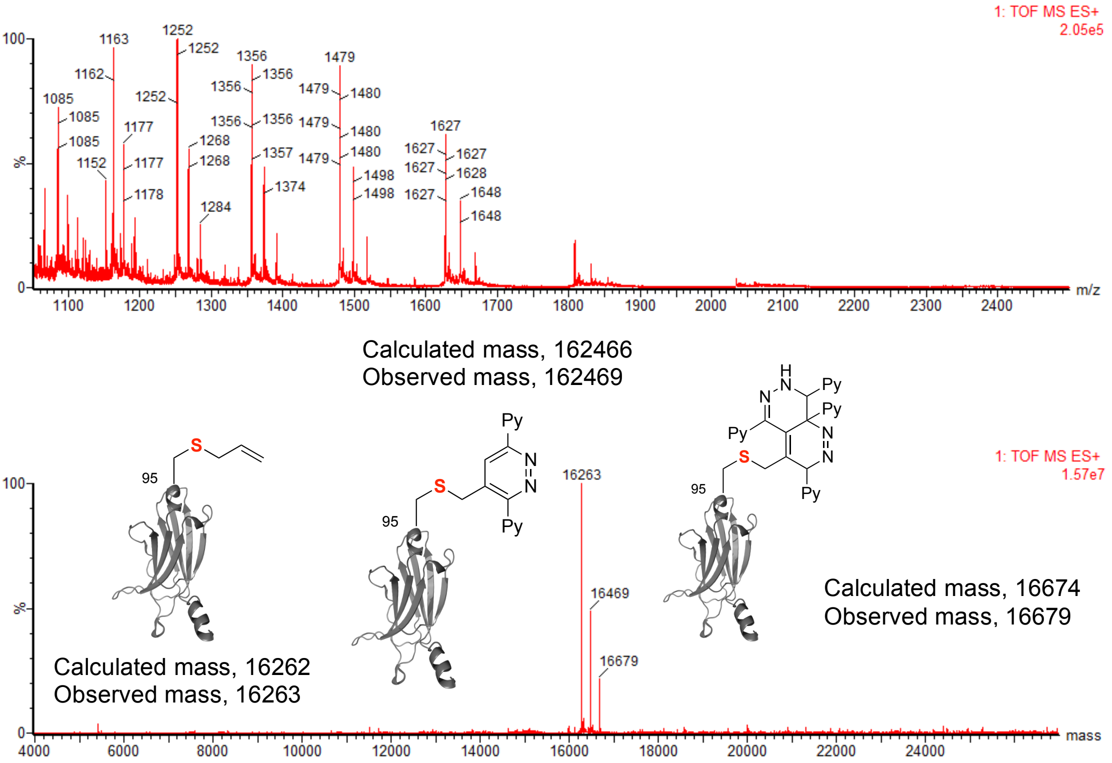

Figure S29 ESI–MS of the reaction of **2** with C2Am S-allyl Cys after 24 h at 37 ºC.

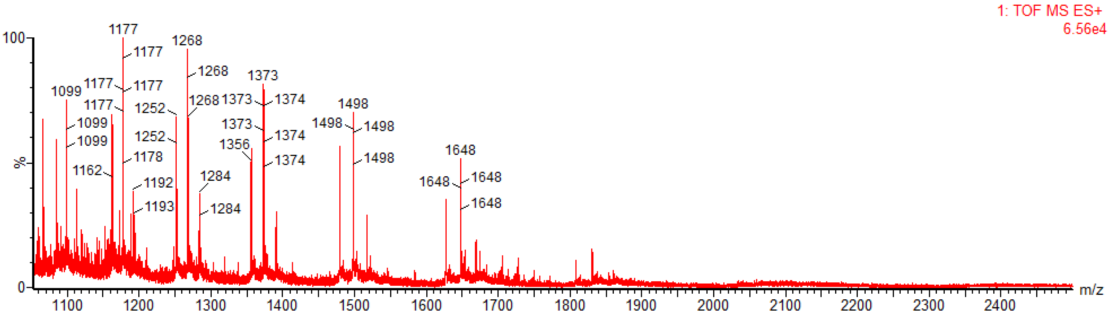

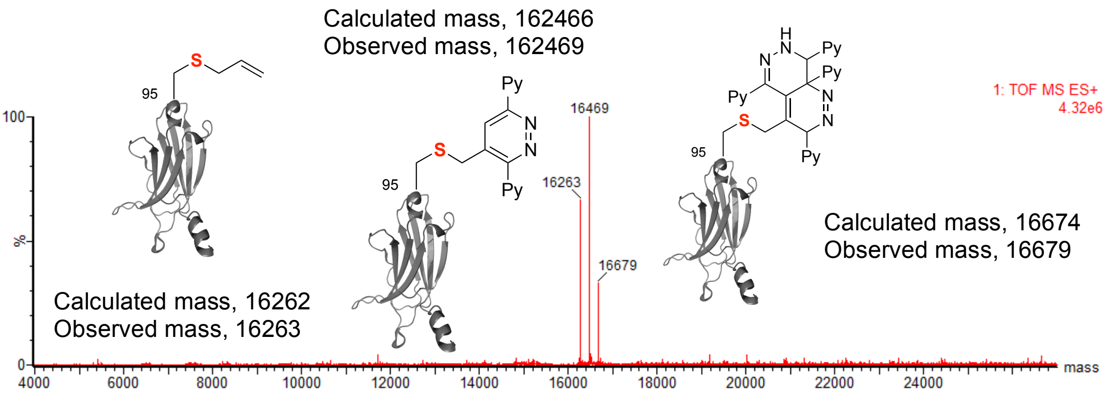

Figure S30 ESI–MS of the reaction of **2** with C2Am S-allyl Cys after 48 h at 37 ºC.

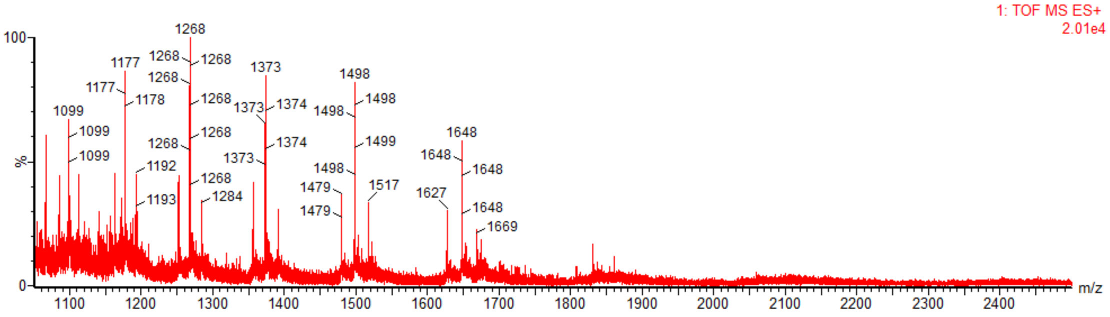

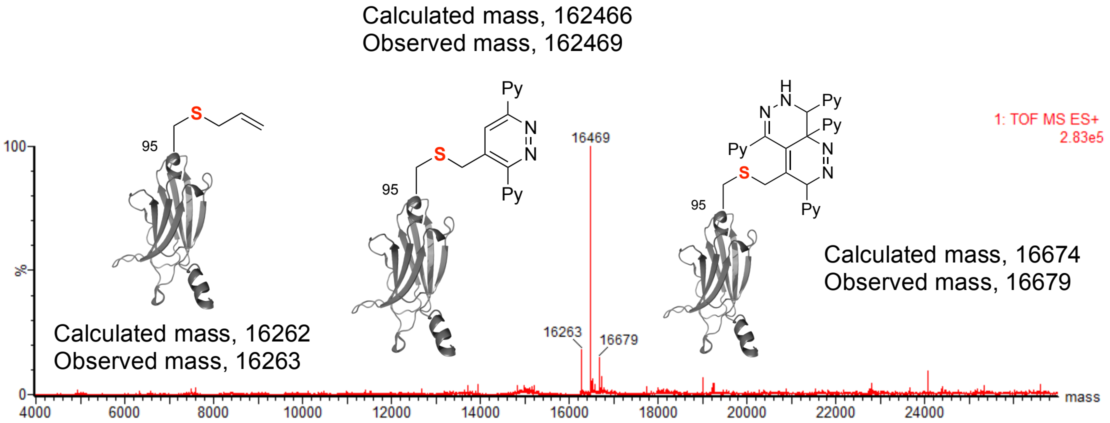

Figure S31 ESI–MS of the reaction of **2** with C2Am S-allyl Cys after 72 h at 37 ºC.

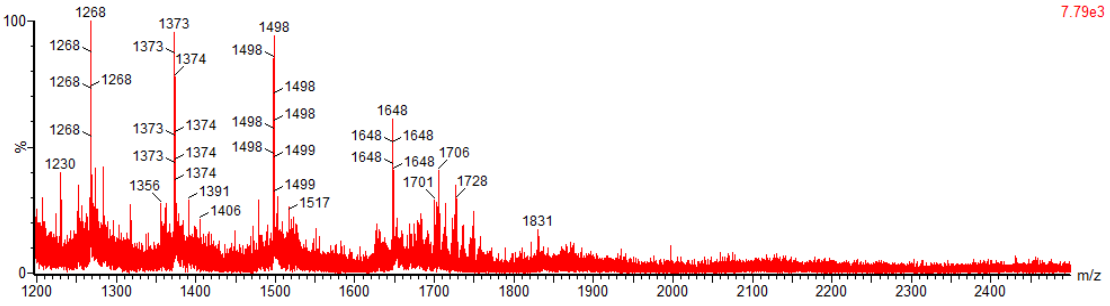

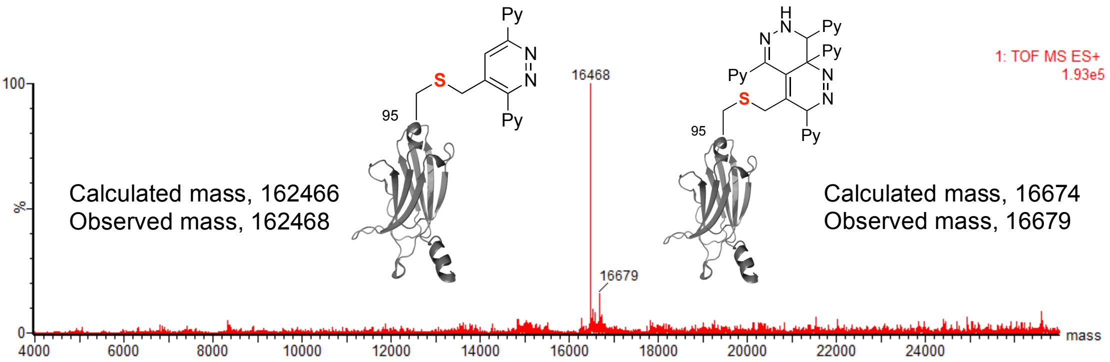

Figure S32 ESI–MS of the reaction of **2** with C2Am S-allyl Cys after 96 h at 37 ºC.

2.5.4. Control: reaction of tetrazine Py-Tz 2 with C2Am

Reaction of the "S-allyl proteins" with the tetrazine cores resulted in the formation of a subproduct corresponding to the addition of two tetrazine moieties. To check if the bis-addition is not cross-reactivity with other amino acids on the C2Am, the untagged protein was reacted with Py-Tz **2** under the same conditions (200 eq, 37 °C, 96 h). As previously observed for AnxV (**Section 2.4.6.**) no reaction was observed if the allyl handle is not present suggesting no cross-reactivity with other amino acid residues.

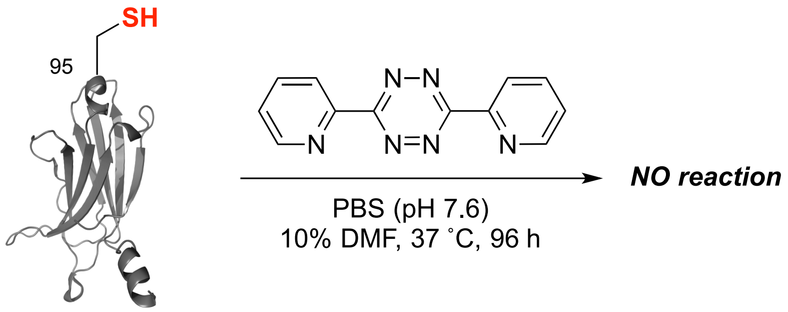

Tris(2-carboxyethyl)phosphine hydrochloride (TCEP·HCl) (11 eq, 10 µL of a stock solution of 1 mg/mL in water, 35.0 nmol) was added to a solution of C2Am (50 µL, 1 mg/mL, 3.08 nmol) in 50 mM sodium phosphate buffer at pH 8.0 and the resulting mixture shaken for 2 h. Small molecules were removed using Zeba™ Spin desalting column (ThermoFisher Scientific). A 25 µL aliquot of C2Am in PBS buffer at pH 7.6 (*ca.* 0.5 mg/mL, 0.616 nmol) was transferred to a 0.5 mL eppendorf tube. Py-Tz **3** (200 eq, 3.1 µL of a stock solution of 0.4 mg in 34 µL DMF, 123.2 nmol) was added and the

resulting mixture vortexed for 30 seconds. After 96 h of additional shaking at 37 ºC, a 8 μL aliquot was analyzed by LC–MS and starting protein (calculated mass, 16222; observed mass, 16223) was detected unaltered.

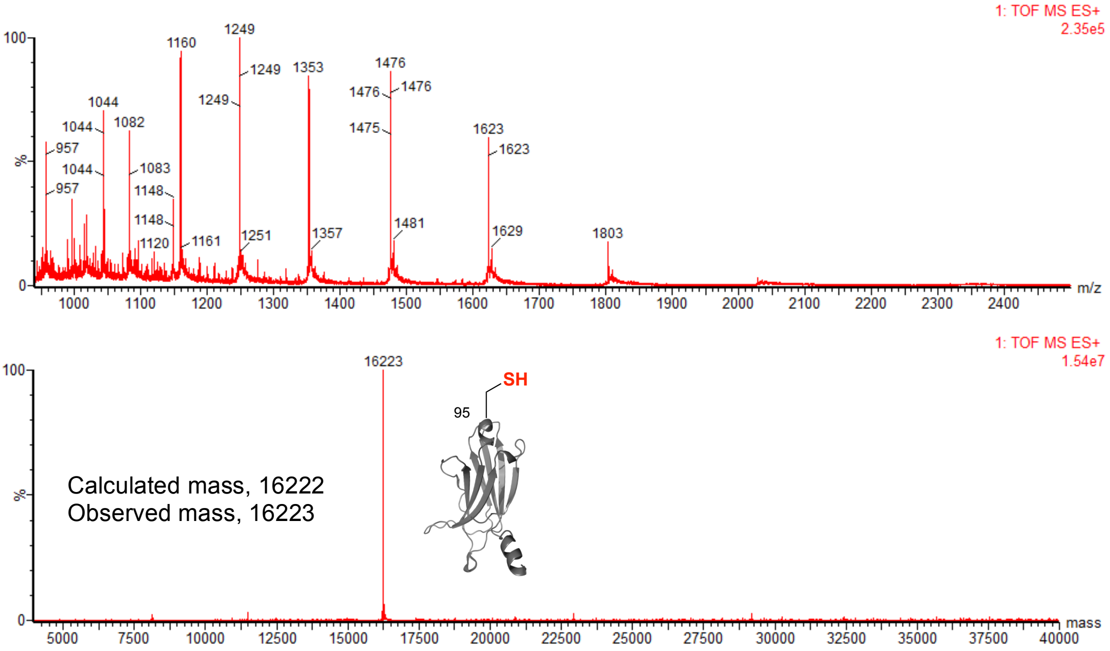

Figure S33 ESI–MS of the reaction of Py-Tz **2** with C2Am after 96 h at 37 °C.

2.6. LC–MS/MS analysis

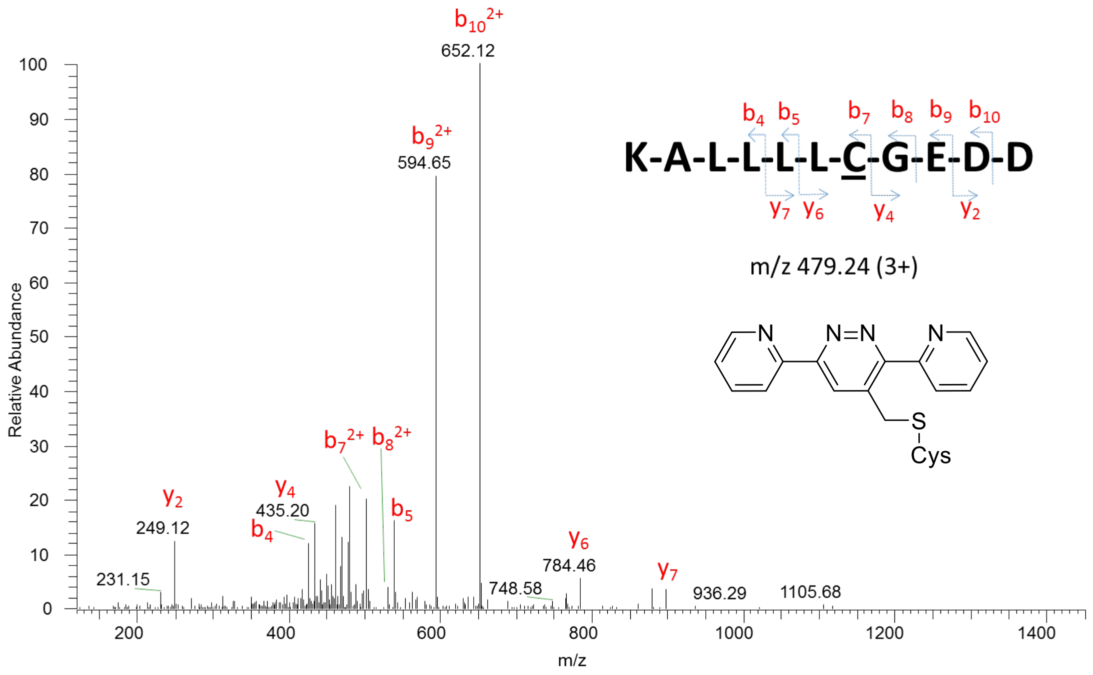

Figure S34 MS/MS spectrum of the *m/z* 479.24 (3+) ion of the tryptic peptide KALLLLCGEDD from AnxV, containing the S-allyl/PyTz **2** modification at the original cysteine residue. The generated fragment ions are consistent with the mass of the modification.

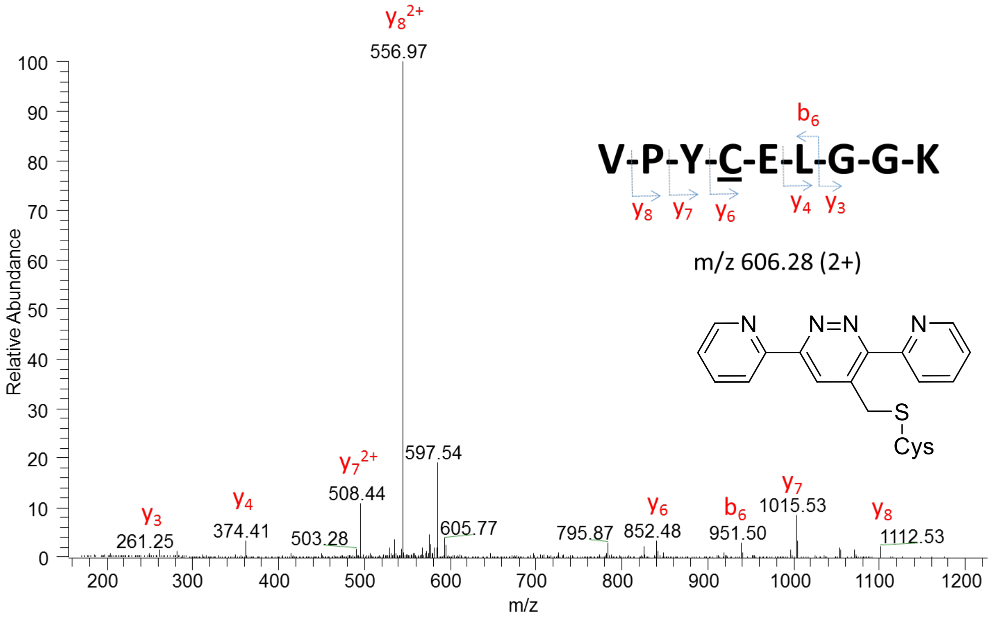

Figure S35 MS/MS spectrum of the *m/z* 606.28 (2+) ion of the tryptic peptide VPYC̲ELGGK from C2Am, containing the S-allyl/PyTz **2** modification at the original cysteine residue. The generated fragment ions are consistent with the mass of the modification.

2.7. NuPAGE Bis-Tris gels

AnxV S-allyl Cys was reacted with an excess of Tz-Rhod **4** and Tz-Cy3 **5** as previous described (**Sections 2.4.7.** and **2.4.8.**). After 12 h at 37 ºC, the reaction mixture was purified by dialysis for 5 h against PBS pH 7.6 using a Slide-A-Lyzer™ MINI dialysis device (Thermo Fisher Scientific) and analyzed by SDS-PAGE. The dialyzed solution was transferred to a tube (5.0 µL), and NuPAGE LDS sample buffer (4×, 2.5 µL), NuPAGE reducing agent (10×, 1 µL), and H_2O (1.5 µL) were added. The solution was denatured at 70 °C for 10 min. The heated solution was loaded onto a NuPAGE Bis-Tris mini gel (10 cm x 10 cm) with 4 – 12% gradient polyacrylamide concentration, and then subjected to electrophoresis (200 V, 35 min). The buffering system employed was 1× SDS running buffer (NuPAGE MES SDS running buffer pH 7.3). For the molecular weight marker, 5 µl of the Mark12 unstained standard (Thermo Fisher Scientific) was loaded on the gel. After electrophoresis the gel was fixed using a solution of 10 % acetic acid and 45% MeOH for 30 min. Fluorescent proteins were detected using a fluorescent imager (Typhoon 9210, Amersham Biosciences) followed by Coomassie stain using Instant Blue (Expedion).

2.8. Cell growth conditions and fluorescent microscopy

Human embryonic kidney HEK293 cells were maintained at 37 ºC in a humidified atmosphere of 5% CO_2 in DMEM high glucose (Gibco) supplemented with 10% heat inactivated fetal bovine serum (HI-FBS, Gibco) and 1× antibiotic-antimycotic (Gibco). When cells have reached the appropriate density (70% - 80% confluent), the medium was aspirated and cells harvested with 0.25% Trypsin-EDTA. Then, 200 µL of the cell suspension (~50 000 cells) was applied on top of 12 mm glass coverslips pre-coated with poly d-lysine (Corning BioCoat) placed inside a 24-well plate. After 1 h of incubation to allow cells to adhere, 200 µL of additional media was added to flood the wells. Cells were then grown for more 6 - 8 h at 37 ºC before apoptosis was induced by treatment for 12 h with 2 µM of actinomycin D in fresh growth media. Untreated cells at the same density were included as a control. After induction of apoptosis the media containing the cytotoxic drug was removed, the cells were washed 2 × with D-PBS and then preincubated with AnxV S-allyl Cys in 200 µL of fresh media for 45 min (4.5 µg, 0.125 nmol, 0.625 µM). Cells were then further washed 2 × before incubating for 1 h, 2 h and 3 h at 37 ºC with the tetrazine fluorophores diluted in 200 µL of fresh growth media (600 eqs, 75 nmol, 375 µM; stock solutions of Tz-Rhod **4** and Tz-Cy3 **5** prepared in DMF and H_2O, respectively). Blocking studies were performed by preincubating apoptotic cells for 30 min with a 10× excess of non-fluorescent AnxV (45 µg, 1.26 nmol, 6.3 µM) before incubation with AnxV S-allyl Cys for 45 min and reaction with Tz-Cy3 **5** for 3 h at 37 ºC. For fluorescent DNA nuclei staining, Hoechst 33342 (0.8 µg/mL, 1.3 µM, Sigma Aldrich) was incubated with the cells for 20 min. To test binding specificity cells were treated with Tz-Cy3 **5** for 3 h at 37 ºC without preincubation with AnxV S-allyl Cys. After labeling cells were washed in PBS two times and then fixed with PBS containing 4% (w/v) formaldehyde for 15 min at room temperature. Finally, cells were further washed two times with mili-Q water, and mounted on slides with Ibidi mounting medium.

Fluorescence microscopy was performed using an inverted epifluorescent microscope (Olympus IX-71) connected to a F-view digital camera (Soft Imaging System). Images were acquired in the Tritc and Hoechst channels and analyzed using the software Cell-F. Identical

image acquisition settings were used for the control, experimental and blocking data sets. Images were analysed using ImageJ (http://fiji.sc/wiki/index.php).

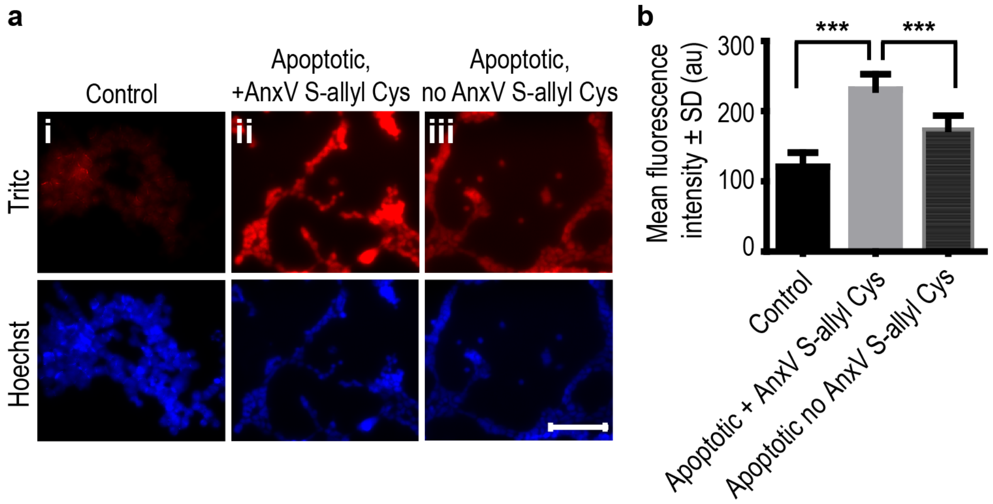

Figure S36 a. Fluorescent microscope images of HEK cells after pretargeting with AnxV S-allyl Cys and subsequent labeling with Tz-Rhod **4**. **b.** Quantification of mean fluorescence intensity of apoptotic and control cells. Non-apoptotic (**a i**) and apoptotic cells (**a ii**) were incubated with AnxV S-allyl Cys at 37 ºC for 45 min and then labeled with Tz-Rhod **4** for 3 h. After washing, the cells were imaged using both Tritc and Hoechst channels. Apoptotic cells are shown red, while the nuclei counterstained with Hoechst 33342 are shown blue. Significant labeling was observed for apoptotic cells when compared to non-apoptotic cells (**a**, i vs ii). To test binding specificity, apoptotic cells were treated only with Tz-Rhod **4** (**a**, iii). Apoptotic cells without being exposed to the targeting protein AnxV S-allyl Cys showed a predominant background indicating dye sticking. The mean fluorescence intensity in (**b**) was quantified in 30 predetermined regions of interest using ImageJ software. Data were statistically analyzed by means of a t-test (***p<0.001). Scale bar represents 100 µm; SD, standard deviation; au, arbitrary units.

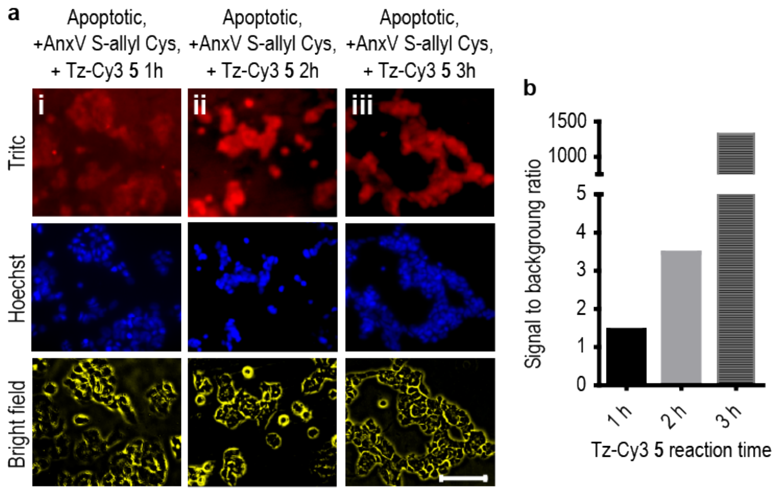

Figure S37 a. Fluorescent images of HEK cells pretargeted with AnxV S-allyl Cys and subsequent labeled with Tz-Cy3 **5** for 1, 2 and 3 h (i, ii, and iii, respectively). Apoptotic cells are shown red (tritc channel) and nuclei blue (Hoechst channel). **b**. Signal to background ratios calculated for cells labeled with Tz-Cy3 **5** for different reaction times (1 – 3 h). The mean fluorescence intensity of cells was quantified in predetermined regions of interest (ROI) using ImageJ software. Background quantification was performed by measuring the mean fluorescence intensity of regions drawn outside the cells close to the defined ROI. These results indicate that longer reaction times between the tetrazine and the pretargeted cells results in higher contrasts (**b**). Scale bar 100 µm.

2.9. Toxicity and stability of 1

Cell culture

HepG2 (a human hepatoma cell line) and HeLa cells (derived from cervical cancer cells) were maintained in a humidified incubator at 37 ºC under 5% CO2 and grown using 1x D-MEM (Dulbecco's modified Eagle medium) with Sodium Pyruvate and without L-Glutamine (Invitrogen, Life Technologies) supplemented with 10% heat-inactivated fetal bovine serum (FBS) (Gibco, Life Technologies), 1x MEM NEAA (Gibco, Life Technologies) 1x GlutaMAX (Gibco, Life Technologies), 200 units/mL penicillin and 200 µg/mL streptomycin (Gibco, Life Technologies) and 10 mM HEPES (Gibco, Life Technologies).

Cell viability assay

10 000 cells/well were seeded in 96 well-plates and were treated with S-Allyl Cys 24 h after the seeding, to allow the cells to stabilize. The cells were incubated with 1mM of the compound for 24 hours. After this incubation period, the culture medium was removed and the cells were incubated with CellTiter-Blue (Promega) for 1 h 30 min at 37 ºC. The cells viability was evaluated by measuring the Emission Intensity in RFUs – relative fluorescent Units- with an Infinite M200 plate reader. After the reading the cells were re-incubated with S-Allyl Cys for an additional period of 24 h, for a total incubation time of 48 h. The cells viability assay was done as mentioned for the 24 h time-point.

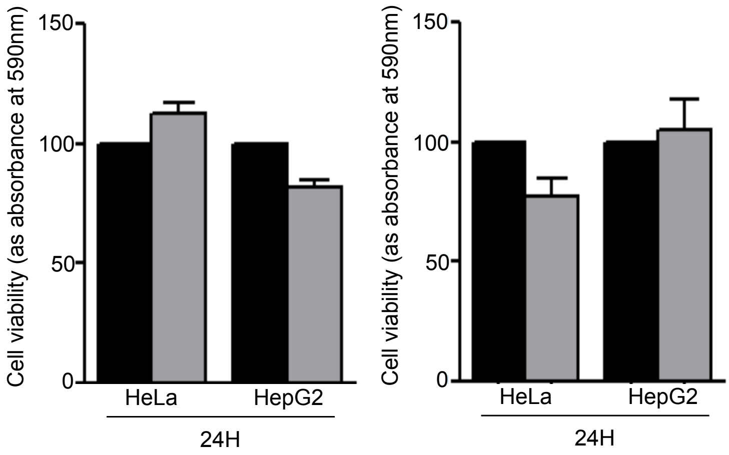

Figure S38 HeLa and HepG2 cells viability after treatment with 1mM of S-Allyl Cys for 24h and 48h and compared with the control treatment (medium + vehicle – PBS). The results correspond to 3 biological replicates (each with 6 technical replicates) and are shown as percentage of control (mean +sd).Differences were tested with a Mann-Whitney test that indicated no significant differences between S-Allyl Cys treatment and control.

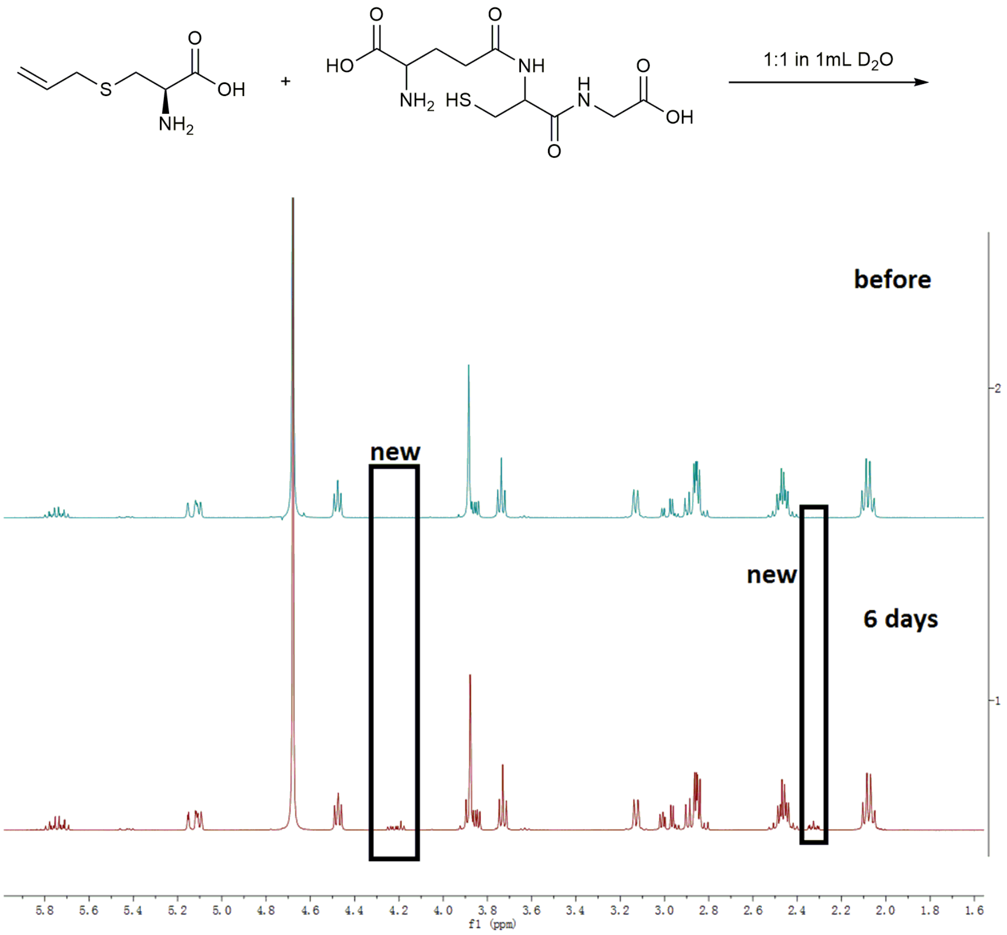

Figure S39 NMR analysis of the reaction of S-allyl Cys **1** in the presence of GSH for 6 days. No major degradation or products were observed indicating the stability of **1** towards biological thiol nucleophiles.

2.10. Computational Details

Full geometry optimizations were carried out with Gaussian 09[4] using the M06-2X hybrid functional[5] and 6-31G(d) basis set. Bulk solvent effects in water were considered implicitly through the IEF-PCM polarizable continuum model.[6] The possibility of different conformations was taken into account for all structures. Frequency analyses were carried out at the same level used in the geometry optimizations, and the nature of the stationary points was determined in each case according to the appropriate number of negative eigenvalues of the Hessian matrix. Scaled frequencies were not considered. Scaled frequencies were not considered. Mass-weighted intrinsic reaction coordinate (IRC) calculations were carried out by using the Gonzalez and Schlegel scheme[7,8] in order to ensure that the TSs indeed connected the appropriate reactants and products. Gibbs free energies (ΔG) were used for the discussion on the relative stabilities of the considered structures. Free energies calculated using the gas phase standard state concentration (1 atm = 1/24.5 M) were converted to reproduce the standard state concentration in solution (1 M) by adding or subtracting 1.89 kcal mol^{-1} for bimolecular additions and decompositions, respectively. Cartesian coordinates, electronic energies, entropies, enthalpies, Gibbs free energies, and lowest frequencies of the calculated structures are available below.

Discussion: To further analyze the reactivity of the dihydropyridazine species towards Py-Tz **2**, we carried out a detailed computational study of the whole reaction profile at the PCM(H_2O)/M06-2X/6-31G(p) level of theory using complete models for both the amino acid and tetrazine counterparts . We predict free energy barriers of 25.0 and 23.8 kcal mol^{-1} for the first and second IEDDA cycloaddition reactions, respectively. The first Py-Tz **2** cycloaddition is rate-limiting with an activation energy in good agreement with the experimentally determined moderate kinetics (calculated reaction time $1/k$ = 3.9 days). Of note, only one of the four calculated dihydropyridazine tautomers is reactive enough, the other three being either unable to add a second tetrazine unit or leading to too high activation barriers (*ca.* 31 kcal mol^{-1}). Since up to three of these tautomers are of similar energy and are expected to be in fast equilibrium, and given the similar activation energies

for the first and second tetrazine cycloadditions, it is expected that both unreacted dihydropyridazines and bis-adducts are observed in the reaction mixture.

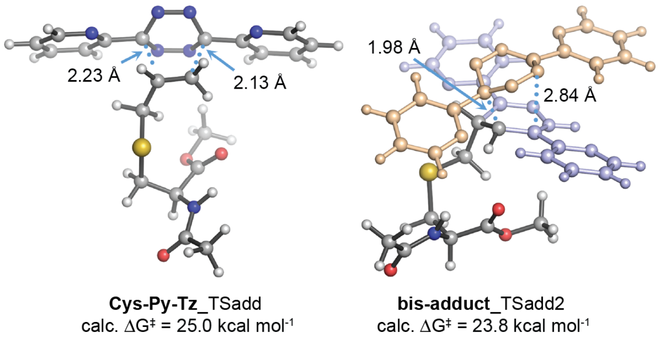

Figure S40 Transition structures (TS) calculated with PCM(H_2O)/M06-2X/6-31G(p) for the first and second cycloadditions of Py-Tz **2** to *S*-allyl Cys derivative

Activation energies ($\Delta G^{\ddagger}$, in kcal mol^{-1}) are calculated with respect to the separated reactants (for the first cycloaddition) and the lowest energy dihydropyridazine tautomer (for the second cycloaddition). DFT computations show that addition to **bis_adduct'_oxi-TSadd1** leads to too high activation barriers. Since the oxidation of the dihydropyridazines tautomers is proposed to be faster than the attack by a second tetrazine molecule the formation of bisproduct is very limited and always obtained in low yields. The competition between oxidation and second addition explains why the formation of the bis-adduct is observed from the beginning but only increases slightly over time. A way to favor the double cycloaddition would be the increase of the excess of tetrazine and/or the use of reducing conditions, or at least non-oxidizing conditions (Ar atmosphere and degassed solvents).

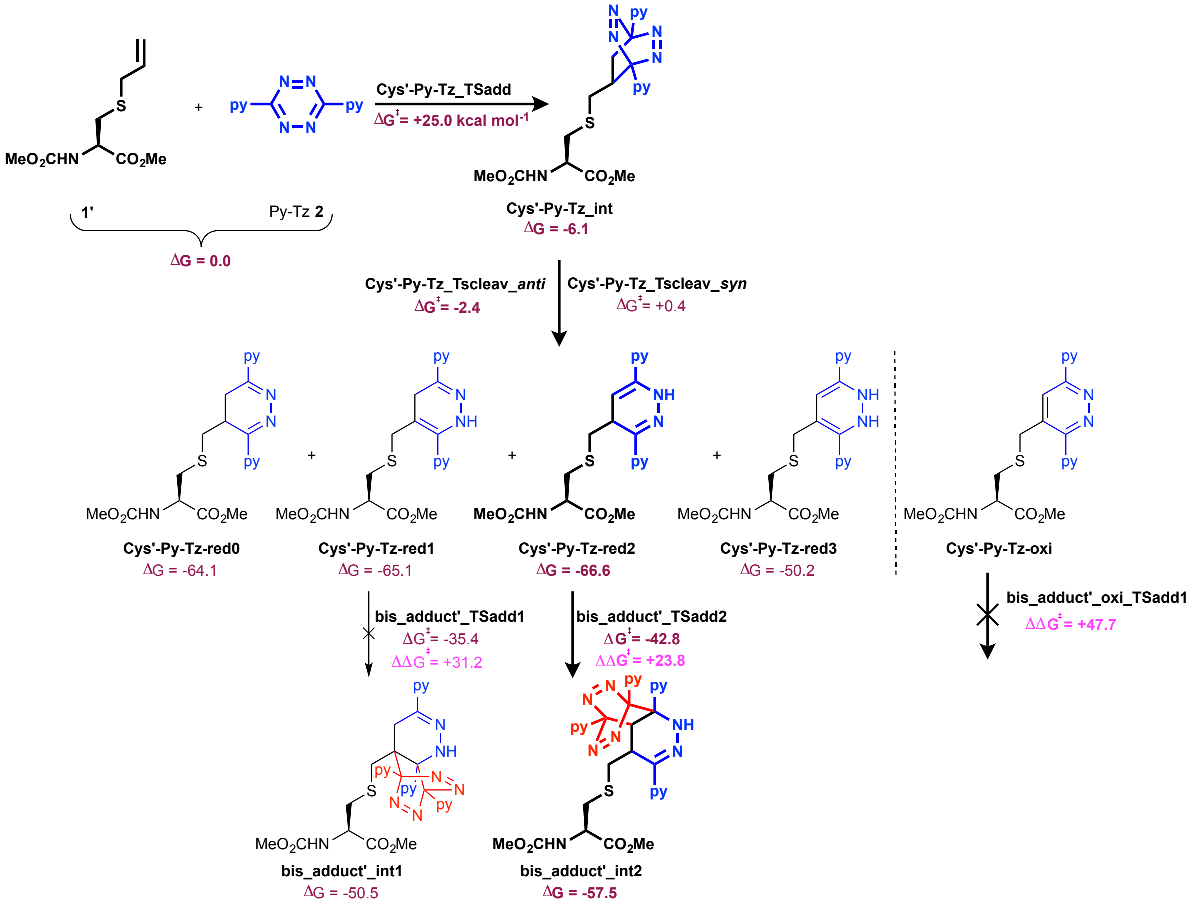

Figure S41 Activation (ΔG‡) and reaction (ΔG) free energies (in kcal mol^{-1}) calculated with PCM(H_2O)/M06-2X/6-31G(d) for the successive addition of di-pyridyltetrazine Py-Tz **2** to S-allylcysteine derivative **1'**. Four tautomers of the reduced dihydropyridazine (**Cys'-Py-Tz-red0** to **Cys'-Py-Tz-red4**) are shown. The structures involved in the minimum energy pathway for the formation of **bis_adduct'_int2** are shown with thicker lines.

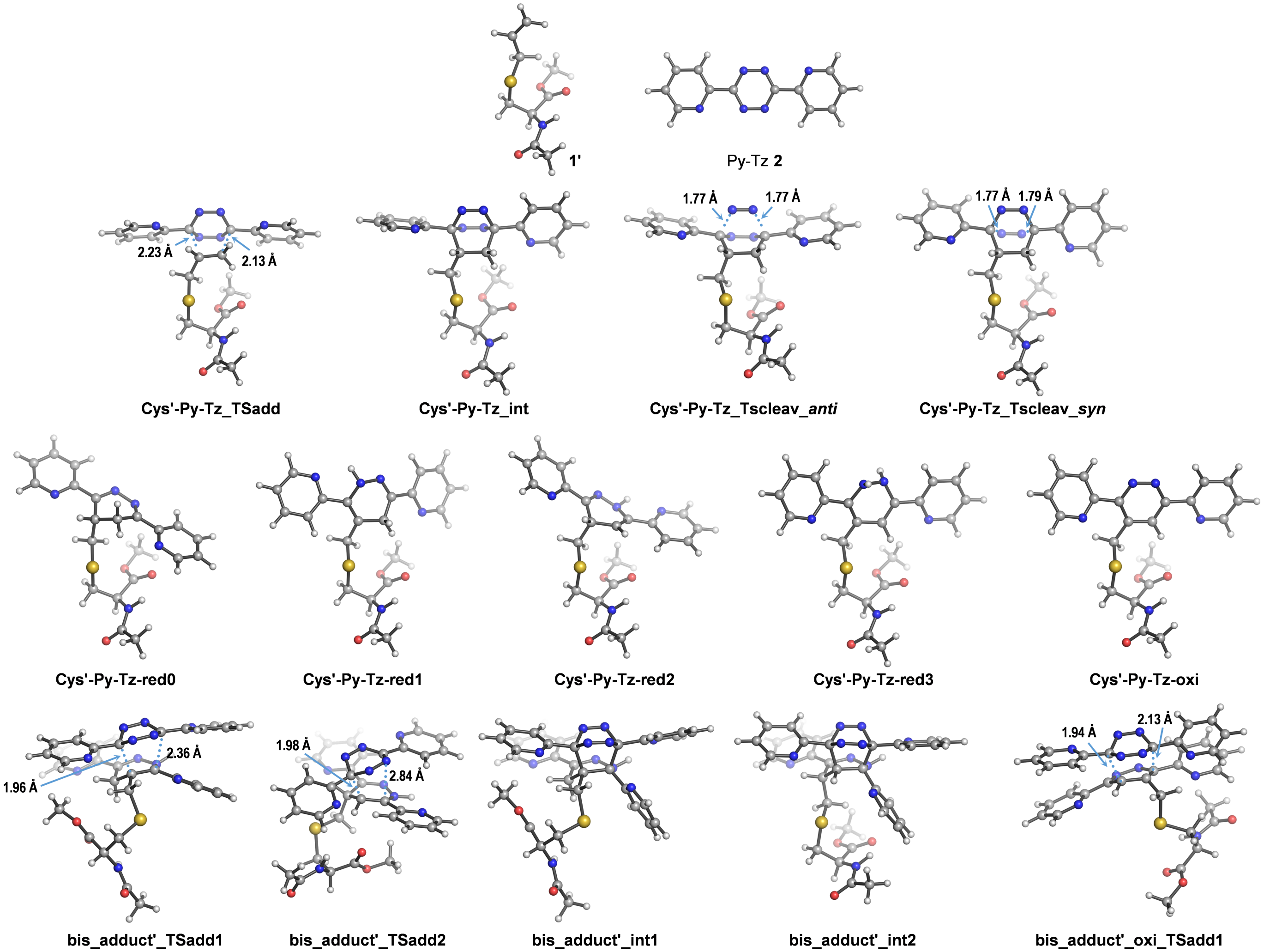

Figure S42 Guide to compound numbering of calculated structures (only the lowest energy conformers are shown).

Table S1: Energies, entropies, and lowest frequencies of the lowest energy calculated structures.[a]

Structure	E_{elec} (Hartree)	E_{elec} + ZPE (Hartree)	H (Hartree)	S (cal mol^{-1} K^{-1})	G (Hartree)	Lowest freq. (cm^{-1})	# of imag freq.
**N2**	–109.487797	–109.481998	–109.478693	45.8	–109.500435	2545.9	0
**1'**	–1030.307165	–1030.066581	–1030.048963	134.3	–1030.112782	33.0	0
**2**	–790.216712	–790.024546	–790.010545	119.2	–790.067190	22.7	0
**Cys'-Py-Tz_TSadd**	–1820.514587	–1820.079364	–1820.048908	198.7	–1820.143325	–377.9	1
**Cys'-Py-Tz_int**	–1820.568428	–1820.129627	–1820.099676	196.2	–1820.192908	22.7	0
**Cys'-Py-Tz_Tscleav_*anti***	–1820.554942	–1820.118717	–1820.088399	201.3	–1820.184025	–581.7	1
**Cys'-Py-Tz_Tscleav_*syn***	–1820.552950	–1820.116595	–1820.086468	196.1	–1820.179630	–588.3	1
**Cys'-Py-Tz-red0**	–1711.147724	–1710.719414	–1710.690492	192.3	–1710.781863	7.0	0
**Cys'-Py-Tz-red1**	–1711.149762	–1710.720849	–1710.691683	193.2	–1710.783498	21.8	0
**Cys'-Py-Tz-red2**	–1711.150142	–1710.721396	–1710.692140	197.4	–1710.785912	4.8	0
**Cys'-Py-Tz-red3**	–1711.125823	–1710.697161	–1710.667861	193.6	–1710.759836	23.1	0
**Cys'-Py-Tz-oxi**	–1709.956816	–1709.552304	–1709.523480	196.6	–1709.616890	7.4	0
**bis_adduct'_TSadd1**	–2501.350480	–2500.728371	–2500.686159	253.6	–2500.806631	–370.8	1
**bis_adduct'_TSadd2**	–2501.364994	–2500.741975	–2500.699994	249.3	–2500.818452	–420.4	1
**bis_adduct'_int1**	–2501.380636	–2500.755425	–2500.713966	245.8	–2500.830740	18.2	0
**bis_adduct'_int2**	–2501.391480	–2500.765512	–2500.724018	248.0	–2500.841859	19.9	0
**bis_adduct'_oxi_TSadd1**	–2500.134506	–2499.536423	-2499.495007	244.7	–2499.611269	–542.5	1

[a]Energy values calculated at the PCM(H_2O)/M06-2X/6-31G(d) level. 1 Hartree = 627.51 kcal mol^{-1}.
Thermal corrections at 298.15 K.

Cartesian coordinates of the lowest energy structures calculated with PCM(H_2O)/M06-2X/6-31G(d)

```
Structure N2
 N     0.00000   0.00000   0.54930
 N     0.00000   0.00000  -0.54930

Structure 1'
 N     1.92620   0.05760   0.49290
 H     1.62250   0.08350   1.45770
 C     1.05610   0.64830  -0.49140
 H     1.64990   1.28140  -1.16210
 C     0.34500  -0.39780  -1.36970
 C     0.05180   1.52750   0.22970
 H    -0.23410   0.09960  -2.15230
 H     1.12030  -1.00230  -1.84580
 S    -0.72090  -1.56010  -0.46540
 O    -0.06830   1.58480   1.43220
 C     3.11850  -0.47970   0.13980
 O     3.49820  -0.49520  -1.02780
 C     3.94110  -1.06630   1.26440
 H     3.51430  -0.87850   2.25080
 H     4.02350  -2.14520   1.11020
 H     4.94730  -0.64480   1.21730
 O    -0.70560   2.19720  -0.63450
 C    -1.71420   3.03730  -0.05620
 H    -2.20750   3.52310  -0.89470
 H    -2.42620   2.43310   0.51030
 H    -1.25370   3.77420   0.60320
 C    -2.25290  -0.57200  -0.23490
 H    -2.38260   0.05100  -1.12630
 H    -2.15260   0.07240   0.64290
 C    -3.40540  -1.51370  -0.06090
 H    -3.67280  -2.10890  -0.93220
 C    -4.07050  -1.65750   1.08230
 H    -3.81060  -1.07830   1.96500
 H    -4.89870  -2.35280   1.17370

Structure 2
 N    -0.64410  -1.19300  -0.03200
 C    -1.28480  -0.01490  -0.02010
 C     1.28480   0.01490  -0.02010
 N     0.66100  -1.17200  -0.02950
 N    -0.66100   1.17200  -0.02940
 N     0.64410   1.19300  -0.03190
 C    -2.77180  -0.00650  -0.00330
 C    -3.46280   1.20300  -0.09800
 N    -3.37670  -1.19510   0.10650
 C    -4.85310   1.17010  -0.07880
 H    -2.91940   2.13550  -0.18500
 C    -4.70780  -1.20790   0.12430
 C    -5.49310  -0.05800   0.03450
 H    -5.42350   2.09000  -0.15210
 H    -5.17540  -2.18500   0.21570
 H    -6.57440  -0.13280   0.05400
 C     2.77180   0.00650  -0.00330
 C     3.46280  -1.20300  -0.09800
 N     3.37670   1.19510   0.10650
 C     4.85310  -1.17010  -0.07880
 H     2.91940  -2.13560  -0.18490
 C     4.70780   1.20790   0.12430
 C     5.49310   0.05800   0.03450
 H     5.42350  -2.09000  -0.15210
 H     5.17540   2.18500   0.21560
 H     6.57440   0.13280   0.05400

Structure Cys'-Py-Tz_TSadd
 N    -4.33240  -0.15320  -0.33080
 H    -3.96270   0.64240  -0.83520
 C    -3.55250  -0.65770   0.77060
 H    -4.21920  -0.88880   1.60900
 C    -2.80030  -1.95460   0.41010
 C    -2.58540   0.42810   1.20200
 H    -2.25930  -2.34400   1.27650
 H    -3.54920  -2.69150   0.11290
 S    -1.65800  -1.75850  -0.99520
 O    -2.32420   1.40960   0.54260
 C    -5.49940  -0.74210  -0.68860
 O    -5.93800  -1.71900  -0.08850
 C    -6.22330  -0.12250  -1.86200
 H    -6.35400  -0.88530  -2.63300
 H    -7.21640   0.19180  -1.53230
 H    -5.69740   0.73340  -2.28750
 O    -2.02690   0.14040   2.37360
 C    -1.07400   1.09870   2.86110
 H    -0.75770   0.72680   3.83290
 H    -0.21990   1.16460   2.18200
 H    -1.54660   2.07770   2.95460
 C    -0.09870  -1.35700  -0.10650
 H     0.34850  -2.28640   0.25350
 H    -0.33360  -0.72820   0.75720
 C     0.80680  -0.60990  -1.04860
 H     1.29510  -1.18570  -1.83230
 C     0.65440   0.75340  -1.19370
 H    -0.13080   1.25690  -0.63060
 H     1.00890   1.26570  -2.08220
 C     2.73550  -0.77480   0.05350
 C     2.20640   1.65850  -0.05670
 N     2.30060  -0.19990   1.21540
 N     2.02950   1.05130   1.15930
 N     3.26320   1.22870  -0.83800
 N     3.51930  -0.01940  -0.79130
 C     1.81310   3.09220  -0.17260
 C     1.36490   3.80380   0.93980
 C     0.99840   5.13440   0.76220
 H     1.32000   3.32620   1.91120
 C     1.54650   4.89130  -1.55200
 C     1.09150   5.69390  -0.50680
 H     0.64900   5.72260   1.60420
 H     1.62540   5.29270  -2.55910
 H     0.81610   6.72600  -0.69130
 C     2.95110  -2.24650   0.03910
 C     2.90430  -2.98990   1.21840
 C     3.35920  -4.10150  -1.22780
 C     3.10610  -4.36420   1.13560
```

C	3.34130	-4.93490	-0.11000
H	3.53050	-4.51450	-2.21860
H	3.08130	-4.97630	2.03110
H	3.50250	-6.00090	-0.22200
N	3.16620	-2.78400	-1.16680
N	1.89850	3.61470	-1.40020
H	2.72000	-2.49570	2.16490

Structure **Cys'-Py-Tz_int**

N	-4.43150	-0.42320	-0.33020
H	-4.25850	0.49320	-0.72360
C	-3.57070	-0.85050	0.74580
H	-4.18600	-1.17400	1.59580
C	-2.68120	-2.03470	0.34000
C	-2.74520	0.34820	1.18520
H	-2.11930	-2.40860	1.19880
H	-3.33400	-2.83210	-0.01840
S	-1.51330	-1.60620	-0.99620
O	-2.83080	1.45050	0.69490
C	-5.52910	-1.14120	-0.67390
O	-5.79860	-2.20720	-0.12850
C	-6.39400	-0.54920	-1.76320
H	-5.96870	0.35320	-2.20510
H	-6.53750	-1.30100	-2.54220
H	-7.37440	-0.31450	-1.34100
O	-1.91210	0.02620	2.17060
C	-1.10630	1.10090	2.68690
H	-0.40140	0.63180	3.37050
H	-0.58110	1.61730	1.88000
H	-1.74470	1.80890	3.21920
C	0.04760	-1.50620	-0.04680
H	0.42770	-2.51660	0.12440
H	-0.15910	-1.03850	0.92030
C	1.05800	-0.67290	-0.83330
H	1.21620	-1.11830	-1.81940
C	0.67320	0.80280	-0.93760
H	-0.24760	1.03570	-0.39440
H	0.55100	1.13250	-1.97110
C	2.46180	-0.71840	-0.13250
C	1.82110	1.64140	-0.30460
N	2.30130	-0.04320	1.16300
N	1.97380	1.14640	1.08410
N	3.03710	1.29330	-1.05650
N	3.36110	0.10420	-0.98230
C	1.54120	3.12320	-0.30320
C	2.34780	4.03760	-0.97450
C	1.99760	5.38460	-0.92000
H	3.22000	3.69990	-1.52010
C	0.12830	4.76410	0.43190
C	0.86780	5.76000	-0.20320
H	2.60210	6.12730	-1.43030
H	-0.76330	5.01570	1.00080
H	0.55960	6.79710	-0.13480
C	3.02330	-2.11210	0.00780
C	3.42710	-2.63250	1.23420
C	3.58760	-4.02990	-1.09970
C	3.93690	-3.92850	1.26130
C	4.02140	-4.64440	0.07360
H	3.63550	-4.55890	-2.04830
H	4.26220	-4.36860	2.19840
H	4.41090	-5.65580	0.04870

N	3.09560	-2.79120	-1.14180
N	0.44970	3.47100	0.38560
H	3.34100	-2.03800	2.13520

Structure **Cys'-Py-Tz_Tscleav_*anti***

N	-4.19870	-0.88310	-0.18040
H	-3.98850	0.02360	-0.57770
C	-3.33720	-1.36020	0.87170
H	-3.95130	-1.76260	1.68640
C	-2.40730	-2.49220	0.39730
C	-2.54730	-0.17910	1.40530
H	-1.81310	-2.88020	1.22850
H	-3.04270	-3.29780	0.02470
S	-1.30120	-2.00180	-0.96600
O	-2.52840	0.91760	0.89250
C	-5.27230	-1.60400	-0.58600
O	-5.54070	-2.69410	-0.08970
C	-6.11650	-0.98160	-1.67450
H	-7.11460	-0.78810	-1.27350
H	-5.69930	-0.05070	-2.06150
H	-6.21850	-1.70000	-2.49070
O	-1.85330	-0.50950	2.49030
C	-1.06240	0.54330	3.06540
H	-0.56580	0.09900	3.92470
H	-0.32730	0.90520	2.34210
H	-1.70910	1.36630	3.37280
C	0.21810	-1.58230	-0.03580
H	0.77410	-2.50010	0.16780
H	-0.05910	-1.12500	0.91920
C	1.07060	-0.60380	-0.85330
H	1.28020	-1.04400	-1.83130
C	0.40790	0.77310	-0.98290
H	-0.58980	0.77730	-0.53300
H	0.29020	1.09130	-2.02000
C	2.41700	-0.43400	-0.12500
C	1.24060	1.81120	-0.23480
N	2.37250	0.30110	1.03970
N	1.76860	1.43610	0.98610
N	2.65660	1.72760	-1.29230
N	3.18080	0.68680	-1.25720
C	0.75420	3.22520	-0.27130
C	1.37440	4.20260	0.50900
C	0.90430	5.50760	0.42860
H	2.19850	3.93240	1.15940
C	-0.70270	4.74120	-1.16680
C	-0.15820	5.78660	-0.42610
H	1.36070	6.29400	1.02090
H	-1.53240	4.92190	-1.84570
H	-0.55760	6.78990	-0.52190
C	3.32390	-1.62610	-0.07400
C	4.29300	-1.74710	0.92200
C	3.98240	-3.57440	-1.07430
C	5.13210	-2.85550	0.89350
C	4.97790	-3.79070	-0.12450
H	3.83100	-4.28310	-1.88460
H	5.89580	-2.98460	1.65360
H	5.61040	-4.66900	-0.18630
N	3.16770	-2.51770	-1.05840
N	-0.26210	3.48250	-1.09980
H	4.37340	-0.99020	1.69260

Structure **Cys'-Py-Tz_Tscleav_*syn***
```
N    -4.50370  -0.37760  -0.32610
H    -4.33510   0.49630  -0.80830
C    -3.64040  -0.69240   0.78640
H    -4.25550  -0.93240   1.66390
C    -2.74630  -1.90700   0.50090
C    -2.82210   0.54790   1.10730
H    -2.17950  -2.18600   1.39200
H    -3.39600  -2.74060   0.23030
S    -1.58320  -1.61040  -0.87430
O    -2.91920   1.59980   0.51840
C    -5.60380  -1.12580  -0.58780
O    -5.86860  -2.13150   0.06390
C    -6.47970  -0.64360  -1.72160
H    -6.04360   0.18980  -2.27450
H    -6.65980  -1.47720  -2.40330
H    -7.44380  -0.33430  -1.30960
O    -1.98480   0.32550   2.11670
C    -1.19440   1.45270   2.53390
H    -0.49700   1.06080   3.27160
H    -0.66170   1.88800   1.68490
H    -1.84480   2.20640   2.98250
C    -0.01190  -1.45540   0.05130
H     0.33320  -2.44930   0.33580
H    -0.20120  -0.86660   0.95310
C     1.00620  -0.74690  -0.84870
H     1.01640  -1.24230  -1.82500
C     0.68370   0.74700  -1.00690
H    -0.21350   1.03290  -0.45070
H     0.51260   1.01230  -2.05360
C     2.45530  -0.86970  -0.34270
C     1.86580   1.59450  -0.53620
N     2.21290  -0.10760   1.24140
N     1.92750   1.01800   1.15690
N     3.10150   1.21950  -0.99620
N     3.39750  -0.03110  -0.91030
C     1.63780   3.07110  -0.44520
C     2.62480   3.98710  -0.80690
C     2.34200   5.34300  -0.67960
H     3.57870   3.63390  -1.17860
C     0.18510   4.73580   0.15030
C     1.09890   5.73030  -0.19020
H     3.08340   6.08530  -0.95740
H    -0.79630   4.99590   0.53920
H     0.83770   6.77590  -0.07280
C     3.00710  -2.24280  -0.10650
C     4.16330  -2.39110   0.65930
C     2.88740  -4.48600  -0.52060
C     4.68440  -3.66820   0.82950
C     4.03450  -4.74070   0.22630
H     2.35520  -5.29920  -1.00740
H     5.58050  -3.82350   1.42130
H     4.40310  -5.75490   0.33050
N     2.37350  -3.26460  -0.68830
N     0.43920   3.43270   0.02460
H     4.63390  -1.52010   1.10420
```

Structure **Cys'-Py-Tz-red0**
```
N    -3.49800  -1.47830   0.04120
H    -3.56110  -0.54140  -0.33740
C    -2.49260  -1.71310   1.04790
H    -2.94810  -2.25000   1.89030
C    -1.33400  -2.58160   0.53080
C    -2.02360  -0.36000   1.55640
H    -0.61530  -2.77980   1.32970
H    -1.76100  -3.53300   0.20790
S    -0.46640  -1.86550  -0.90270
O    -2.44070   0.69980   1.15290
C    -4.37030  -2.44910  -0.32060
O    -4.32750  -3.57260   0.17290
C    -5.39100  -2.06610  -1.36810
H    -5.30100  -1.02910  -1.69580
H    -5.27140  -2.72710  -2.22970
H    -6.39030  -2.23020  -0.95830
O    -1.09600  -0.48880   2.50350
C    -0.58730   0.74570   3.03450
H     0.13520   0.45980   3.79560
H    -0.10580   1.32600   2.24210
H    -1.40150   1.32590   3.47160
C     0.96060  -1.08510  -0.06860
H     1.60830  -1.86610   0.33940
H     0.60090  -0.46940   0.76060
C     1.76710  -0.22890  -1.05580
H     2.21300  -0.88040  -1.80950
C     0.96930   0.89190  -1.71880
H     0.06770   0.53450  -2.21490
H     1.59330   1.39100  -2.47220
C     2.87310   0.45550  -0.28520
C     0.60620   1.87520  -0.64410
N     1.41320   2.14040   0.32350
N     2.69660   1.58910   0.29620
C    -0.73570   2.51350  -0.62100
C    -1.02870   3.52890   0.29490
C    -2.30910   4.06350   0.30410
H    -0.25880   3.87570   0.97400
C    -2.85190   2.57650  -1.48730
C    -3.24690   3.57590  -0.60170
H    -2.57160   4.85060   1.00370
H    -3.55480   2.17910  -2.21600
H    -4.25940   3.96310  -0.63100
C     4.21140  -0.18380  -0.18090
C     5.26550   0.45100   0.48400
C     5.50830  -2.00960  -0.65840
C     6.49050  -0.19780   0.55390
C     6.61960  -1.45650  -0.02770
H     5.56990  -2.99180  -1.12020
H     7.33120   0.26870   1.05710
H     7.55650  -2.00100   0.00490
N     4.32760  -1.39390  -0.74260
N    -1.62650   2.05170  -1.50710
H     5.10670   1.42600   0.92860
```

Structure **Cys'-Py-Tz-red1**
```
N     4.27870  -0.10140  -0.57000
H     3.88470  -0.72570  -1.26240
C     3.62500  -0.04220   0.71410
H     4.37850  -0.13570   1.50610
C     2.89080   1.29150   0.93140
C     2.68000  -1.22760   0.81440
H     2.47140   1.34190   1.93930
H     3.62600   2.09100   0.82480
S     1.56420   1.58050  -0.28670
```

```
O     2.45650  -2.00060  -0.08890
C     5.43330   0.57080  -0.79760
O     5.94390   1.28400   0.06120
C     6.05200   0.38280  -2.16390
H     5.42750  -0.20320  -2.83980
H     6.23710   1.36580  -2.60190
H     7.01690  -0.11640  -2.04400
O     2.12080  -1.29940   2.01950
C     1.23600  -2.41370   2.23010
H     0.84780  -2.28840   3.23840
H     0.42750  -2.41020   1.49500
H     1.79670  -3.34670   2.14670
C     0.07890   1.11810   0.70450
H    -0.20480   1.95650   1.34340
H     0.37020   0.28020   1.34620
C    -1.02360   0.67920  -0.19860
C    -0.91170  -0.67420  -0.85610
H    -0.13080  -1.28860  -0.40500
H    -0.66400  -0.56790  -1.92400
C    -2.16060   1.35590  -0.45800
C    -2.26070  -1.33960  -0.74250
N    -3.34290  -0.67120  -0.92070
N    -3.19170   0.67480  -1.12570
C    -2.37490  -2.78840  -0.44380
C    -3.61580  -3.43900  -0.47200
C    -3.66380  -4.79440  -0.18440
H    -4.50900  -2.87670  -0.71670
C    -1.29910  -4.73040   0.13680
C    -2.48110  -5.46200   0.13100
H    -4.61050  -5.32520  -0.20250
H    -0.35390  -5.21050   0.37990
H    -2.47280  -6.52030   0.36580
C    -2.50010   2.75070  -0.08180
C    -1.52630   3.75130   0.00270
C    -4.16840   4.23820   0.44410
C    -1.91960   5.03560   0.36040
C    -3.26710   5.28890   0.59490
H    -5.23300   4.39970   0.59330
H    -1.18440   5.82990   0.43770
H    -3.61880   6.27670   0.86980
N    -3.80400   2.99860   0.11550
N    -1.23540  -3.42670  -0.14810
H    -0.48960   3.53330  -0.23370
H    -4.08320   1.15470  -1.05230
```

Structure **Cys'-Py-Tz-red2**

```
N    -3.69100  -1.61770  -0.23320
H    -3.60510  -0.80710  -0.83280
C    -2.86270  -1.65480   0.94630
H    -3.47330  -1.96270   1.80370
C    -1.70750  -2.66320   0.81770
C    -2.35100  -0.24890   1.20520
H    -1.13430  -2.71430   1.74680
H    -2.15190  -3.64340   0.63450
S    -0.58680  -2.30130  -0.57150
O    -2.47880   0.67660   0.43800
C    -4.57740  -2.60690  -0.49860
O    -4.69990  -3.57940   0.24080
C    -5.39620  -2.44380  -1.75910
H    -5.21420  -1.49690  -2.26940
H    -5.16070  -3.26780  -2.43720
H    -6.45450  -2.51800  -1.49940
O    -1.73480  -0.17270   2.38280
C    -1.21710   1.12060   2.73240
H    -0.85660   1.02340   3.75420
H    -0.39850   1.39130   2.06160
H    -2.00960   1.86800   2.67090
C     0.76460  -1.40600   0.27760
H     1.47540  -2.13020   0.68550
H     0.34830  -0.81440   1.09940
C     1.47520  -0.47270  -0.72230
H     1.76210  -1.06550  -1.59570
C     0.61730   0.69840  -1.11670
H    -0.04290   0.62490  -1.97180
C     2.73290   0.07310  -0.07600
C     0.66470   1.80730  -0.36330
N     2.75740   1.16660   0.60090
N     1.61360   1.89490   0.65980
C    -0.18850   3.01080  -0.51430
C    -1.34710   2.99780  -1.29660
C    -2.08250   4.16890  -1.41170
H    -1.67490   2.08370  -1.77660
C    -0.50920   5.21340   0.04990
C    -1.65770   5.30630  -0.72830
H    -2.98350   4.19130  -2.01620
H    -0.15160   6.07170   0.61290
H    -2.20670   6.23920  -0.78580
C     3.99160  -0.70930  -0.15920
C     5.20130  -0.20040   0.33170
C     5.00150  -2.66460  -0.80560
C     6.34040  -0.98550   0.23810
C     6.24540  -2.24850  -0.34420
H     4.88510  -3.64270  -1.26640
H     7.29180  -0.61760   0.60930
H     7.11060  -2.89460  -0.44050
N     3.89410  -1.92260  -0.71920
N     0.21600   4.09760   0.15680
H     5.22700   0.78990   0.77040
H     1.75680   2.83290   1.01840
```

Structure **Cys'-Py-Tz-red3**

```
N    -4.13880  -0.26540  -0.65700
H    -3.78880   0.45970  -1.27030
C    -3.57080  -0.33980   0.66760
H    -4.38420  -0.35680   1.40520
C    -2.73470  -1.61100   0.87050
C    -2.75870   0.92450   0.89770
H    -2.40030  -1.68900   1.90750
H    -3.37960  -2.46450   0.65450
S    -1.28360  -1.69320  -0.23370
O    -2.65670   1.82310   0.09390
C    -5.20860  -1.02260  -1.00160
O    -5.69910  -1.83790  -0.22590
C    -5.75330  -0.80620  -2.39520
H    -5.19770  -0.05720  -2.96140
H    -5.72630  -1.75700  -2.93230
H    -6.79840  -0.49730  -2.31670
O    -2.18290   0.92060   2.09630
C    -1.46460   2.11640   2.45060
H    -1.02040   1.90810   3.42160
H    -0.69700   2.34350   1.70750
H    -2.16370   2.95210   2.52080
```

C	0.07750	-1.32940	0.95030
H	0.42150	-2.26370	1.38900
H	-0.34410	-0.68720	1.72950
C	1.18750	-0.59920	0.25160
C	0.97270	0.81530	-0.01530
H	-0.04010	1.20620	-0.05220
C	2.38390	-1.13100	-0.10260
C	2.02430	1.63690	-0.19370
N	3.33430	1.08750	-0.23380
N	3.40530	-0.27150	-0.59710
C	1.92080	3.10310	-0.32900
C	3.01330	3.86410	-0.75790
C	2.87250	5.24490	-0.85040
H	3.94110	3.36950	-1.01950
C	0.61920	4.97830	-0.10790
C	1.65120	5.82120	-0.52160
H	3.70450	5.85910	-1.18010
H	-0.35120	5.39110	0.15940
H	1.49410	6.89230	-0.57880
C	2.76980	-2.55840	-0.05830
C	4.12060	-2.91840	0.03840
C	2.13110	-4.76130	-0.13740
C	4.45840	-4.26650	0.04650
C	3.44550	-5.21510	-0.04180
H	1.31110	-5.47190	-0.21450
H	5.49770	-4.57020	0.12380
H	3.66000	-6.27780	-0.04170
N	1.78990	-3.47380	-0.15530
N	0.73990	3.65740	-0.00370
H	4.87480	-2.14470	0.11150
H	3.53060	-0.35070	-1.60500
H	3.84540	1.23460	0.63590

Structure **Cys'-Py-Tz-oxi**

N	3.93850	0.17610	-0.67180
H	3.47360	-0.64090	-1.04640
C	3.50630	0.65060	0.61820
H	4.38580	0.89440	1.22560
C	2.65220	1.92870	0.51620
C	2.74280	-0.46710	1.30130
H	2.39450	2.30260	1.51050
H	3.26330	2.68350	0.01700
S	1.13750	1.73720	-0.47830
O	2.37920	-1.48090	0.74900
C	4.94360	0.79120	-1.34130
O	5.51260	1.77800	-0.88380
C	5.32020	0.18670	-2.67450
H	4.69560	-0.66360	-2.95210
H	5.24120	0.95930	-3.44260
H	6.36410	-0.13310	-2.62930
O	2.48730	-0.17420	2.57400
C	1.74530	-1.16500	3.30260
H	1.65600	-0.77790	4.31460
H	0.75730	-1.30050	2.85580
H	2.28300	-2.11380	3.29390
C	-0.08040	1.33830	0.83780
H	-0.47500	2.26590	1.24910
H	0.44610	0.79540	1.62660
C	-1.17780	0.45790	0.30090
C	-0.96440	-0.90440	0.20060
H	-0.00480	-1.34880	0.44970
C	-2.46100	0.91010	-0.07670
C	-2.01430	-1.72460	-0.22060
N	-3.42770	0.06720	-0.45750
N	-3.21710	-1.23010	-0.52260
C	-1.84640	-3.19950	-0.33290
C	-2.88450	-4.00840	-0.80120
C	-2.67020	-5.37900	-0.88460
H	-3.82730	-3.55990	-1.08860
C	-0.46470	-5.00230	-0.04700
C	-1.43580	-5.89260	-0.50060
H	-3.45580	-6.03530	-1.24500
H	0.51240	-5.36530	0.26230
H	-1.22400	-6.95470	-0.54920
C	-2.86570	2.34340	-0.08970
C	-4.18560	2.70410	0.19620
C	-2.27080	4.52190	-0.46770
C	-4.53230	4.04840	0.14180
C	-3.55730	4.98130	-0.19850
H	-1.48220	5.21890	-0.74020
H	-5.54710	4.36280	0.36300
H	-3.78280	6.04030	-0.25480
N	-1.92290	3.23480	-0.41770
N	-0.65540	-3.68600	0.03860
H	-4.90900	1.93980	0.45300

Structure **bis_adduct'_TSadd1**

N	3.41720	3.60550	-0.94550
H	3.35120	3.12640	-1.83490
C	3.53030	2.78530	0.23690
H	4.41080	3.10550	0.81110
C	2.31220	2.91940	1.15670
C	3.77740	1.35240	-0.20540
H	2.44540	2.32370	2.06200
H	2.23210	3.96890	1.44470
S	0.75530	2.43730	0.33460
O	3.93570	1.01380	-1.35600
C	3.61890	4.94540	-0.88500
O	3.82650	5.51830	0.18020
C	3.58250	5.68000	-2.20580
H	4.59690	6.00470	-2.45310
H	3.19630	5.06820	-3.02260
H	2.96310	6.57170	-2.09380
O	3.83160	0.52650	0.83590
C	4.28080	-0.81010	0.54680
H	4.19290	-1.35350	1.48550
H	3.66520	-1.27230	-0.22740
H	5.32250	-0.77540	0.22030
C	0.30640	0.91100	1.26230
H	-0.39860	1.16590	2.04910
H	1.22390	0.51970	1.70490
C	-0.26050	-0.15010	0.32680
C	0.83620	-0.73750	-0.55950
H	1.60670	-1.21170	0.04830
H	1.32750	0.08700	-1.10020
C	-1.45120	0.14630	-0.42820
C	0.28330	-1.71480	-1.55380
N	-1.62220	-0.50300	-1.62910
N	-0.89200	-1.58890	-2.04570
C	1.10480	-2.86600	-1.99560
C	0.57390	-3.87180	-2.81030
C	1.39410	-4.93060	-3.17350

H	-0.45800	-3.81120	-3.13560
C	3.14590	-3.90650	-1.91010
C	2.70970	-4.95460	-2.71510
H	1.01250	-5.72930	-3.80120
H	4.16500	-3.88650	-1.53110
H	3.38280	-5.76500	-2.97080
C	-2.25480	1.40380	-0.29670
C	-2.87200	1.96140	-1.41970
C	-3.08360	3.04640	1.06570
C	-3.64840	3.10540	-1.25120
C	-3.76160	3.66400	0.01370
H	-3.13650	3.45670	2.07110
H	-4.14810	3.55190	-2.10460
H	-4.35320	4.55480	0.19050
N	-2.35780	1.94300	0.92360
N	2.36870	-2.88240	-1.55300
H	-2.74030	1.53100	-2.40500
H	-2.59090	-0.58080	-1.94260
C	-3.02430	-1.05060	0.86080
C	-0.69810	-1.60140	1.57560
C	-4.28600	-0.55240	0.28240
C	-5.12590	0.29980	1.00550
C	-6.25180	0.81060	0.37440
H	-4.88090	0.55050	2.03070
C	-5.60450	-0.38820	-1.58790
C	-6.50550	0.45700	-0.94880
H	-6.92230	1.47740	0.90710
H	-5.75730	-0.67450	-2.62530
H	-7.37330	0.83250	-1.47890
C	0.61200	-2.10630	2.12510
C	1.20440	-3.25500	1.59310
C	2.28610	-1.86690	3.66660
C	2.39210	-3.71140	2.15370
H	0.73290	-3.77600	0.76850
C	2.94320	-3.01280	3.22280
H	2.69360	-1.28340	4.48860
H	2.87480	-4.60060	1.76080
H	3.86380	-3.33440	3.69700
N	-1.36140	-2.56310	0.79250
N	-2.52420	-2.24710	0.39670
N	-2.67790	-0.66440	2.12590
N	-1.51940	-0.98920	2.54340
N	-4.51190	-0.88350	-0.99950
N	1.14790	-1.41500	3.13630

Structure **bis_adduct'_TSadd2**

N	-4.74920	-0.13560	-0.67320
H	-4.26370	-0.63230	0.06360
C	-4.02630	0.08900	-1.90000
H	-4.70110	-0.06740	-2.74890
C	-3.47900	1.52570	-1.99420
C	-2.90150	-0.92570	-1.97570
H	-2.97590	1.69070	-2.95020
H	-4.33320	2.20290	-1.93480
S	-2.35550	1.94700	-0.62340
O	-2.54360	-1.61320	-1.04650
C	-5.99230	0.37390	-0.49190
O	-6.56160	1.01230	-1.37180
C	-6.61750	0.12730	0.86200
H	-7.66720	-0.13560	0.72110
H	-6.11730	-0.66310	1.42480
H	-6.57720	1.05530	1.43980
O	-2.33610	-0.93590	-3.18030
C	-1.23420	-1.84060	-3.35030
H	-1.05890	-1.89310	-4.42230
H	-0.34910	-1.44810	-2.84300
H	-1.48400	-2.82200	-2.94440
C	-0.72480	1.64230	-1.39490
H	-0.40500	2.54500	-1.92060
H	-0.80800	0.82690	-2.12140
C	0.29210	1.28070	-0.29540
H	0.17850	2.00480	0.52060
C	0.09910	-0.13280	0.22470
H	-0.94290	-0.43240	0.32210
C	1.69180	1.44950	-0.85050
C	0.86790	-1.10840	-0.45290
N	2.37220	0.49810	-1.38600
N	1.84140	-0.75100	-1.30410
C	0.61460	-2.56340	-0.37620
C	-0.37410	-3.09000	0.45510
C	-0.53960	-4.47250	0.46980
H	-0.99030	-2.44330	1.07280
C	1.23130	-4.63630	-1.13370
C	0.27090	-5.26210	-0.33620
H	-1.29570	-4.92360	1.10360
H	1.88370	-5.22020	-1.77710
H	0.16920	-6.34120	-0.35450
C	2.30610	2.79920	-0.85660
C	3.65570	2.97360	-1.18180
C	2.01390	5.04230	-0.50670
C	4.17620	4.26020	-1.16460
C	3.34090	5.32100	-0.82290
H	1.33370	5.84400	-0.23040
H	5.21950	4.43370	-1.40810
H	3.70610	6.34140	-0.79490
N	1.49810	3.81180	-0.51730
N	1.40580	-3.31900	-1.15560
H	4.26430	2.11030	-1.42310
H	2.40620	-1.49250	-1.71040
C	2.68030	-0.54610	1.66230
C	0.30030	-0.03870	2.19060
C	3.91640	-0.87920	0.93710
C	4.13470	-2.19730	0.52140
C	5.27030	-2.47800	-0.23130
H	3.42280	-2.97170	0.78570
C	5.83290	-0.16010	-0.07780
C	6.14170	-1.44020	-0.54060
H	5.46530	-3.48940	-0.57300
H	6.49350	0.67500	-0.30140
H	7.03720	-1.60810	-1.12820
C	-1.10790	0.28420	2.59350
C	-1.45780	1.55210	3.05520
C	-2.79010	1.79290	3.38050
H	-0.69790	2.31690	3.15920
C	-3.26090	-0.46860	2.77310
C	-3.71430	0.76410	3.24160
H	-3.09790	2.76910	3.74100
H	-3.94880	-1.30490	2.66210
H	-4.76010	0.90340	3.49240
N	-1.99200	-0.70890	2.44430
N	4.74860	0.12920	0.63780
N	0.76900	-1.28020	2.62670

```
N     1.97130  -1.54230   2.25790
N     1.21190   1.00100   2.38800
N     2.41060   0.73730   2.00220
```

Structure **bis_adduct'_int1**

```
N     3.61680   3.21280  -0.86070
H     3.49950   2.71350  -1.73340
C     3.62730   2.42870   0.35070
H     4.51940   2.68990   0.93590
C     2.40710   2.70680   1.23660
C     3.75590   0.96470  -0.03560
H     2.45660   2.12250   2.15770
H     2.43250   3.76520   1.50110
S     0.82300   2.39180   0.38410
O     3.85910   0.56690  -1.17290
C     3.92480   4.53290  -0.83940
O     4.16750   5.12100   0.21030
C     3.92960   5.23550  -2.17780
H     4.87360   5.77360  -2.28580
H     3.80060   4.55440  -3.02020
H     3.12220   5.97220  -2.18770
O     3.78080   0.18200   1.04070
C     4.14530  -1.19030   0.80430
H     4.23370  -1.63810   1.79120
H     3.38430  -1.70310   0.21030
H     5.10140  -1.22540   0.27810
C     0.22170   0.91220   1.29020
H    -0.50220   1.24540   2.03280
H     1.08020   0.48430   1.81150
C    -0.38630  -0.20830   0.41070
C     0.64590  -0.64130  -0.63950
H     1.54630  -1.01100  -0.14860
H     0.93020   0.25520  -1.20660
C    -1.74280   0.13520  -0.27110
C     0.08970  -1.63930  -1.59950
N    -1.78170  -0.37420  -1.65930
N    -1.08930  -1.47800  -2.07680
C     0.88300  -2.80870  -2.04320
C     0.33360  -3.78230  -2.88700
C     1.12060  -4.86310  -3.25600
H    -0.68920  -3.67790  -3.22940
C     2.88030  -3.93420  -1.93620
C     2.42500  -4.94860  -2.77150
H     0.72170  -5.63370  -3.90800
H     3.89040  -3.96130  -1.53290
H     3.07210  -5.77910  -3.02970
C    -2.07340   1.62820  -0.31480
C    -1.96290   2.36680  -1.49140
C    -2.75250   3.46450   0.87330
C    -2.26510   3.72570  -1.44830
C    -2.66360   4.29210  -0.24450
H    -3.06930   3.86410   1.83340
H    -2.18740   4.32980  -2.34650
H    -2.90870   5.34530  -0.16630
N    -2.47140   2.16280   0.84290
N     2.13630  -2.88550  -1.57670
H    -1.64100   1.88630  -2.40640
H    -2.75290  -0.46360  -1.95790
C    -2.88100  -0.59570   0.59030
C    -0.71520  -1.41990   1.40620
C    -4.29210  -0.21340   0.20410
C    -5.17740   0.35470   1.11790
C    -6.46040   0.67040   0.68300
H    -4.85870   0.54350   2.13460
C    -5.85000  -0.13980  -1.47800
C    -6.81010   0.41720  -0.63900
H    -7.17600   1.11120   1.36950
H    -6.07570  -0.34480  -2.52090
H    -7.79910   0.64810  -1.01780
C     0.48860  -2.10980   2.01840
C     0.96890  -3.30730   1.48650
C     2.08130  -2.10910   3.66210
C     2.05700  -3.91740   2.10300
H     0.49200  -3.75470   0.62310
C     2.61930  -3.31710   3.22340
H     2.50410  -1.59820   4.52360
H     2.45230  -4.85000   1.71320
H     3.46170  -3.76120   3.74210
N    -1.53030  -2.42660   0.68000
N    -2.63160  -2.03390   0.29700
N    -2.65810  -0.40830   2.02790
N    -1.58150  -0.84330   2.45280
N    -4.61810  -0.45390  -1.07090
N     1.04100  -1.51040   3.07840
```

Structure **bis_adduct'_int2**

```
N    -4.15790   2.27620   0.25320
H    -4.12780   1.31610  -0.06990
C    -3.18790   3.19070  -0.29010
H    -3.68000   4.14960  -0.49760
C    -2.02900   3.49050   0.67930
C    -2.69330   2.62940  -1.61230
H    -1.34430   4.21750   0.23550
H    -2.46550   3.92900   1.57940
S    -1.10080   2.03410   1.24410
O    -3.06010   1.58050  -2.09100
C    -4.99880   2.65380   1.24820
O    -5.00790   3.80050   1.68430
C    -5.89430   1.55880   1.78340
H    -5.31140   0.89910   2.43440
H    -6.69850   2.00930   2.36430
H    -6.31530   0.95540   0.97520
O    -1.78340   3.42730  -2.16550
C    -1.19830   2.94750  -3.38390
H    -0.51040   3.72580  -3.70550
H    -0.66040   2.01380  -3.19670
H    -1.97340   2.77960  -4.13270
C    -0.07240   1.69460  -0.22920
H     0.26510   2.65010  -0.64270
H    -0.66700   1.16940  -0.98430
C     1.17020   0.86020   0.12320
H     1.80940   1.45610   0.77680
C     0.81410  -0.46150   0.80680
H     0.18330  -0.27310   1.67940
C     1.88480   0.55540  -1.16400
C     0.09140  -1.42210  -0.13360
N     1.48760  -0.39730  -1.92760
N     0.40090  -1.12890  -1.54460
C    -1.42070  -1.41820   0.09000
C    -2.31880  -1.15250  -0.93810
C    -3.68220  -1.18590  -0.64680
H    -1.96390  -0.90660  -1.93090
```

C	-3.10670	-1.72600	1.60730
C	-4.09040	-1.48030	0.64810
H	-4.40810	-0.97720	-1.42680
H	-3.38270	-1.95490	2.63370
H	-5.14000	-1.52600	0.91700
C	3.03260	1.38500	-1.59670
C	3.80510	1.03780	-2.71210
C	4.30890	3.24800	-1.20340
C	4.86860	1.85610	-3.06250
C	5.13280	2.98840	-2.29340
H	4.48180	4.12120	-0.57880
H	5.48790	1.61310	-3.92010
H	5.95680	3.65270	-2.52830
N	3.27990	2.47180	-0.85390
N	-1.80310	-1.69920	1.33960
H	3.56410	0.14270	-3.27330
H	0.33920	-1.97060	-2.11660
C	0.66610	-2.87330	0.22730
C	2.06180	-1.22670	1.38290
C	-0.10430	-3.99020	-0.43170
C	-0.85610	-4.90070	0.30690
C	-1.57040	-5.87570	-0.38350
H	-0.87610	-4.83900	1.38740
C	-0.74460	-4.93680	-2.41850
C	-1.51300	-5.90110	-1.77230
H	-2.16470	-6.60370	0.15910
H	-0.68200	-4.91290	-3.50290
H	-2.05400	-6.64350	-2.34780
C	2.99330	-0.31200	2.13590
C	4.27040	-0.01270	1.66910
C	5.03180	0.90590	2.38820
H	4.64840	-0.48150	0.76870
C	3.20030	1.11300	3.90890
C	4.49160	1.47940	3.53250
H	6.03040	1.16850	2.05420
H	2.74160	1.54600	4.79440
H	5.04830	2.19940	4.12140
N	2.45510	0.24180	3.22760
N	-0.05210	-3.99890	-1.76910
N	1.45180	-2.23810	2.27670
N	0.72520	-3.04440	1.68780
N	2.75180	-1.95410	0.30120
N	2.06580	-2.80720	-0.26900

Structure **bis_adduct'_oxi_TSadd1**

S	1.083475	1.753195	-0.961067
C	0.632414	0.035784	-1.416781
H	1.550904	-0.526476	-1.556728
H	0.049472	0.034254	-2.340086
C	-0.212953	-0.506840	-0.282493
C	-1.587339	-0.062161	-0.275012
H	-1.921292	0.504130	-1.141402
C	0.349228	-0.714918	1.067450
C	-2.075141	0.477004	0.995798
N	-0.318855	-0.462761	2.154727
N	-1.488754	0.242529	2.125561
C	-0.406639	-2.533404	-0.895376
C	-2.715788	-1.597862	-0.643304
N	-2.308986	-2.523019	0.333011
N	-1.141892	-2.986494	0.194377
N	-1.113650	-2.313158	-2.074938
N	-2.289656	-1.866124	-1.947401
C	-3.227554	1.418801	1.008503
C	-3.789734	1.839492	2.216864
C	-4.872615	2.706801	2.171644
H	-3.383564	1.478008	3.153430
C	-4.714826	2.652958	-0.213808
C	-5.353279	3.120782	0.932157
H	-5.339577	3.049460	3.089319
H	-5.059134	2.955352	-1.199790
H	-6.202016	3.790212	0.849400
C	1.703794	-1.296869	1.311866
C	1.928992	-2.035737	2.478420
C	3.190605	-2.579642	2.687043
H	1.122751	-2.178530	3.187177
C	3.863063	-1.608764	0.609148
C	4.180903	-2.367270	1.734063
H	3.393491	-3.165746	3.577587
H	4.611965	-1.406203	-0.150499
H	5.177785	-2.777212	1.851694
N	2.657174	-1.078724	0.396655
N	-3.670367	1.824424	-0.185711
C	0.938957	-3.155372	-1.077638
C	1.430490	-4.073075	-0.146512
C	2.681284	-4.634013	-0.375142
H	0.843963	-4.331402	0.726383
C	2.802179	-3.346684	-2.385080
C	3.386232	-4.265707	-1.516605
H	3.097675	-5.346319	0.329852
H	3.321777	-3.035590	-3.287881
H	4.366310	-4.675855	-1.732794
C	-4.143438	-1.151953	-0.539113
C	-4.863250	-0.804372	-1.681998
C	-5.891512	-0.643209	0.835445
C	-6.168795	-0.355490	-1.521292
H	-4.405846	-0.881594	-2.660302
C	-6.695318	-0.263065	-0.237325
H	-6.266842	-0.582279	1.854087
H	-6.761125	-0.073606	-2.385702
H	-7.703928	0.095007	-0.063866
N	-4.641126	-1.086275	0.696602
N	1.602647	-2.800593	-2.181750
C	2.844602	1.787103	-1.443354
H	2.945245	2.023341	-2.504400
H	3.274407	0.804770	-1.231028
C	3.598771	2.829109	-0.598378
H	4.616149	2.895275	-0.997084
C	2.936409	4.191250	-0.716667
C	4.540021	1.517889	1.219701
O	2.377015	4.756232	0.194711
O	3.007242	4.652473	-1.961401
C	2.337148	5.898972	-2.196645
H	2.485195	6.117745	-3.251503
H	1.274724	5.798986	-1.967975
H	2.774642	6.681505	-1.574915
N	3.668674	2.467101	0.792924
O	5.372094	1.023640	0.465289
C	4.385629	1.104401	2.664973
H	5.284488	0.577153	2.983640
H	4.205817	1.964240	3.314903
H	3.528815	0.426072	2.750403
H	2.969636	2.853438	1.414651

3. Spectroscopic data

NMR spectra

^{1}H NMR ($CDCl_3$) of BocCysOMe-S-allyl

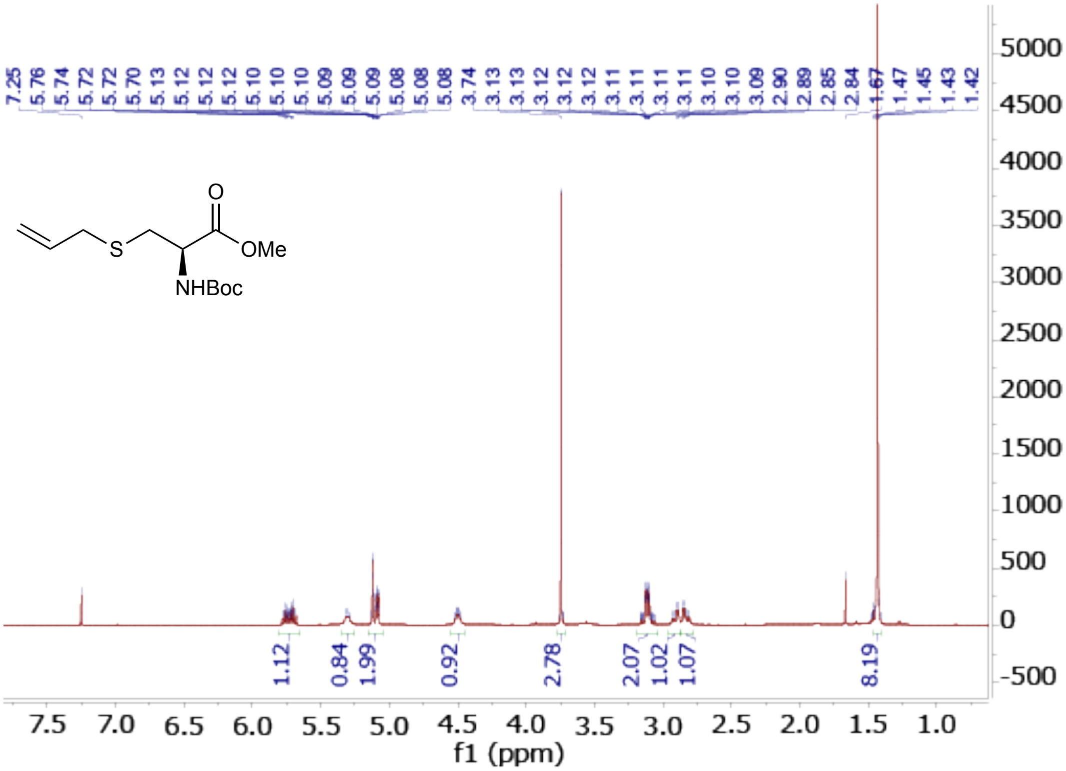

^{13}C NMR ($CDCl_3$) of BocCysOMe-S-allyl

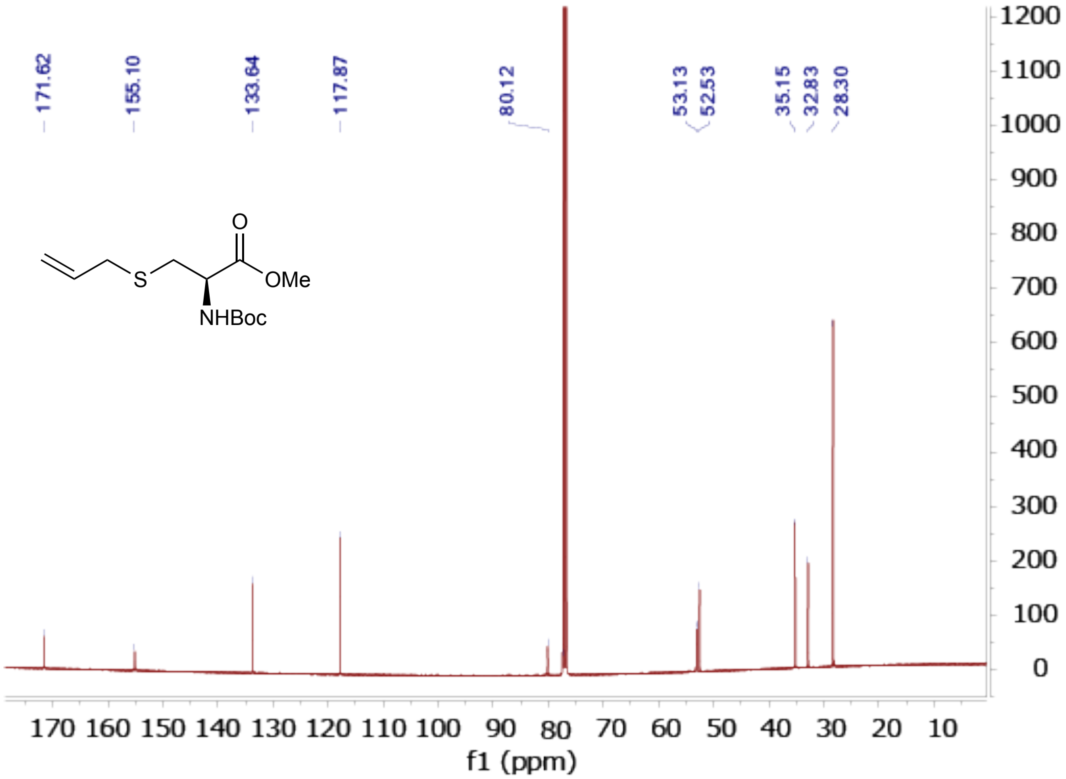

^{1}H NMR (D_2O) of ***1***

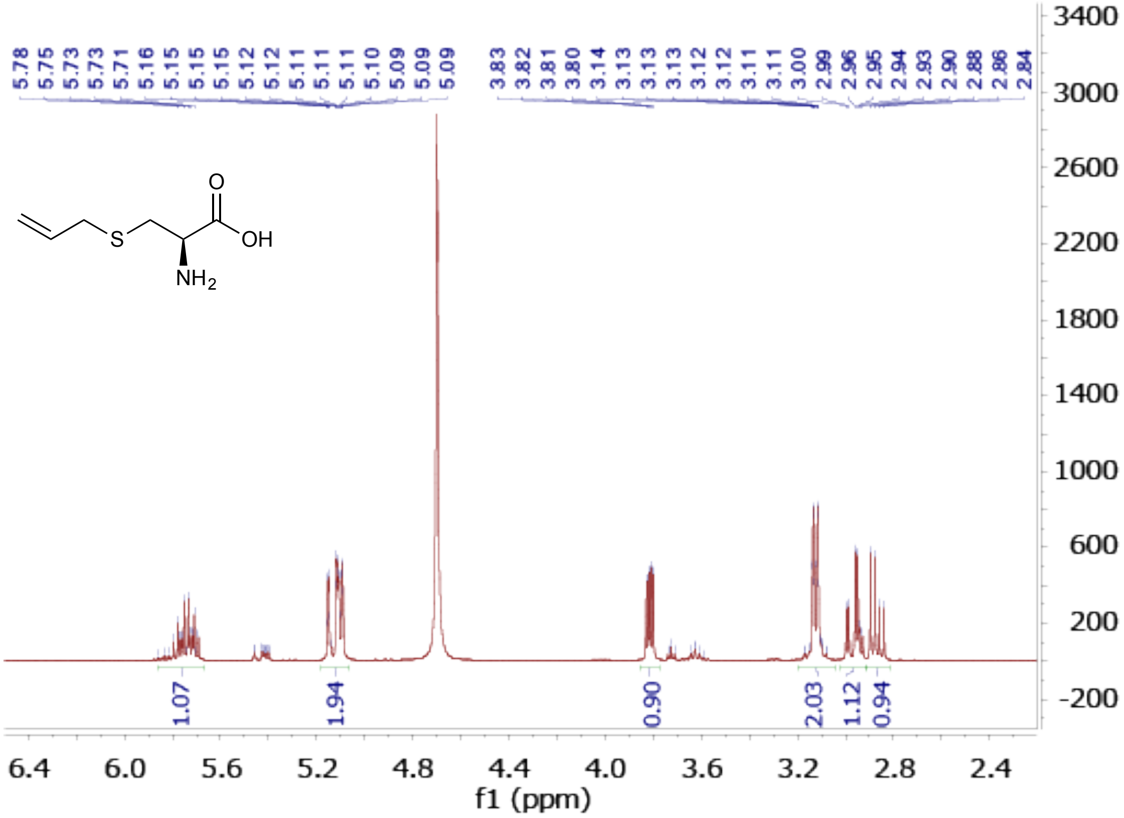

^{13}C NMR (D_2O) of ***1***

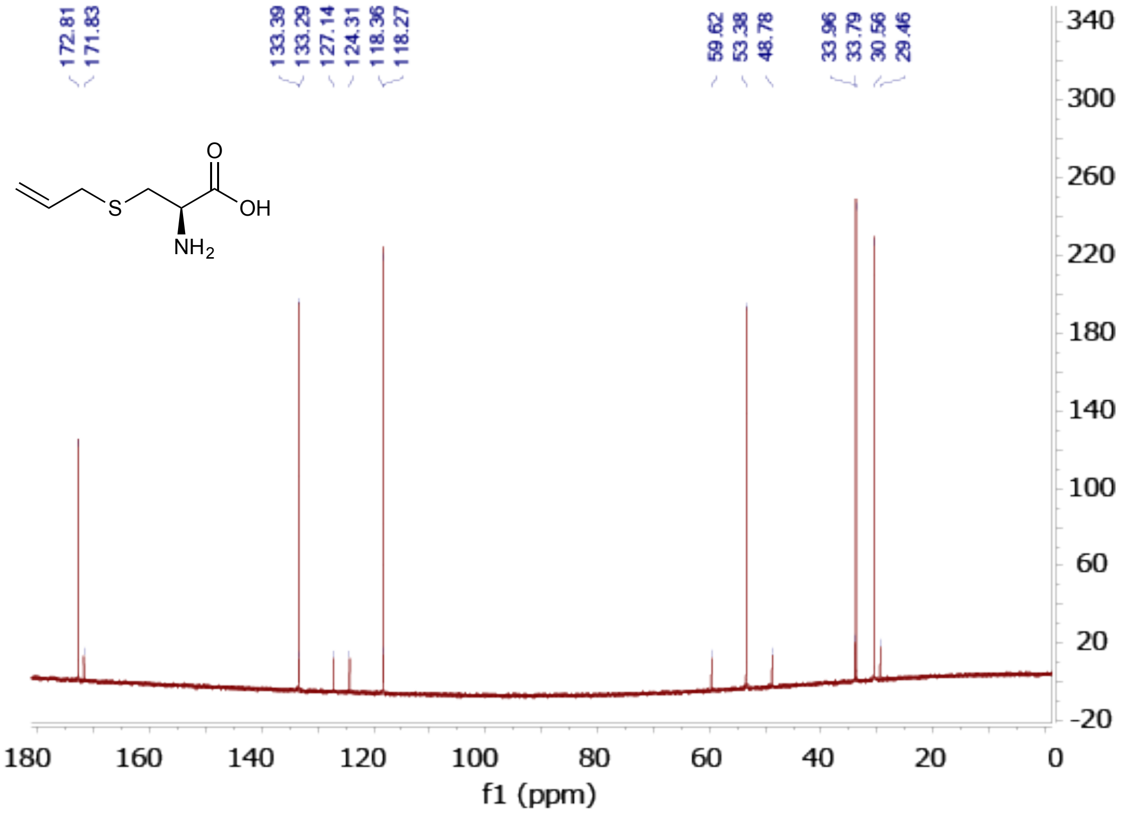

^{1}H NMR ($CDCl_3$) of Cys-Py-Tz_{red}

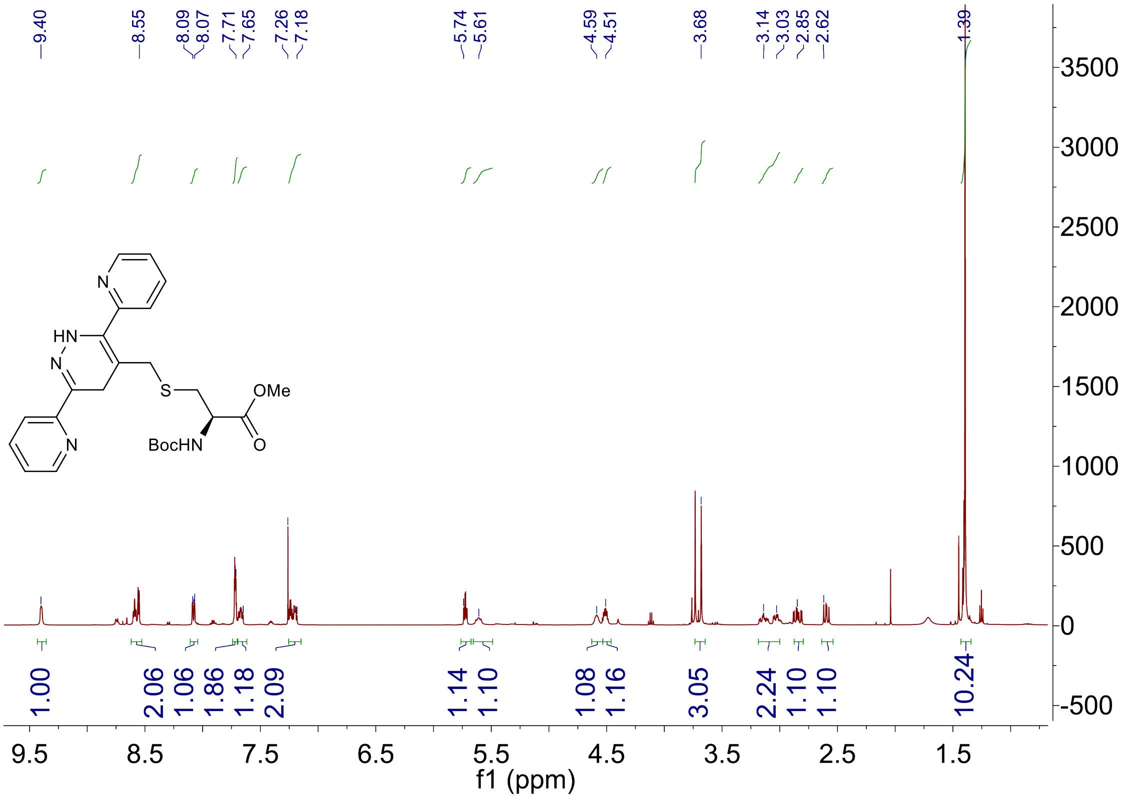

^{13} C NMR ($CDCl_3$) of Cys-Py-Tz_{red}

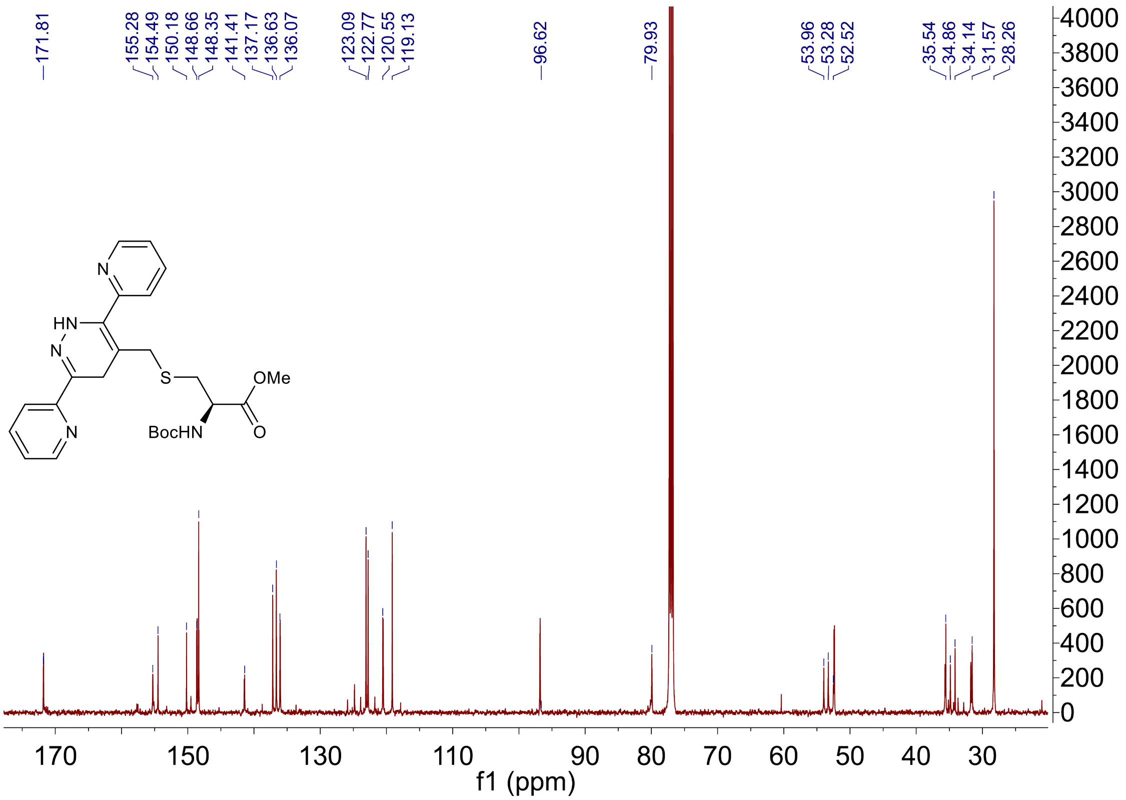

^{1}H NMR ($CDCl_3$) of Cys-Py-Tz$_{oxi}$

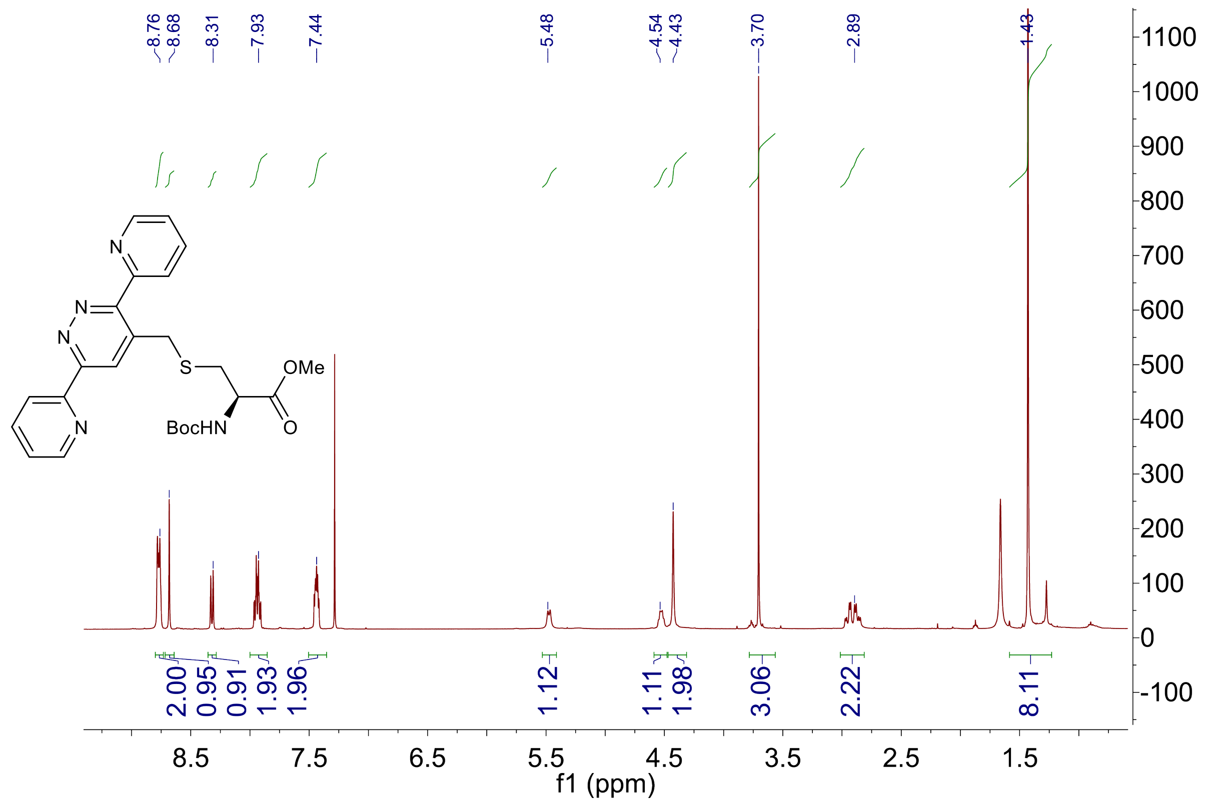

^{13} C NMR ($CDCl_3$) of Cys-Py-Tz$_{oxi}$

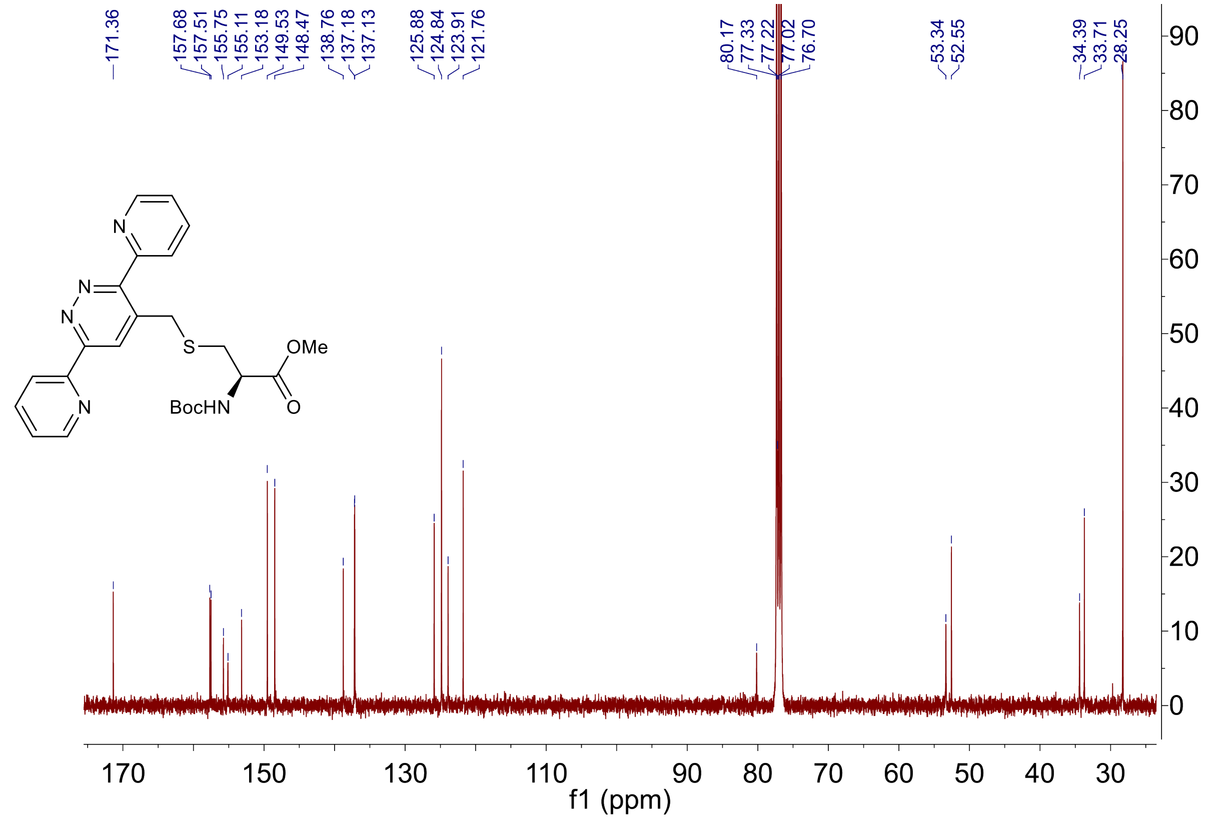

^{1}H NMR (CDCl3) of bis-addition product

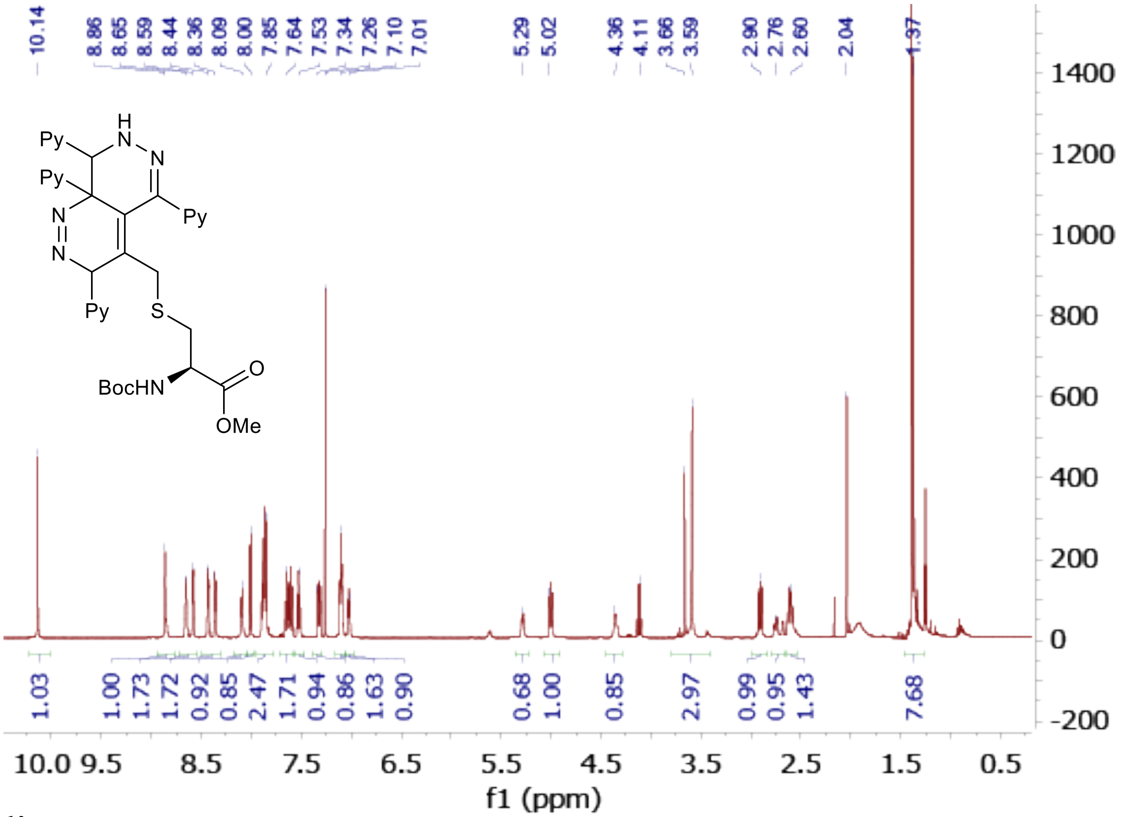

^{13}C NMR (CDCl3) of bis-addition product

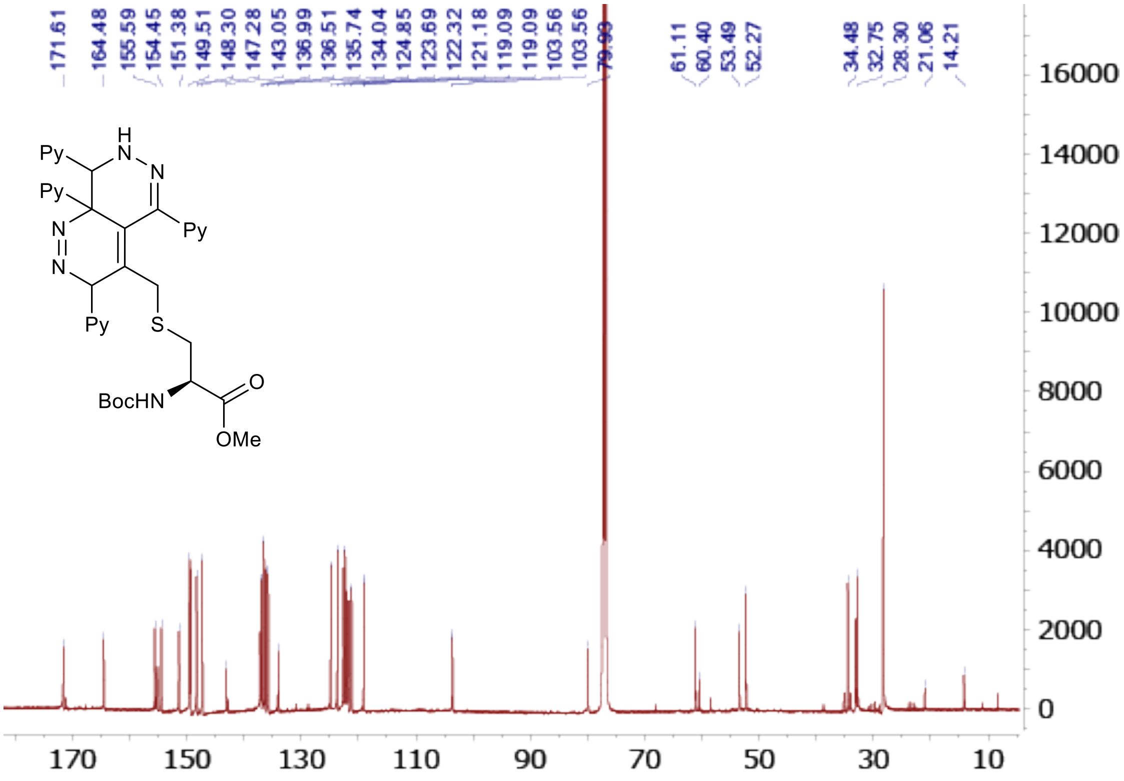

^{1}H NMR (CD_3OD) of Rhod-NH_2

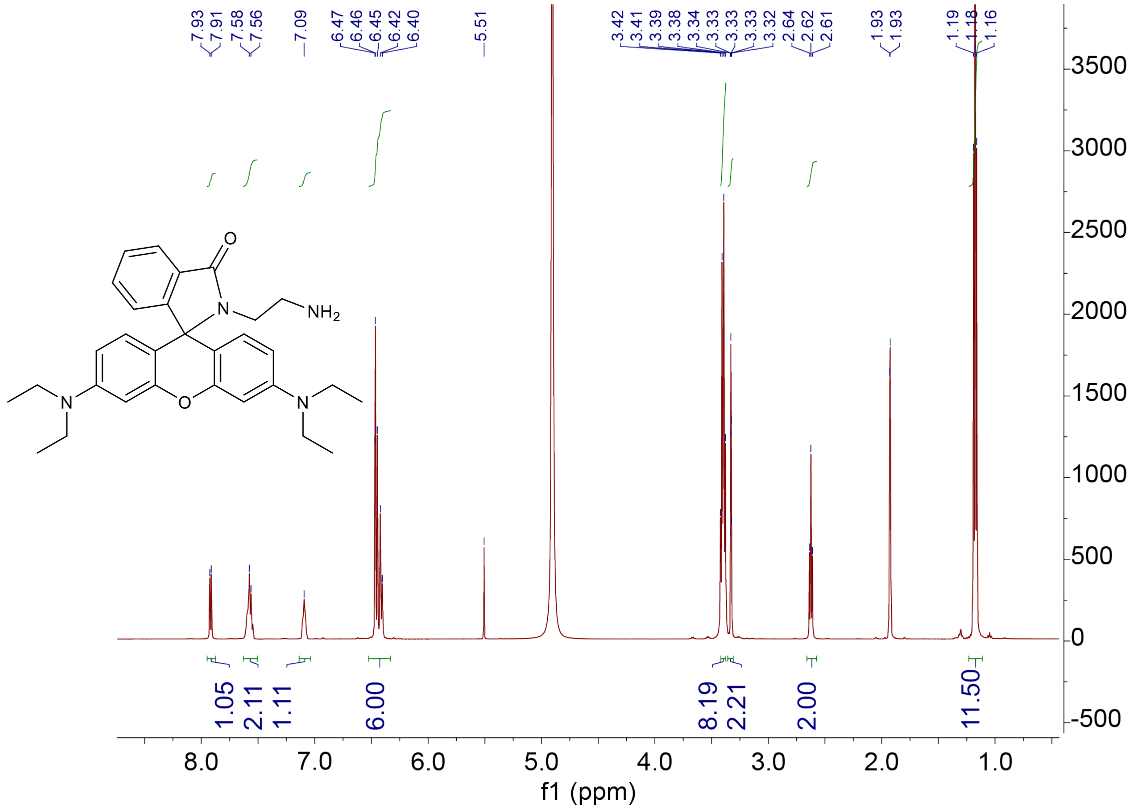

^{1}H NMR (d_6-DMSO) of Tz-NH_2-oxi

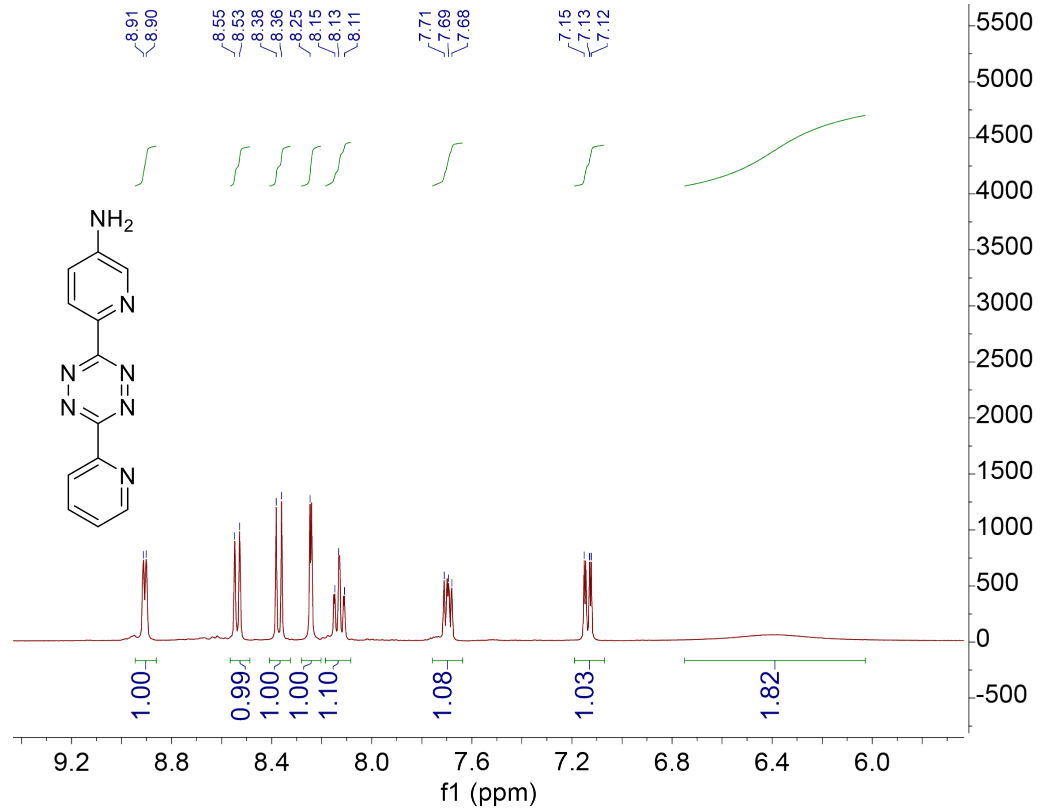

^{1}H NMR (d_6-DMSO) of Py-Tz-NH2-succinic

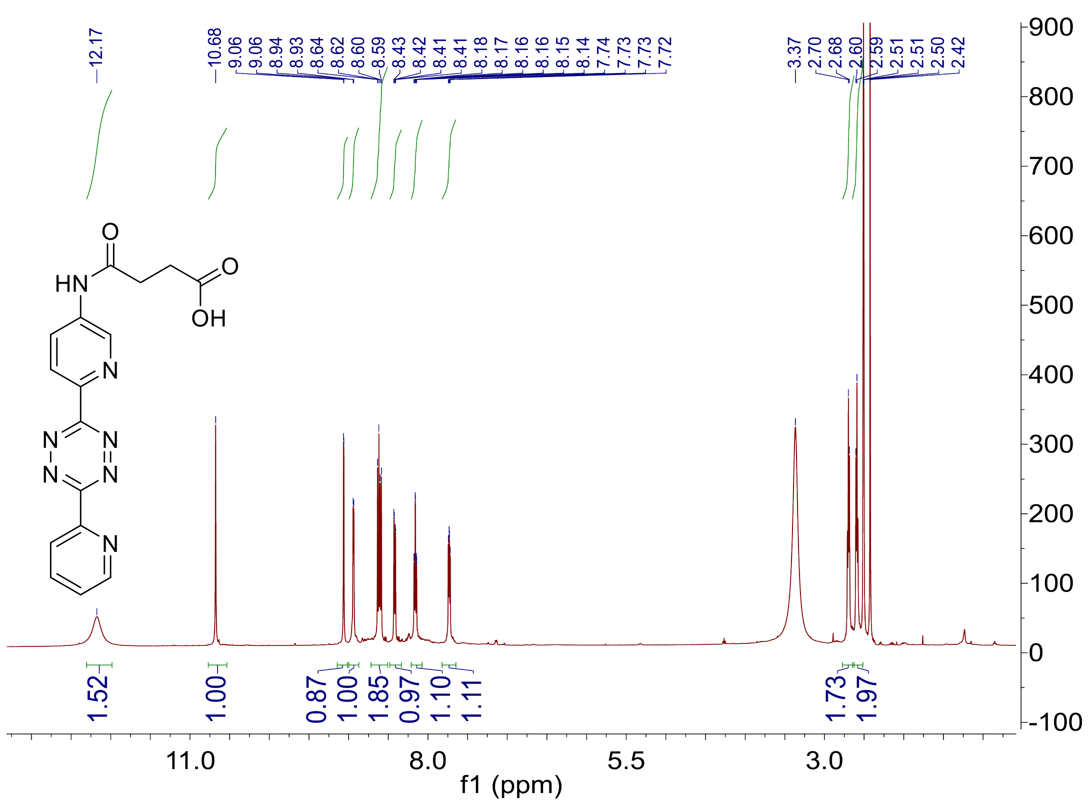

^{1}H NMR ($CDCl_3$) of Tz-Rhod 4

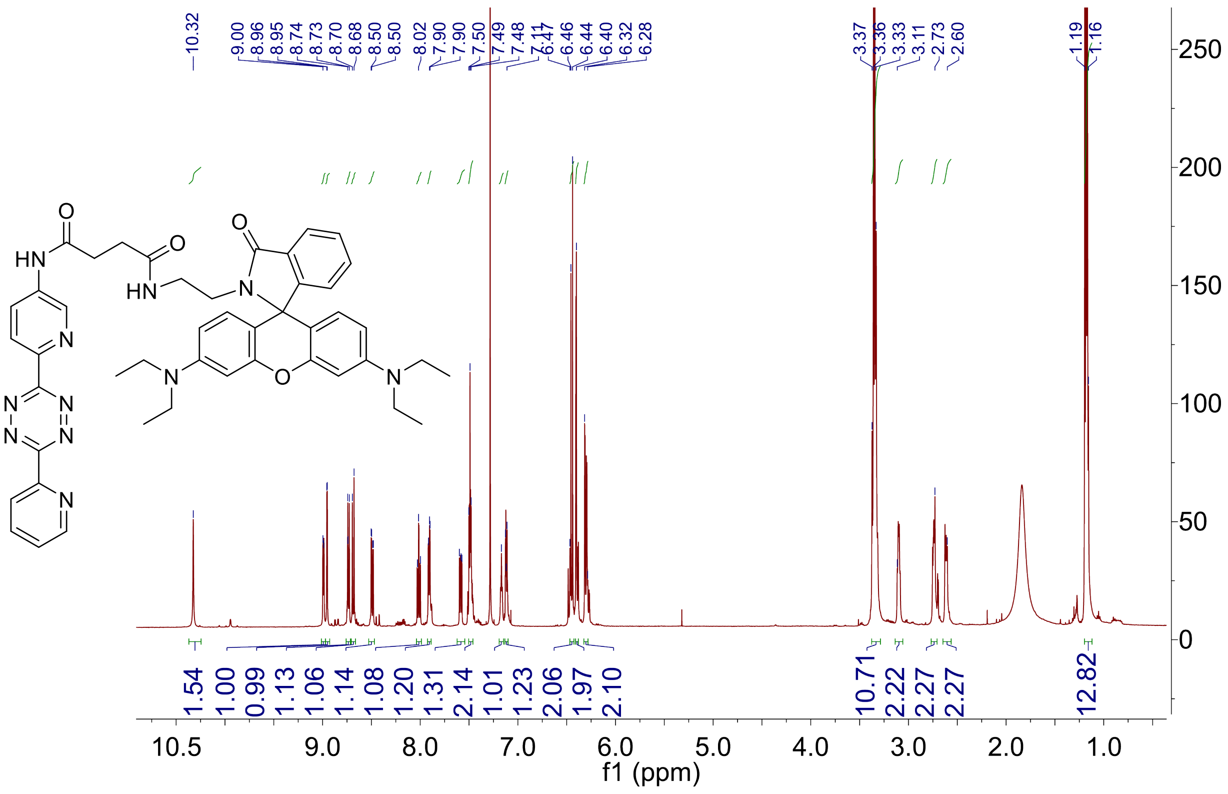

^{13}C NMR (CDCl$_3$) of Tz-Rhod 4

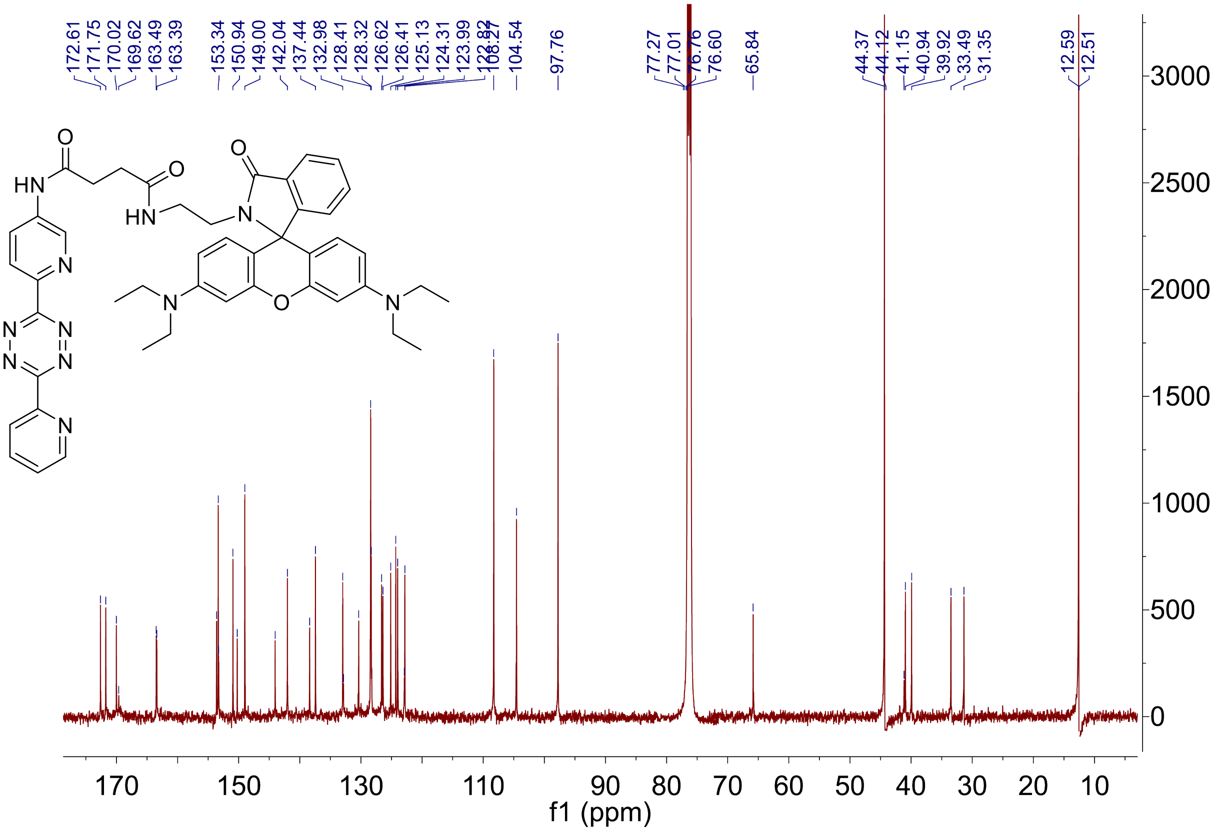

Excitation and fluorescence emission of Tz-Rhod 4 (PBS buffer pH 7.6, 0.1 mM)

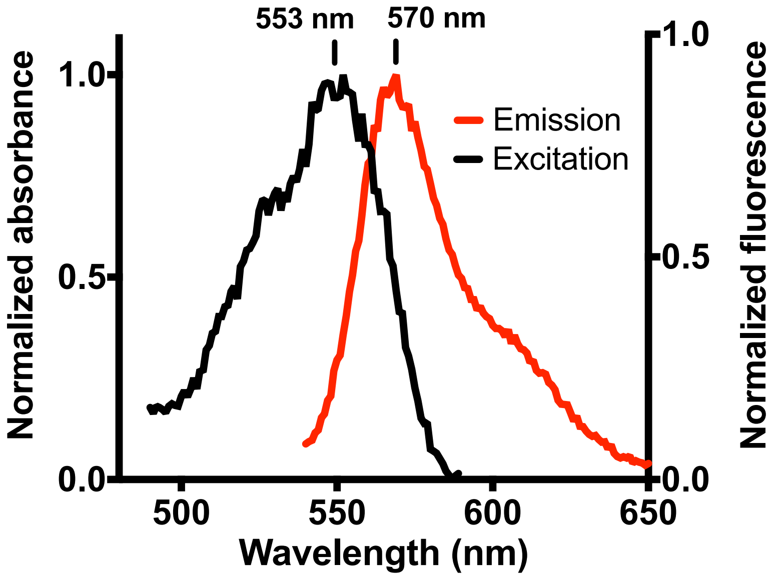

4. References

[1] X. Zhang, Y. Shiraishi, T. Hirai, *Org. Lett.* **2007**, *9*, 5039.

[2] M. Jin, C. Smith, H.-Y. Hsieh, D. F. Gibson, J. F. Tait, *J. Biol. Chem.* **2004**, *279*, 40351.

[3] I. S. Alam, A. A. Neves, T. H. Witney, J. Boren, K. M. Brindle, *Bioconjugate Chem.* **2010**, *21*, 884.

[4] Frisch, M. J.; Trucks, G. W.; Schlegel, H. B.; Scuseria, G. E.; Robb, M. A.; Cheeseman, J. R.; Scalmani, G.; Barone, V.; Mennucci, B.; Petersson, G. A.; Nakatsuji, H.; Caricato, M.; Li, X.; Hratchian, H. P.; Izmaylov, A. F.; Bloino, J.; Zheng, G.; Sonnenberg, J. L.; Hada, M.; Ehara, M.; Toyota, K.; Fukuda, R.; Hasegawa, J.; Ishida, M.; Nakajima, T.; Honda, Y.; Kitao, O.; Nakai, H.; Vreven, T.; J. A. Montgomery, J.; Peralta, J. E.; Ogliaro, F.; Bearpark, M.; Heyd, J. J.; Brothers, E.; Kudin, K. N.; Staroverov, V. N.; Kobayashi, R.; Normand, J.; Raghavachari, K.; Rendell, A.; Burant, J. C.; Iyengar, S. S.; Tomasi, J.; Cossi, M.; Rega, N.; Millam, J. M.; Klene, M.; Knox, J. E.; Cross, J. B.; Bakken, V.; Adamo, C.; Jaramillo, J.; Gomperts, R.; Stratmann, R. E.; Yazyev, O.; Austin, A. J.; Cammi, R.; Pomelli, C.; Ochterski, J. W.; Martin, R. L.; Morokuma, K.; Zakrzewski, V. G.; Voth, G. A.; Salvador, P.; Dannenberg, J. J.; Dapprich, S.; Daniels, A. D.; Farkas, Ö.; Foresman, J. B.; Ortiz, J. V.; Cioslowski, J.; Fox, D. J.; Gaussian, Inc.: Wallingford CT, 2009.

[5] Zhao, Y.; Truhlar, D. *Theor. Chem. Acc.* **2008**, *120*, 215.

[6] Scalmani, G.; Frisch, M. J. *J. Chem. Phys.* **2010**, *132*.

[7] Gonzalez, C.; Schlegel, H. B. *J. Chem. Phys.* **1989**, *90*, 2154.

[8] Gonzalez, C.; Schlegel, H. B. *J. Phys. Chem.* **1990**, *94*, 5523.
